# Model-Informed Drug Development, Pharmacokinetic/Pharmacodynamic Cutoff Value Determination, and Antibacterial Efficacy of Benapenem against *Enterobacteriaceae*

**DOI:** 10.1128/AAC.01751-19

**Published:** 2020-02-21

**Authors:** Xi-wei Ji, Feng Xue, Zi-sheng Kang, Wei Zhong, Isabelle Hui-san Kuan, Xi-ping Yang, Xiao Zhu, Yun Li, Yuan Lv

**Affiliations:** a Institute of Clinical Pharmacology, Peking University First Hospital, Beijing, China; b School of Pharmacy, University of Otago, Dunedin, New Zealand; c XuanZhu Pharma Co., Ltd., Jinan, Shandong, China

**Keywords:** PK/PD cutoff values, benapenem, model-informed drug development, simulation, *Enterobacteriaceae*, Monte Carlo simulation

## Abstract

Benapenem is a novel carbapenem. The objective of this study was to determine the pharmacokinetic (PK)/pharmacodynamic (PD) cutoff values and evaluate the optimal administration regimens of benapenem for the treatment of bacterial infections via PK/PD modeling and simulation. Ertapenem was used as a control. Infected mice received an intravenous (i.v.) injection of benapenem or ertapenem of 14.6, 58.4, or 233.6 mg/kg of body weight, and the PK/PD profiles were evaluated. The MICs were determined by using a 2-fold agar dilution method. Mathematical models were developed to characterize the pharmacokinetic profile of benapenem in humans and mice. Monte Carlo simulations were employed to determine the cutoff values and the appropriate benapenem dosing regimens for the treatment of infections caused by clinical isolates of *Enterobacteriaceae*. Two 2-compartment models were developed to describe the PK profiles of benapenem in humans and mice. A two-site binding model was applied to fit the protein binding in mouse plasma. Through correlation analysis, the percentage of the time that the free drug concentration remains above the MIC (%*fT*_>MIC_) was determined to be the indicator of efficacy. Results from the simulation showed that the probability of target attainment (PTA) against the tested isolates was over 90% with the dosing regimens studied. The PK/PD cutoff value of benapenem was 1 mg/liter at a %*fT*_>MIC_ of 60% when given at a dose of 1,000 mg/day by i.v. drip for 0.5 h. The established model provides a better understanding of the pharmacological properties of benapenem for the treatment of *Enterobacteriaceae* infections. The proposed PK/PD cutoff value suggests that benapenem is a promising antibacterial against the *Enterobacteriaceae*. The cutoff value of 1 mg/liter may be a useful guide for the clinical use of benapenem and for surveillance for benapenem resistance.

## Abstract (precis)

Benapenem is a novel carbapenem. The objective of this study was to determine the pharmacokinetic (PK)/pharmacodynamic (PD) cutoff values and evaluate the optimal administration regimens of benapenem for the treatment of bacterial infections via PK/PD modeling and simulation. Ertapenem was used as a control. Infected mice received an intravenous (i.v.) injection of benapenem or ertapenem of 14.6, 58.4, or 233.6 mg/kg of body weight, and the PK/PD profiles were evaluated.

## INTRODUCTION

Benapenem is a novel parenteral beta-lactam antibacterial that has potential for use for the treatment of severe infections. It exerts clinical effects as a carbapenem and has the potential to treat severe infections, such as intra-abdominal infections, soft tissue infections, complicated urinary tract infections, and community-acquired pneumonia. It has been shown to have excellent antibacterial activity against a wide variety of bacteria, including extended-spectrum-beta-lactamase-(ESBL)-positive, Gram-negative, and anaerobic bacteria (1). Benapenem works by binding to penicillin-binding proteins (PBPs), thus interfering with the lengthening and strengthening (added through cross-linking) of the peptidoglycan portion of the bacterial cell wall (i.e., inhibiting bacterial cell wall synthesis). For Gram-negative bacteria, beta-lactams gain access to the PBPs by first moving through porin channels to the periplasmic space, withstanding potential degradation and binding to the PBPs.

Breakpoints are important for clinical antibacterial selection, an indispensable part of microbiology laboratory practice used to define susceptibility, intermediate, or resistance to antibacterials. The breakpoints can be classified into epidemiological cutoff values (Ecoff), pharmacokinetic (PK)/pharmacodynamic (PD) cutoff values, and clinical breakpoints. PK/PD cutoff values refer to the antibacterial concentrations (i.e., MICs) calculated from knowledge of a PK/PD index and the dimension of that parameter that predicts efficacy *in vivo*. The data derived from preclinical studies, such as animal experiments, can be extrapolated to humans through mathematical or statistical techniques (2–4).

Model-informed drug development (MIDD) is increasingly considered a key component of modern drug development and applies a number of models (including population pharmacokinetic [Pop-PK] models, PK/PD models, exposure-response models, etc.) derived from preclinical and clinical data sources to address drug development or promote the decision-making process (5–7). In our study, the MIDD approaches were utilized with the aim of optimizing benefit-risk and improving the efficiency of benapenem development.

The objective of this study was to determine the PK/PD index, target value, and PK/PD cutoff values of benapenem against *Enterobacteriaceae* through PK/PD analysis and, ultimately, to propose the sensitivity breakpoint together with Ecoff. The efficacy of the dosing regimens of benapenem tested against infections caused by *Enterobacteriaceae* was investigated. The *in vitro* and *in vivo* antibacterial efficacy of benapenem was also evaluated in the preclinical study.

## RESULTS

### PK data and plasma protein binding rates of benapenem and ertapenem.

The demographic data from the benapenem clinical phase I trial are listed in Table 1. The protein binding rates of benapenem and ertapenem in mouse plasma and human plasma gradually became saturated with an increase in their concentrations. The plasma protein binding rates of benapenem and ertapenem decreased to less than 50% at 1,000 μM (see Table S2 in the supplemental material).

**TABLE 1:** Demographic data from benapenem clinical phase I trial

Attribute	Value
No. of patients	12
No. of females/no. of males	6/6
Median (range) age (yr)	28 (21–35)
Median (range) body wt (kg)	61.5 (51.8–78.3)
Median (range) body ht (cm)	165.3 (153.5–181)

### *In vitro* activity of benapenem and ertapenem.

The *in vitro* activity of benapenem and ertapenem is shown in Table 2. The MIC_50_ and MIC_90_ of benapenem against most tested bacteria were ≤0.125 μg/ml and ≤0.5 μg/ml, respectively. Benapenem displayed potent activity against ESBL-producing (ESBL^+^) and ESBL-nonproducing (ESBL^−^) Escherichia coli, ESBL-producing Klebsiella pneumoniae, and Enterobacter cloacae strains.

**TABLE 2:** MICs of benapenem and ertapenem against clinical isolates of *Enterobacteriaceae*

Species	No. of strains	MIC (μg/ml)			
		Benapenem		Ertapenem	
		50%	90%	50%	90%
ESBL^+^ E. coli	41	0.031	0.25	0.031	0.5
ESBL^−^ E. coli	30	0.016	0.031	0.008	0.008
ESBL^+^ K. pneumoniae	38	0.062	0.25	0.062	0.5
ESBL^−^ K. pneumoniae	31	0.031	0.031	0.008	0.008
Klebsiella aerogenes	16	0.125	0.5	0.062	0.5
Other *Klebsiella* spp.	16	0.016	0.062	0.008	0.008
Enterobacter cloacae	21	0.125	1	0.25	1
*Citrobacter* spp.	16	0.062	0.5	0.062	0.25
*Serratia* spp.	15	0.125	0.25	0.031	0.25
*Proteus* spp.	23	0.062	0.125	0.016	0.016
*Morganella* spp.	12	0.125	0.25	0.016	0.031
*Salmonella* spp.	16	0.031	0.062	0.008	0.062
*Shigella* spp.	16	0.031	0.031	0.008	0.031
*Providencia*	21	0.125	4	0.016	0.5

### PK/PD model and mouse and human Pop-PK models.

The pharmacokinetic characteristics of benapenem in mice were described by a two-compartment model with dosage as the covariate (equations 1 and 2):

(1) $$\text{CL}=0.913\cdot \left[1+{\left(\text{dose}/58.4\right)}^{1.46}\right]\cdot e^{{\text{η}}_{1}}$$

(2) $$V_{1}=3.62\cdot \left[1+{\left(\text{dose}/58.4\right)}^{1.06}\right]$$

where CL is clearance (in milliliters per hour), *V*_1_ is the volume of the central compartment (in hours), η_1_ is the interindividual variation, intercompartmental clearance (*Q*) is equal to 0.09 ml/h, and the volume of the peripheral compartment (*V*_2_) is equal to 1.65 ml. The pharmacokinetic properties of benapenem in humans were profiled by a two-compartment model with dosage and body weight as covariates (equations 3 and 4):

(3) $$\text{CL}=0.825\cdot {\left(\text{dose}/250\right)}^{0.183}\cdot e^{{\text{η}}_{1}}$$

(4) $$V_{1}=5.03\cdot {\left(\text{dose}/250\right)}^{0.184}\cdot \left(\text{WT}/70\right)\cdot e^{{\text{η}}_{2}}$$

where CL is in liters per hour, *V*_1_ is in liters, η_2_ is the interindividual variation, *Q* is equal to 1.14 liter/h, *V*_2_ is equal to 2.93 liters, and the infusion duration is 0.404 h.

**FIG 1:**
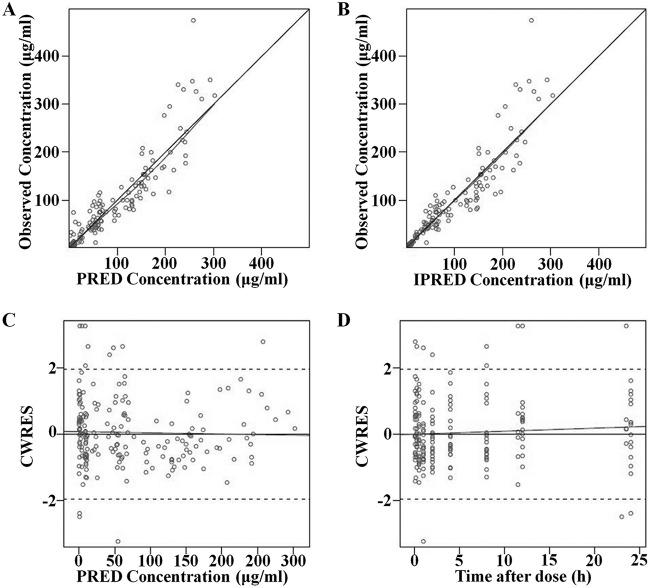
Goodness-of-fit plots of the mouse PK model. (A) Relationship between observed and predicted (PRED) PK values; (B) relationship between observed and individual predicted (IPRED) PK values; (C) Conditional weighted residuals (CWRES) at different predicted values; (D) CWRES at different time points. The solid lines represent *x* equal to* y*. The dotted lines are trend lines.

**FIG 2:**
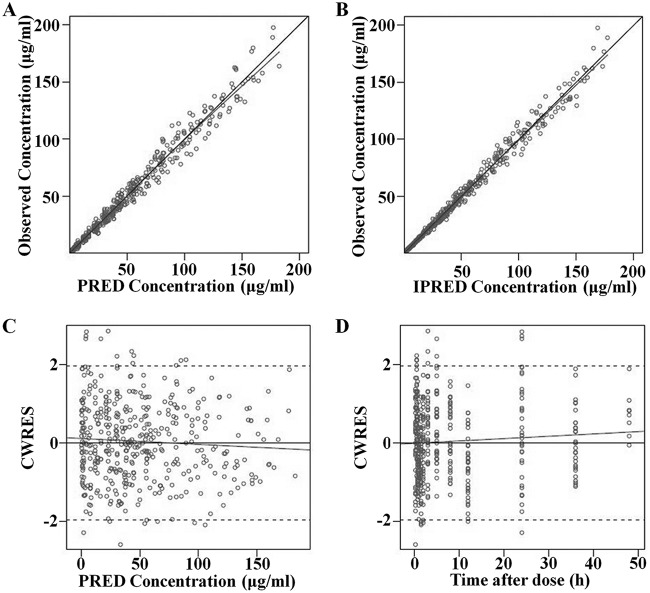
Goodness-of-fit plots of human PK model. (A) Relationship between observed and predicted (PRED) PK values; (B) relationship between observed and individual predicted (IPRED) PK values; (C) Conditional weighted residuals (CWRES) at different predicted values; (D) CWRES at different time points. The solid lines represent *x* equal to* y*. The dotted lines are trend lines.

**FIG 3:**
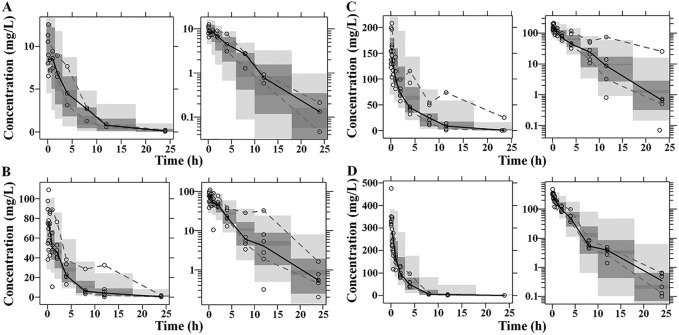
Visual predictive check (VPC) of mouse PK model. (Left) Arithmetic scale; (right) logarithmic scale. The results are for the 1.9-mg (A), 14.6-mg (B), 58.4-mg (C), and 233-mg (D) dose groups. The range between the dashed lines depicts the 90th percentile intervals. The solid lines represent the medians of the simulated data. Circles represent the observed data.

**FIG 4:**
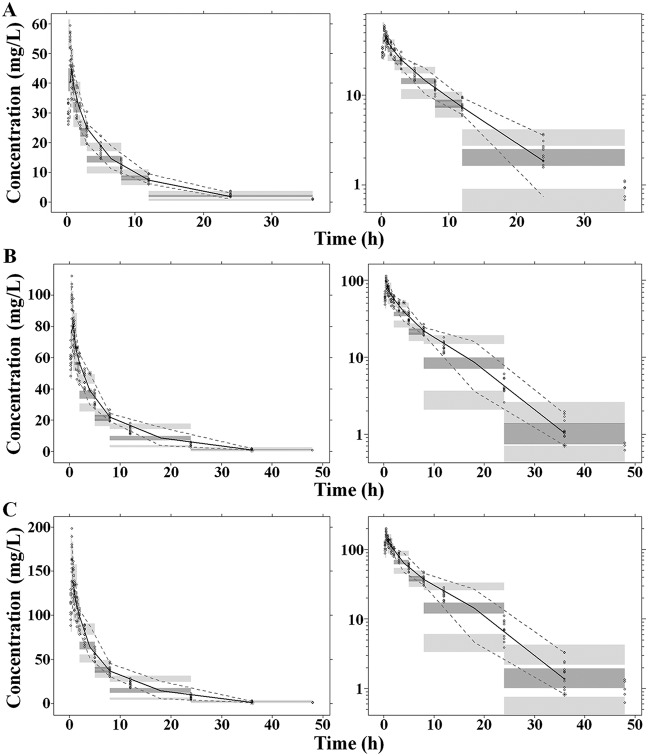
Visual predictive check (VPC) of human PK model. (Left) arithmetic scale; (right) logarithmic scale), The results are for the 250-mg (A), 500-mg (B), and 1,000-mg (C) dose groups. The range between the dashed lines depicts the 90th percentile intervals. The solid lines represent the medians of the simulated data. Circles represent the observed data.

The diagnostic goodness-of fit plots for the mouse and human PK models are shown in Fig. 1 and 2, respectively. The observed values versus either the population or the individual predicted values were closely distributed around the line of identity (Fig. 1A and B and 2A and B). The conditional weighted residuals (CWRES) were randomly and homogeneously distributed near 0 (Fig. 1C and D and 2C and D). The visual predictive check (VPC) results for the mouse (Fig. 3) and human (Fig. 4) PK models indicated that the established models were able to describe the data well, with most of the observed plasma concentrations falling within the 90% prediction intervals. Therefore, the models adequately predicted the observed PK profile of benapenem.

The model parameters for the mouse and human Pop-PK models are summarized in Tables 3 and 4, respectively, and suggest that the model demonstrates acceptable predictability.

**TABLE 3:** Parameters estimates obtained from the mouse Pop-PK model of benapenem

Value (% RSE) for the following parameter^*a*^:								
CL (ml/h)	*V*_1_ (ml)	*Q* (ml/h)	*V*_2_ (ml)	Dose_CL	Dose_*V*_1_	IIV_CL	Prop. error (%)	Add. error
0.913 (6)	3.62 (3)	0.09 (36)	1.65 (34)	1.46 (4)	1.06 (5)	36.5 (15)^*b*^	30.1 (7)	0 (fix)

a RSE, relative standard error; CL, clearance; *V*_1_, volume of central compartment; *Q*, intercompartmental clearance; *V*_2_, volume of peripheral compartment; Dose_CL, dose effect on CL; Dose_*V*_1_, dose effect on *V*_1_; IIV _CL, interindividual variation of CL; Prop. error, proportional residual error; Add. error, additive residual error.

b The eta shrinkage for IIV_CL was 11.

**TABLE 4:** Parameters estimates obtained from the human Pop-PK model of benapenem

Value (% RSE) for the following parameter^*a*^:										
CL (liters/h)	*V*_1_ (liters)	*Q* (liters/h)	*V*_2_ (liters)	*D*_1_ (h)	Dose_CL	Dose_*V*_1_	IIV_CL	IIV_*V*_1_	Prop. error (%)	Add. error
0.825 (3)	5.03 (2)	1.14 (8)	2.93 (4)	0.404 (1)	0.183 (8)	0.184 (7)	10.5 (15)	7.6 (16)	7.4 (4)	0.233 (15)

a RSE, relative standard error; CL, clearance; *V*_1_, volume of central compartment; *Q*, intercompartmental clearance; *V*_2_, volume of peripheral compartment; *D*_1_, intravenous infusion time; Dose_CL, dose effect on CL; Dose_*V*_1_, dose effect on *V*_1_; IIV _CL, interindividual variation of CL; IIV _*V*_1_, interindividual variation of *V*_1_; Prop. error, proportional residual error; Add. error, additive residual error. The eta shrinkage for IIV_CL was −3%, and the eta shrinkage for IIV_*V*_1_ was 0%.

### Covariate analysis.

The influences of covariates on the model parameters were assessed through stepwise regression analysis. Dosage as a covariate was found to have an effect on clearance and the volume of the central compartment in the mouse population PK model. In the human population PK model, the volume of the central compartment was also significantly affected by body weight.

### Determination of free benapenem concentration.

**FIG 5:**
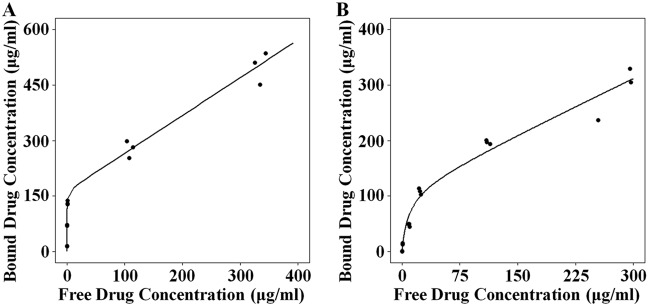
Observed and predicted plasma protein binding profiles of mice (A) and humans (B). The solid lines represent the simulated data. Circles represent the observed data.

The simplified two-site binding model appropriately described the protein binding of benapenem to mouse and human plasma proteins at multiple concentrations (Fig. 5A and B). The corresponding model parameters are listed in Table 5. All parameters were estimated with good precision (relative standard error [RSE] ≤ 20%).

**TABLE 5:** Parameter estimates obtained from the plasma protein binding model

Species	Value (% RSE) for the following parameter^*a*^:			
	*B*_max_ (μg/ml)	*C*_50_ (μg/ml)	Slope	Prop. error (%)
Mouse	164 (3)	0.232 (6)	1.02 (5)	5.4 (18)
Human	115 (15)	8.63 (16)	0.666 (15)	16.3 (14)

a RSE, relative standard error; *B*_max_, maximum load in the binding sites; *C*_50_, apparent dissociation constant; Prop. error, proportional residual error.

### PK/PD correlation analyses of benapenem and determination of PK/PD index.

**FIG 6:**
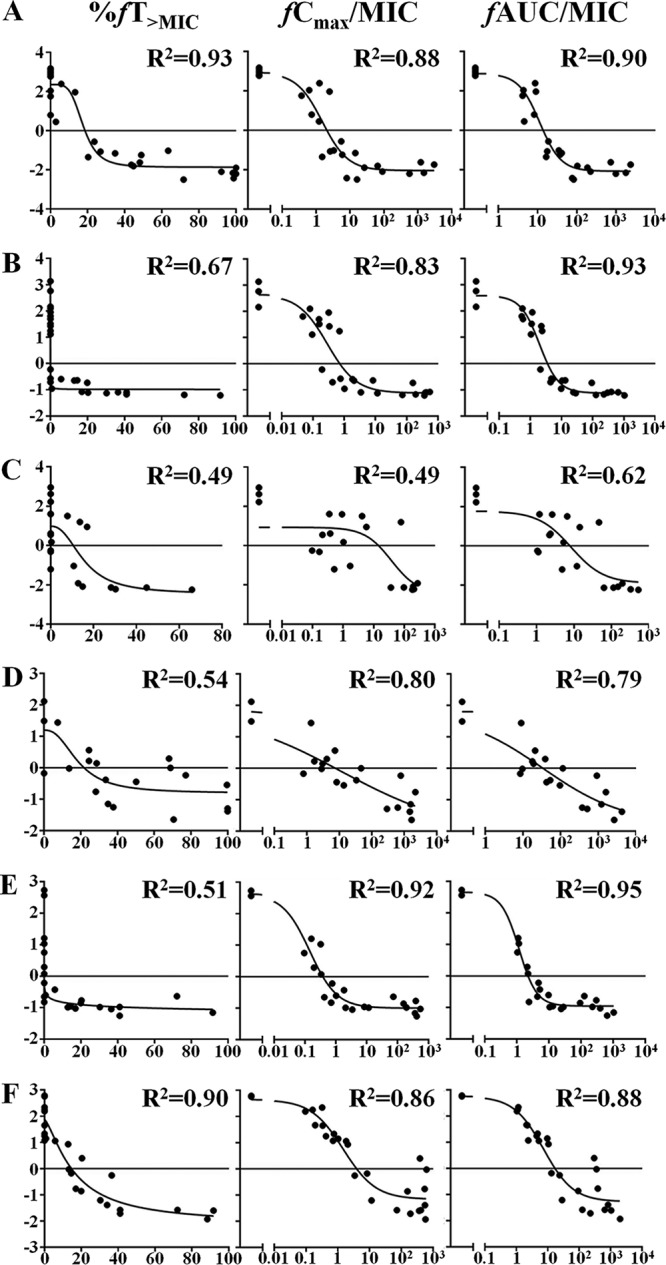
Correlation between antibacterial effects and %*fT*_>MIC_ *fC*_max_/MIC, or *f*AUC/MIC. (A) ATCC 25922 (ESBL^−^ E. coli); (B) 13G136 (ESBL^+^ E. coli); (C) 7742692 (ESBL^+^ E. coli); (D) ATCC 700603 (ESBL^+^ K. pneumoniae); (E) 13C285 (ESBL^+^ K. pneumoniae); (F) 13H279 (Enterobacter cloacae). The dotted line represents the linear correlation; the solid line demarcates the antibacterial effects.

As shown in Fig. 6, the correlations between the PK/PD indexes and the number of log(CFU per gram) [log(CFU/g)] are approximate. The correlation between the percentage of the time that the free drug concentration remains above the MIC (%*fT*_>MIC_) and log(CFU/g) was greater than that between the maximum concentration in plasma (*C*_max_)/MIC and the area under the concentration-time curve (AUC)/MIC for two strains, 13H279 and ATCC 25922, although the correlation between AUC/MIC and log(CFU/g) was the largest, in general. By comprehensive consideration of the results of the preclinical study and the mode of action of other carbapenems (8–10), %*fT*_>MIC_ was ultimately used as the PK/PD index in this study.

### PK/PD analysis of benapenem and ertapenem in infected mice and determination of the target value.

The results from this study showed that increasing values of the PK/PD index (%*fT*_>MIC_) led to improved efficacy {change in log(CFU/g) [Δlog(CFU/g)]}. The results of the analysis describing the relationship between %*fT*_>MIC_ and the antibacterial effects of benapenem and ertapenem are shown in Fig. S1 to S12. Two key values of %*fT*_>MIC_ can be derived: (i) the value that results in a bacteriostatic effect [no drop in log(CFU/g), i.e., Δlog(CFU/g) = 0] and (ii) the value that results in a bactericidal effect [a 1- or 2-log drop in log(CFU/g), i.e., Δlog(CFU/g) = −1 or −2] (11). These are defined as the target values. The target values of benapenem against E. coli, K. pneumoniae, and E. cloacae resulting in a bacteriostatic effect were 6% to 36%, 2% to 18%, and 17%, respectively (Table 6). The Δlog(CFU/g) values were between −1 and −2 when benapenem exerted maximum efficacy against the other strains tested, except for strain 7742692 (an ESBL^+^ E. coli strain). For the 7742692 strain (an ESBL^+^ E. coli strain), a maximum effect was reached when Δlog(CFU/g) was −2. The target values of benapenem resulting in a bactericidal effect against E. coli, K. pneumoniae, and E. cloacae were 16% to 65%, 55%, and 35%, respectively. The target values of ertapenem resulting in a bacteriostatic effect and a bactericidal effect were similar to those of benapenem (Table 6).

**TABLE 6:** Percent *fT*_>MIC_ target values of benapenem against the tested strains resulting in the bacteriostatic and bactericidal effects

Bacterial strain	%*fT*_>MIC_^*a*^			
	Bacteriostatic effect		Bactericidal effect	
	Benapenem	Ertapenem	Benapenem	Ertapenem
ATCC 25922 (ESBL^−^ E. coli)	36	80	65 (−1)	
13G136 (ESBL^+^ E. coli)	6	17	22 (−1)	35 (−1)
7742692 (ESBL^+^ E. coli)	7	25	16 (−1)	53 (−2)
ATCC 700603 (ESBL^+^ K. pneumoniae)	18	4		24 (−1)
13C285 (ESBL^+^ K. pneumoniae)	2	9	55 (−1)	38 (−1)
13H279 (Enterobacter cloacae)	17	20	35 (−1)	48 (−2)

a The Δlog(CFU/g) values are given in parentheses. A bacteriostatic effect was a Δlog(CFU/g) of 0, and a bactericidal effect was a Δlog(CFU/g) value of −1 or −2.

In order to ensure a treatment effect, the target values of benapenem that resulted in bacteriostatic and bactericidal effects were determined to be a %*fT*_>MIC_ of 40% and a %*fT*_>MIC_ of 60%, respectively.

### Prediction of clinical efficacy of benapenem against bacterial strains by Monte Carlo simulation.

Figures S13 to S54 show that the probability of target attainment (PTA) of benapenem at a %*fT*_>MIC_ of 0% to 100% against 14 bacterial strains can be more than 90% under all designed dosing regimens. Benapenem showed excellent activity against the *Enterobacteriaceae* bacteria tested.

### Cutoff value determination.

The relationship between PTA and the MIC distribution under different dosing regimens is shown in Fig. S55 to S60. The MIC value at a PTA of 90% was set as the PK/PD cutoff value (12). As shown in Fig. S55 to S57, the MIC values ranged from 0.6 to 3.0 mg/liter at a %*fT*_>MIC_ of 40%, and the MIC values ranged from 0.4 to 2.5 mg/liter at a %*fT*_>MIC_ of 60% under the designed dosing regimens (Fig. S58 to S60). The target values of carbapenems are commonly set equal to 40% to 50% (13). Considering the safety of the clinical use of benapenem, a higher target value is required in patients with a deficient immune system. Therefore, a target value of 60% was used in setting PK/PD cutoff values. From the current dosing guidelines for ertapenem, the intravenous infusion of 1,000 mg of benapenem every 24 h (q24h) (infusion time, 30 min) was determined to be the recommended dosing regimen. As shown in Fig. S60, the MIC value of 1 mg/liter was used the PK/PD cutoff value of benapenem under the conditions described above (PTA = 90%, %*fT*_>MIC_ = 60%, dosage = 1,000 mg q24h with a 30-min infusion).

**FIG 7:**
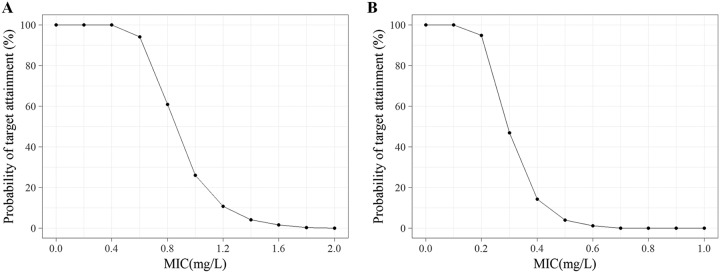
Probability of target attainment (PTA) of ertapenem with various dosing regimens at a %*fT*_>MIC_ of 40% (A) and a %*fT*_>MIC_ of 60% (B) against *Enterobacteriaceae* bacterial strains.

As shown in Fig. 7, for a dosage regimen of 1 g q24h with an infusion time of 0.5 h, the published MIC cutoff values are ≤0.5, 1, and ≥2 μg/ml for interpretive categories of susceptible, intermediate, and resistant, respectively. The observed values were 0.6 μg/ml for susceptible (%*fT*_>MIC_, 40%) and 0.25 μg/ml (%*fT*_>MIC_, 60%) for resistant, and the observed PK/PD cutoff values of ertapenem against *Enterobacteriaceae* under the current recommended dose are 0.6 μg/ml and 0.25 μg/ml at a %*fT*_>MIC_ of 40% and a %*fT*_>MIC_ of 60%, respectively. These results are consistent with the published values of the Clinical and Laboratory Standards Institute (CLSI) and published data (14), which suggest that our results are reliable.

## DISCUSSION

To the best of our knowledge, this is the first study to determine PK/PD cutoff values for benapenem. After investigation of the preclinical PK profiles and antibacterial efficacy of benapenem, a population PK/PD model was developed to describe the concentration-response relationship of benapenem in infected mice. After that, a phase I study of benapenem was conducted. In addition, a population PK model was developed to describe the human PK profiles generated during a benapenem phase I clinical trial. Monte Carlo simulations were performed to predict the clinical effects pf antibacterial treatment associated with several different dosing regimens via the integration of both human PK information and the PK-PD relationship (as shown in the schematic plot) (15–17).

The MIDD approaches integrate knowledge from *in vitro* and *in vivo* PK/PD studies of benapenem, providing a comprehensive understanding of benapenem (e.g., the differences in protein binding between humans and mice). Furthermore, a quantitative comparison of benapenem (a drug candidate) and ertapenem (a drug currently on the market) was performed. The results of all these analyses contribute to scientific decision-making in the development of benapenem, as well as the appropriate use of benapenem in the clinic.

Benapenem exhibited a time-dependent PK profile, whereby %*fT*_>MIC_ could be utilized as a pharmacodynamic index. When %*fT*_>MIC_ was >20%, benapenem exhibited a significant antimicrobial effect; the antimicrobial effect of benapenem tended to be maximum as %*fT*_>MIC_ became >40%. With all the dosing regimens investigated, benapenem exhibited good antibacterial activity against ESBL^−^ and ESBL^+^ Gram-negative bacteria (18).

It is noteworthy that the variability of the target values of benapenem against the different strains tested was significant, which suggests that the variability across the strains may impact the results of *in vivo* antibacterial efficacy studies and lead to potential therapeutic failures in association with resistant strains. The possible reasons for the variability mainly include the different bacterial growth rates and different pathogenicities. The main limitation of this study is the small number of bacterial strains tested. Therefore, the results should be generalized carefully, and further studies with more test strains should be conducted.

Our studies demonstrate that the exposure increment of benapenem is less than the proportional increase in the dose, which was also observed with ertapenem (19, 20). A possible explanation for this is that plasma protein binding may become saturated at high doses. This hypothesis can be validated by the difference in the plasma protein binding rates of benapenem between mice and humans. *In vitro* studies demonstrated that the plasma protein binding of benapenem in mice (dissociation constant, 0.232 μg/ml) was higher than that in humans (dissociation constant, 8.63 μg/ml), which made it easier for the plasma protein binding of benapenem to achieve saturation in mice. It should be noted that the changes in the free fractions of drugs in plasma affect free drug clearance and %*fT*_>MIC_. Therefore, it is necessary to consider the interspecies differences in plasma protein binding for prediction of the volume of distribution and clearance during the scale-up of PK and PD parameters from animal models to humans, as well as during extrapolation of the values for healthy subjects to patients (21).

Clinicians should carefully consider the dosing regimen that is appropriate for use when treating infections with benapenem. This study provides a guide to help determine an appropriate benapenem dosing regimen to provide optimized therapeutic antimicrobial effects. Our results show that the PTA can be improved by prolonging the infusion time from 0.5 h to 3 h for all tested regimens. Several dose optimization strategies may be initiated to maximize the value of PTA, including the use of an increased frequency of dosing and a prolonged infusion time (22, 23). Due to benapenem’s time-dependent-effect properties, shortening the dosing interval and/or prolonging the infusion time may play a more significant role than increasing its dosage in improving the clinical outcome of treatment. Both continuous and extended infusion can provide a higher PTA with a lower total daily dose, but more frequent dosing is often inconvenient, owing to the onerous conditions that it places on nurses and pharmacists (22). Therefore, dosing regimens should be adopted on the basis of the type of infection and the patient’s condition.

In summary, the proposed PK/PD cutoff value provides a thorough understanding of the relationship between the pharmacokinetic profiles of benapenem and its therapeutic effects. Furthermore, the Pop-PK models and plasma protein binding models developed, as well as the model-based simulation developed, can be used to guide the choices for the indications and dosage regimen for benapenem to be used in a phase II clinical trial. Additionally, the PK/PD modeling and simulation may provide a feasible approach to maximizing therapeutic efficacy.

## MATERIALS AND METHODS

### Drugs and reagents.

The benapenem (chemical purity ≥99%), *d*_6_-benapenem (used as the internal standard [IS]; isotopic abundance, 66.9%; chemical purity, ≥99%), and ertapenem (chemical purity, ≥99%) analyzed in this study were provided by XuanZhu Pharma Co., Ltd.

### Instruments.

Liquid chromatography-tandem mass spectrometry (LC-MS/MS) was applied to the detection of the drugs in plasma. The high-performance liquid chromatography system (Shimadzu LC-20AD) consisted of a degasser, a binary pump, and an autosampler. Electrospray ionization mass spectrometry was performed on an API Qtrap 5500 mass spectrometer (Applied Biosystems Inc., USA). *d*_6_-Benapenem was used as the internal standard (IS). The *m/z* for benapenem was 525.1 → 481.1, while that for *d*_6_-benapenem was 531.2 → 487.1. Calibration curves were linear (*r* > 0.99) between 10 and 2,000 ng/ml. The quantitative limit was 10 ng/ml; the intra- and interindividual precisions were <4.85% and <1.47%, respectively; and the intra- and interindividual accuracies were −9.70% and 11.00%, respectively.

### Human PK data and protein binding rate collection.

Clinical phase I PK data for benapenem were obtained from XuanZhu Pharma Co., Ltd. The study participants received an intravenous infusion of benapenem (250 mg, 500 mg, 1,000 mg) over a 30-min infusion period. Blood samples were collected prior to the administration of benapenem (i.e., 0 h) and at 0.25, 0.5, 0.75, 1, 1.5, 2, 3, 5, 8, 12, 24, 36, and 48 h postadministration. PK data for ertapenem were collected from the published literature (19, 20, 24–27) (see Table S1 in the supplemental material). Additional data on the plasma protein binding rates of benapenem and ertapenem were also provided by XuanZhu Pharma Co., Ltd.

### Bacterial strains.

The bacterial strains investigated in experiments evaluating the *in vitro* antibacterial of benapenem (Table 2) included ESBL-producing and ESBL-nonproducing Escherichia coli, ESBL-producing Klebsiella pneumoniae, and Enterobacter cloacae.

### MIC determination.

MICs were determined using the 2-fold agar dilution method described in Clinical and Laboratory Standard Institute (CLSI) guidelines (28). The inocula were cultured on Mueller-Hinton (M-H) agar plates by using a multipoint inoculator, and the inoculum size was 10^4^ CFU/point. E. coli ATCC 25922 was used as the quality control (QC) organism. The validation results indicated that benapenem and ertapenem had MICs for the QC organism ranging from 0.008 to 0.016 mg/liter and 0.004 to 0.008 mg/liter, respectively. The bacteria were treated with benapenem and ertapenem at concentrations ranging from 0.002 to 256 mg/liter.

### Animals.

Beijing Vital Laboratory Animal Technology (Beijing, China) provided ICR mice (males and females; age, 5 to 6 weeks). All animal studies were approved by the Institutional Animal Care and Use Committee of Peking University First Hospital (Beijing, China), and the experiments were conducted according to the guidelines set by the National Research Council (29) (ethics board approval number J201608).

### Infected mouse model establishment and *in vivo* antibacterial activity study.

The antibacterial *in vivo* experiments were carried out with the immunosuppressed mouse thigh infection models. The following strains were selected: ATCC 25922 (ESBL^−^ E. coli), 13G136 (ESBL^+^ E. coli), 7742692 (ESBL^+^ E. coli), ATCC 700603 (ESBL^+^ K. pneumoniae), 13C285 (ESBL^+^ K. pneumoniae), and 13H279 (Enterobacter cloacae).

The mice received intraperitoneal injections of 0.2 ml cyclophosphamide saline solution twice at 3-day intervals to induce neutropenia and, subsequently, immunosuppression. The dosages were 150 mg/kg of body weight and 100 mg/kg, respectively. At 23 h after the last injection of cyclophosphamide, the immunosuppressed mice were infected in the thigh with a log-phase clone in 0.1 ml phosphate-buffered saline by intramuscular injection (30, 31). For the treated groups, the designed dosing regimens of benapenem and ertapenem were administered 2 h after infection to three mice per group. The regimens are listed in Table 7. The blank control group (*n* = 4) received normal saline; two mice in that group were sacrificed 2 h after infection as a preadministration control, and the other two mice used as vehicle controls were sacrificed together with the mice in the treatment groups 24 h later. After 24 h of treatment, the mice were euthanized by cervical displacement, and then the harvested infected thigh muscles were weighed and homogenized. Organ homogenates were plated in serial dilutions for colony counting. The variations in the log(CFU/g) were utilized as the pharmacodynamic index *in vivo*.

**TABLE 7:** MIC and dosing regimens of benapenem and ertapenem for the antibacterial *in vitro* and *in vivo* experiments^*a*^

Bacterial strain	MIC (μg/ml)		Daily dosages (mg/kg)
	Benapenem	Ertapenem	
ATCC 25922 (ESBL^−^ E. coli)	0.016	0.004	0, 1.9, 3.7, 7.3, 14.6, 29.2, 58.4, 116.8
13G136 (ESBL^+^ E. coli)	0.125	0.25	0, 1.9, 3.7, 7.3, 14.6, 29.2, 58.4, 116.8, 233.6
7742692 (ESBL^+^ E. coli)	0.25	0.25	0, 7.3, 14.6, 29.2, 58.4, 116.8, 233.6
ATCC 700603 (ESBL^+^ K. pneumoniae)	0.031	0.031	0, 7.3, 14.6, 29.2, 58.4, 116.8, 233.6
13C285 (ESBL^+^ K. pneumoniae)	0.125	0.062	0, 3.7, 7.3, 14.6, 29.2, 58.4, 116.8, 233.6
13H279 (Enterobacter cloacae)	0.125	0.062	0, 3.7, 7.3, 14.6, 29.2, 58.4, 116.8, 233.6, 467.2

a The dosing interval was q24h, q12h, or q6h.

### Preclinical pharmacokinetic study.

The LC-MS/MS methods were developed by our group to measure the concentrations of benapenem and ertapenem in infected mouse plasma (32). The selected bacterial strain investigated in the preclinical pharmacokinetic study was ATCC 25922 (an ESBL^−^ E. coli strain). In that study, benapenem was injected through the vena caudalis at 1.9, 14.6, 58.4, and 233 mg/kg, and plasma samples were collected at 0, 0.083, 0.25, 0.5, 1, 2, 4, 8, 12, and 24 h. Six mice were assessed at each time point.

### PK/PD modeling.

The model estimations were performed using nonlinear mixed-effect modeling (NONMEM; version 7) software (version VII, level 3; Icon Development Solutions, Ellicott City, MD, USA) by the first-order conditional estimation with interaction method (33). The model-based diagnostic plots were performed by the X-pose visualization method (34). Model validations were based on the NONMEM objective function value (OFV), parameter estimates, relative standard errors (RSE) of the estimates, and exploratory analysis of the goodness-of-fit plots. The ability of the model to describe the observed data was evaluated by a visual predictive check (VPC) of the prediction with 1,000 simulations using the PsN (version 3.4.2) program (35).

### Random effects model.

The random effects of the population pharmacokinetic studies included interindividual random effects and residual random effects. The exponential model (equation 5) was used to describe interindividual variation; the mixed model (equation 6) was used to describe the residual error.

(5) $$P_{i}=P_{\text{pop}}\cdot e^{{\text{η}}_{i}}$$

where *P_i_* is the value of the PK parameter for each individual *i*, *P*_pop_ is the value of the PK parameter for the population, and η*_i_* represents the interindividual variation for individual *i*, which follows a logarithmic normal distribution.

(6) $$C_{\text{obs}}=C_{\text{pred}}\cdot \left(1+{\text{ε}}_{1}\right)+{\text{ε}}_{2}$$

where *C*_obs_ represents the observed concentration, *C*_pred_ represents population predicted concentration, and ε_1_ and ε_2_ represent the additive and proportional residual errors, respectively.

### Fixed-effects model.

Continuous fixed-effect factors, such as the biochemical indicator, were added to PK model in the manner of a power function. The equation is shown below (equation 7):

(7) $$P_{i}=P_{\text{pop}}\cdot {\left(\frac{{\text{COV}}_{i}}{{\text{COV}}_{\mathrm{tv}}}\right)}^{{\text{θ}}_{\text{COV}}}\cdot e^{{\text{η}}_{i}}$$

where θ_COV_ is the influence coefficient of the fixed-effect factors and COV*_tv_* and COV*_i_* represent the population and individual values of the fixed-effect factors, respectively.

Discontinuous fixed-effect factors, such as sex, were added to PK model in the manner of a condition. The equation is shown below (equation 8):

(8) $$P_{i}=P_{\text{pop}}\cdot {\text{θ}}_{\text{SEX}}{}^{\text{SEX}}\cdot e^{{\text{η}}_{i}}$$

where θ_SEX_ represents the influence coefficient of sex.

### Protein binding model.

The PK model was based on total plasma drug concentration data; note, however, that only free drugs exert antimicrobial activity. Therefore, the protein binding model was developed for the PK/PD correlation analysis (i.e., prior to PK model development). In this study, a two-site binding model was used to fit the protein binding data (36).

(9) $$C_{b}=\frac{B_{\max\;}\cdot C_{u}}{C_{50}+C_{u}}+\text{slope}\cdot C_{u}$$

where *C_u_* and *C_b_* represent the free drug concentration and the binding drug concentration, respectively. *B*_max_ is the maximum load in the binding sites, and *C*_50_ is the apparent dissociation constant, namely, the free drug concentration reaching half of the maximum load. Both *B*_max_ and *C*_50_ are the parameters corresponding to the binding sites with a high affinity. Slope is the ratio of *B*_max_ and *C*_50_ which correspond to the binding sites with a low affinity.

### Calculation of free drug concentration.

The relationship between the total drug concentration (*C_t_*) and the free drug concentration (*C_u_*) can be described by the following equation:

(10) $$C_{t}=C_{b}+C_{u}=\frac{B_{\max\;}\cdot C_{u}}{C_{50}+C_{u}}+\text{slope}\cdot C_{u}+C_{u}$$

Equation 10 can be rearranged into equation 11.

(11) $$a\cdot C_{u}^{2}+b\cdot C_{u}+c=0$$

In equation 11, the parameters *a*, *b*, and *c* are defined as follows: *a* is equal to the slope + 1, *b* is equal to (slope + 1)·*C*_50_ + *B*_max_ – *C_t_*, and *c* is equal to −*C_t_*·*C*_50_.

The free drug concentration can be described by the following equations.

(12) $$C_{u}=\frac{-b+\sqrt{b^{2}-4ac}}{2a}$$

### PK/PD correlation analysis.

Correlation analyses between the PK/PD indexes %*fT*_>MIC_, *fC*_max_/MIC, and *f*AUC/MIC and the variations of log(CFU/g) under different dosing regimens were performed. The correlation coefficients were applied to evaluate the mode of action of benapenem (i.e., whether it is in a time-dependent manner or a concentration-dependent manner).

### Calculation of %*fT*_>MIC_.

The following equation was used to calculate %*fT*_>MIC_:

(13) $$\%fT_{\text{>MIC}}=\frac{{\sum_{i=1}^{n}}_{}^{}f\left(C_{u}^{i}\right)}{n}\cdot 100\%$$

where *n* is the total sampling number, *i* is the sampling point, and is the free drug concentration of each sampling point. The following logical equation was used to judge whether the free drug concentration was greater than the MIC. If the return value is 1, the free drug concentration (*x*) is greater than the MIC, and if the return value is 0, the free drug concentration is less than or equal to the MIC.

$$f\;\left(x\right)=\{\begin{array}{c} 1,x\;>\text{ MIC}\\ 0,x\;\leq \text{ MIC} \end{array}$$

### Data analysis and model simulations.

The antibacterial effect of benapenem was performed using a Monte Carlo simulation. MIC values were generated from the discrete MIC distribution obtained for the isolates. The regimens simulated were 250 mg, 500 mg, and 1,000 mg every 6, 8, 12, or 24 h (q6h, q8h, q12h, or q24h, respectively) with a 0.5-, 0.75-, 1-, 2-, and 3-h infusion. The probability of target attainment (PTA) was calculated based on the simulated plasma concentrations at 0.1-min intervals by using a two-compartment model. The PTA at various %*fT*_>MIC_ values (range, 0% to 100% at 5% intervals) for the dosage interval was calculated by using each designed dosing regimen against the different bacterial strains.

PTA (in percent) was calculated by using equation 14:

(14) $$\text{PTA}=\frac{\sum_{i=1}^{n}f(T_{\text{>MIC}}{\%}_{i})\;}{n}\cdot 100\%$$

where *n* is the total number of subjects, *i* denotes an individual, and *T*_>MIC_%*_i_* is the corresponding %*T*_>MIC_ for each individual. The following logical equation was used to judge whether *T*_>MIC_ is greater than the target value. If the return value is 1, the %*T*_>MIC_ (*x*) is greater than or equal to the target value, and if the return value is 0, the %*T*_>MIC_ is less than target value.

$$f\;\left(x\right)=\{\begin{array}{c} 1,x\geq \text{target}\\ 0,x<\text{target} \end{array}$$

## Supplementary Material

Supplemental file 1
