# Supplementary material for: Model-Informed Drug Development, Pharmacokinetic/Pharmacodynamic Cutoff Value Determination, and Antibacterial Efficacy of Benapenem against Enterobacteriaceae
Source: Antimicrob Agents Chemother. 2020 Feb 21;64(3):e01751-19. doi: 10.1128/AAC.01751-19 (PMC7038265; doi:10.1128/AAC.01751-19)
Supplement: Supplemental file 1 [file AAC.01751-19-s0001.pdf]

Table S1. Demographic background information of the ertapenem PK data

Literature No.	Mean				
	Subject Number	Age (year)	Height (cm)	Weight (kg)	BMI (kg/m^2)
1	10 (F=5, M=5)	34.1 (8.4)	171.9 (8.5)	66.7 (9.6)	22.5 (1.9)
2	16 (F=8, M=8)	32.8 (6.0)	-	69.6 (13.2)	-
3	10 (F=5, M=5)	35 (5)	173 (9)	70 (13)	-
4	6 (F=3, M=3)	22~37	160 (8.8)	64.7 (8.6)	25.3 (2.4)
5	26 (F=20, M=6)	30 (6)		74 (12)	
6	16 (F=8, M=8)				
7	-	-	-	-	-
8	12	27	-	-	-

Table S2. Protein binding rates of different concentrations of benapenem and ertapenem in human and mice plasma

Concentration (μM)	Berapenem		Ertapenem	
	human plasma (%)	mice plasma (%)	human plasma (%)	mice plasma (%)
0.2	92.1 ± 0.4	>95.6	93.1 ± 0.2	87.9 ± 0.6
2	93.5 ± 0.4	>99.6	94.3 ± 0.3	87.6 ± 0.5
20	93.9 ± 0.5	99.8 ± 0.0	95.6 ± 0.4	88.1 ± 0.6
100	84.3 ± 2.1	99.7 ± 0.0	90.6 ± 0.1	79.2 ± 1.5
200	82.5 ± 1.5	99.3 ± 0.0	87.1 ± 0.8	71.7 ± 1.3
500	63.9 ± 1.0	60.7 ± 4.1	73.0 ± 3.1	54.3 ± 3.2
1000	50.5 ± 2.2	32.6 ± 5.9	50.4 ± 2.6	38.0 ± 5.5
2 (warfarin)	99.7 ± 0.1	97.4 ± 0.4	99.5 ± 0.1	97.8 ± 0.1

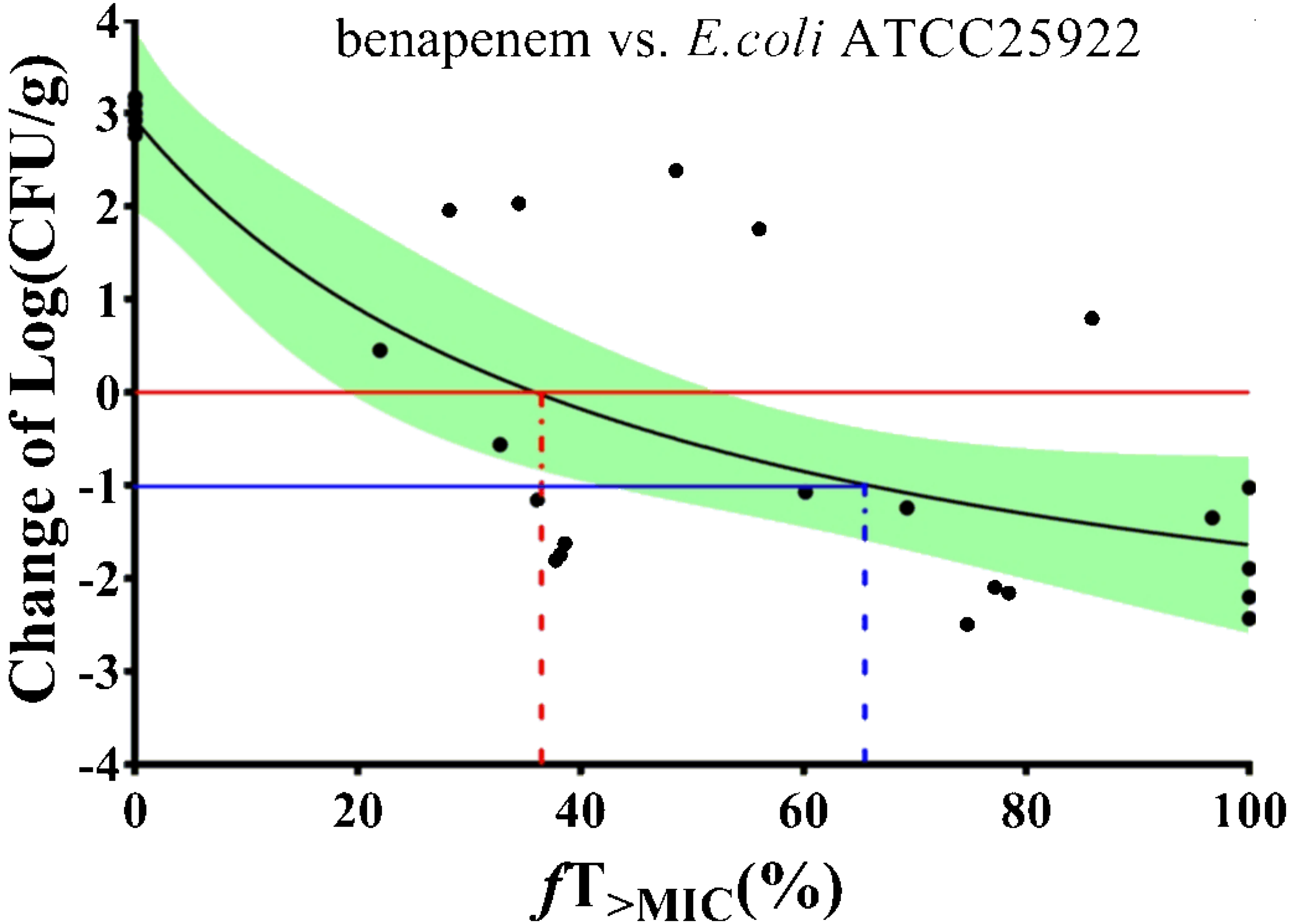

Figure S1. The correlation analysis between %fT $_{>MIC}$ and the *in vivo* anti-bacteria effects [log(CFU/g)] of benapenem against ATCC25922(ESBL-*E.coli*). The curve represents the trend line; the green area represents 95% confidence interval; the solid dots represent the observed data. The values of %fT $_{>MIC}$ at △log(CFU/g)=0 and △log(CFU/g)=-1 ware are marked.

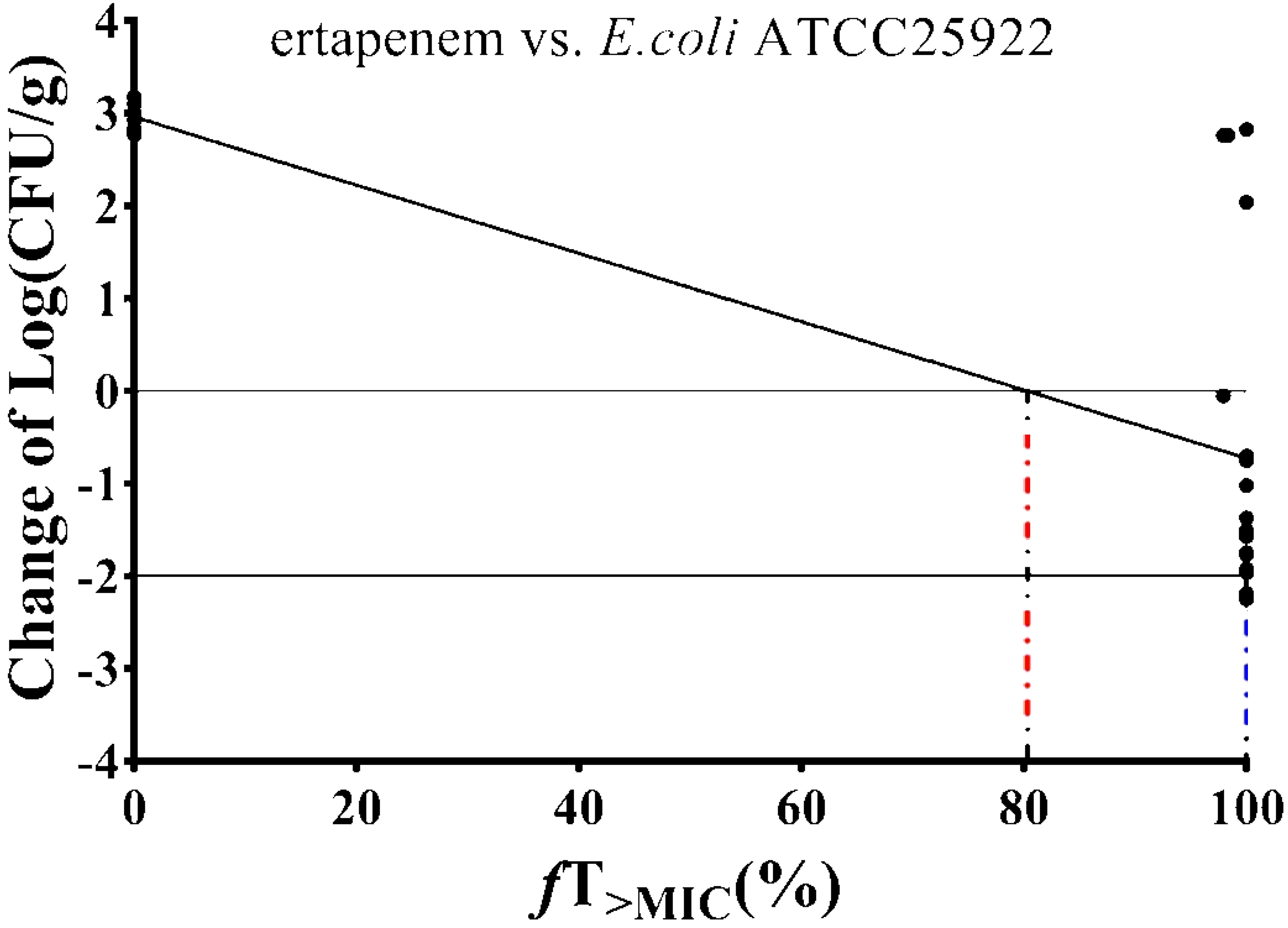

Figure S2. The correlation analysis between $\%fT_{>MIC}$ and the *in vivo* anti-bacteria effects [log(CFU/g)] of ertapenem against ATCC25922(ESBL-*E.coli*). The curve represents the trend line; the solid dots represent the observed data. The values of $\%fT_{>MIC}$ at △log(CFU/g)=0 and △log(CFU/g)= -2 ware are marked.

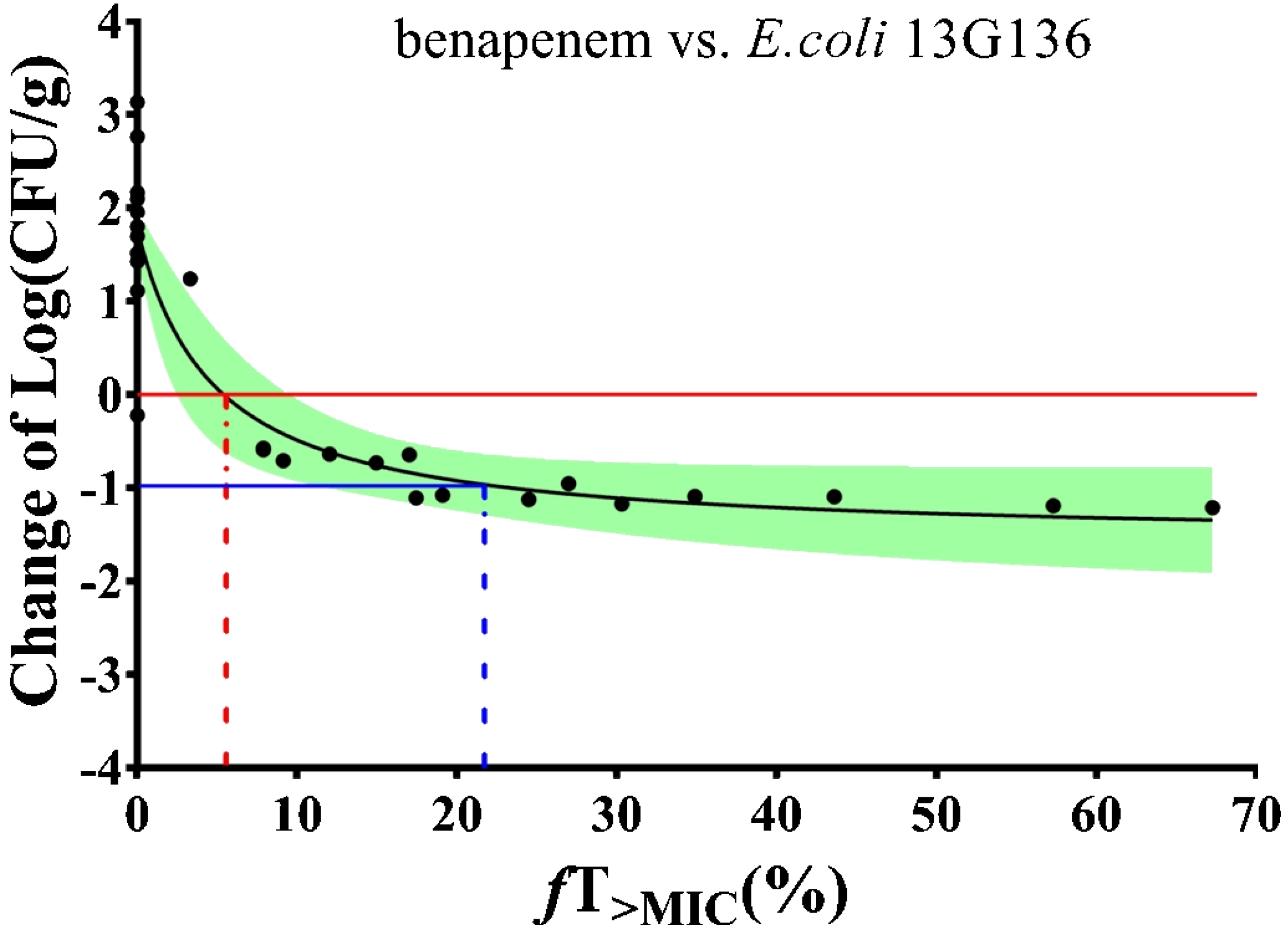

Figure S3. The correlation analysis between %$fT_{>MIC}$ and the *in vivo* anti-bacteria effects [log(CFU/g)] of benapenem against 13G136(ESBL+*E.coli*). The curve represents the trend line; the green area represents 95% confidence interval; the solid dots represent the observed data. The values of %$fT_{>MIC}$ at △log(CFU/g)=0 and △log(CFU/g)=-1 ware are marked.

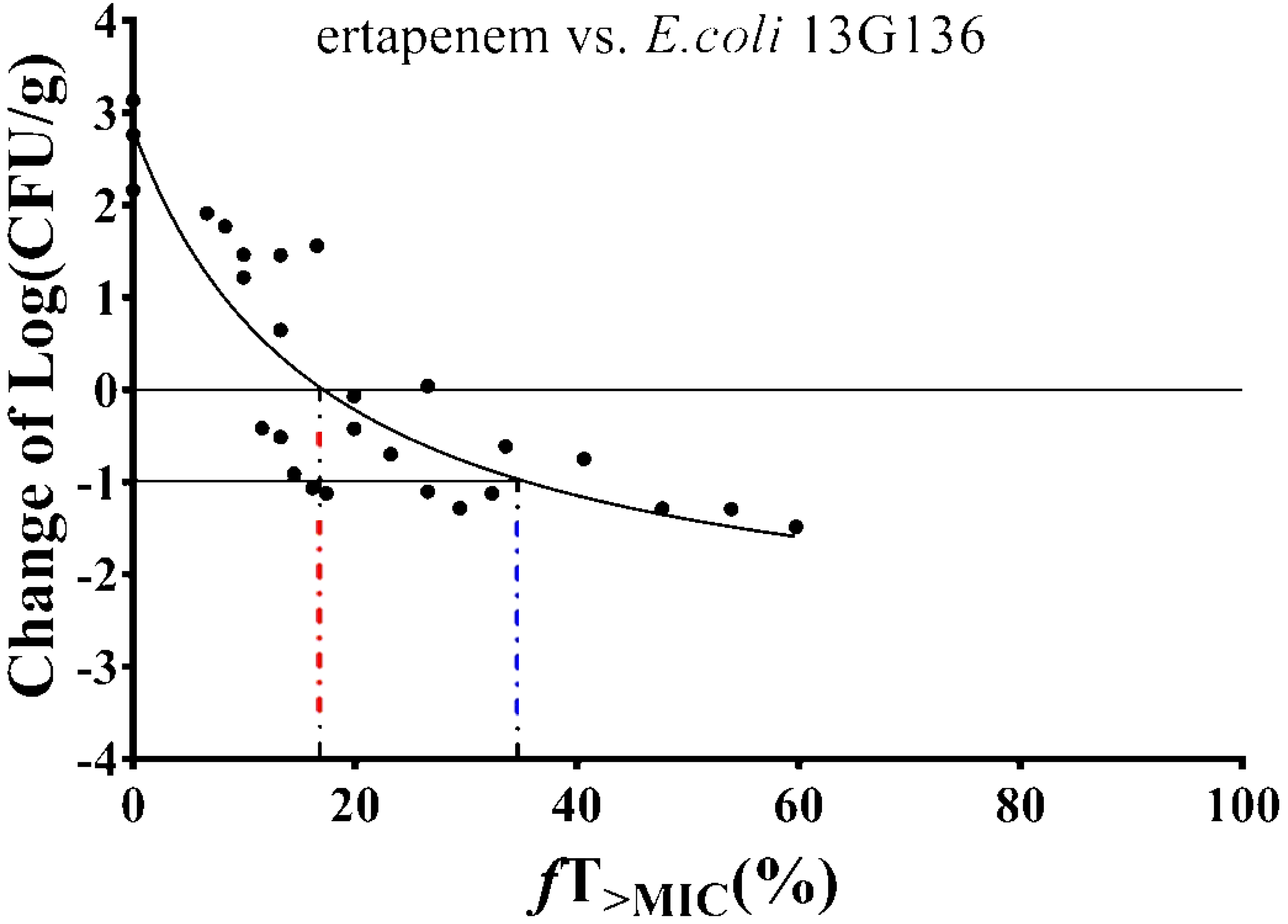

Figure S4. The correlation analysis between $\% fT_{>MIC}$ and the *in vivo* anti-bacteria effects [log(CFU/g)] of ertapenem against 13G136(ESBL+*E.coli*). The curve represents the trend line; the solid dots represent the observed data. The values of $\% fT_{>MIC}$ at △log(CFU/g)=0 and △log(CFU/g)=-1 ware are marked.

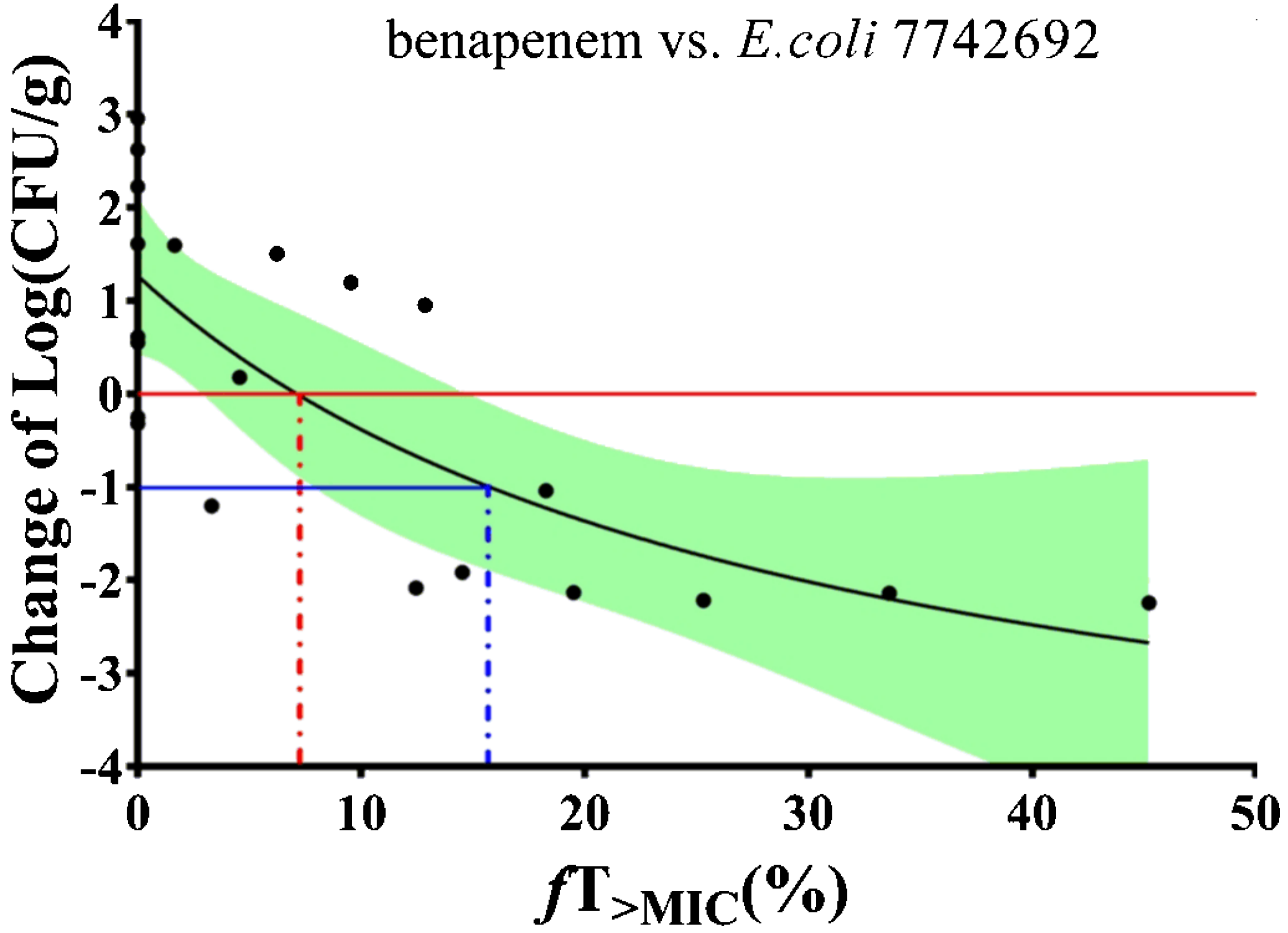

Figure S5. The correlation analysis between %$fT_{>MIC}$ and the *in vivo* anti-bacteria effects [log(CFU/g)] of benapenem against 7742692(ESBL+*E.coli*). The curve represents the trend line; the green area represents 95% confidence interval; the solid dots represent the observed data. The values of %$fT_{>MIC}$ at △log(CFU/g)=0 and △log(CFU/g)=-1 ware are marked.

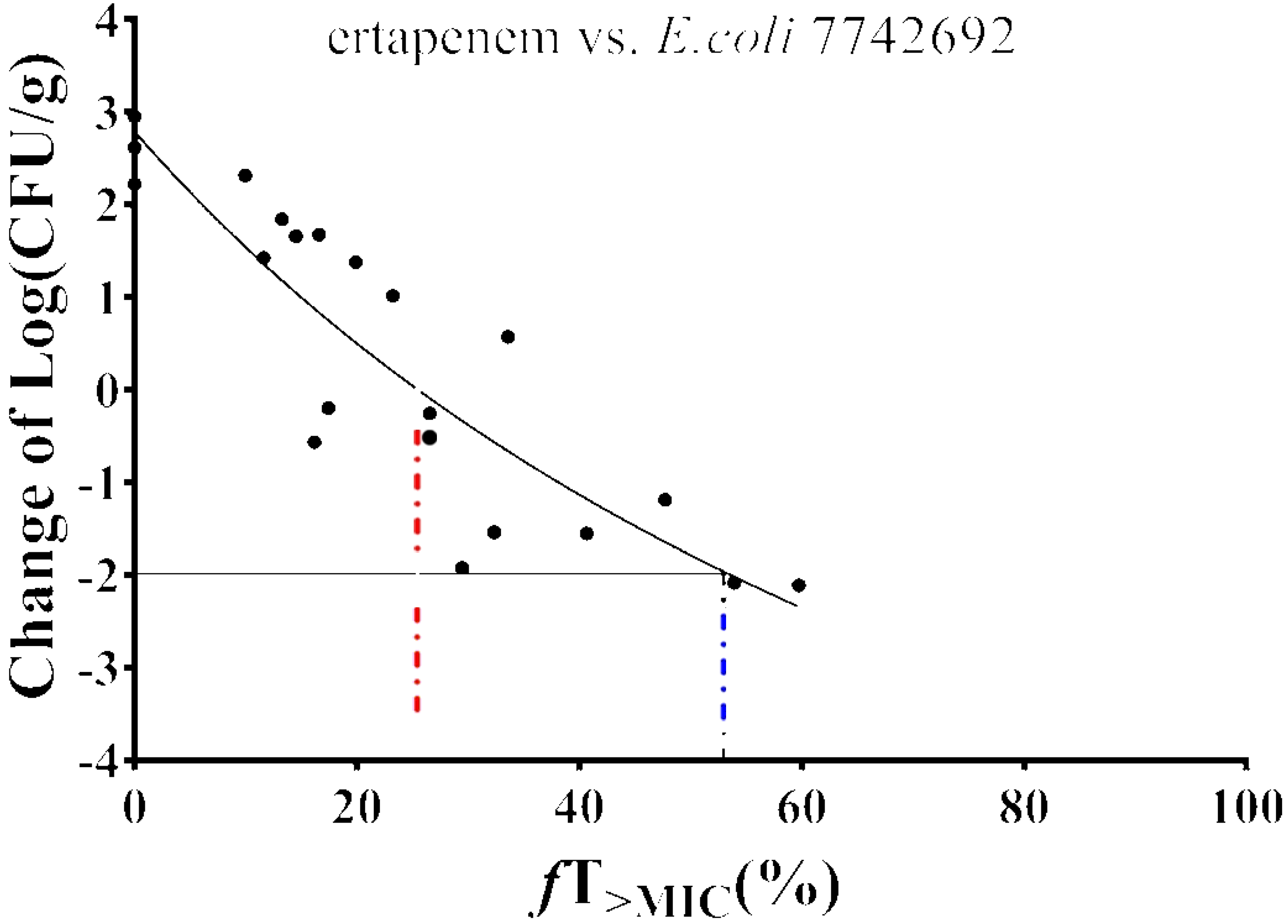

Figure S6. The correlation analysis between %fT$_{>MIC}$ and the *in vivo* anti-bacteria effects [log(CFU/g)] of ertapenem against 7742692(ESBL+*E.coli*). The curve represents the trend line; the solid dots represent the observed data. The values of %fT$_{>MIC}$ at △log(CFU/g)=0 and △log(CFU/g)= -2 ware are marked.

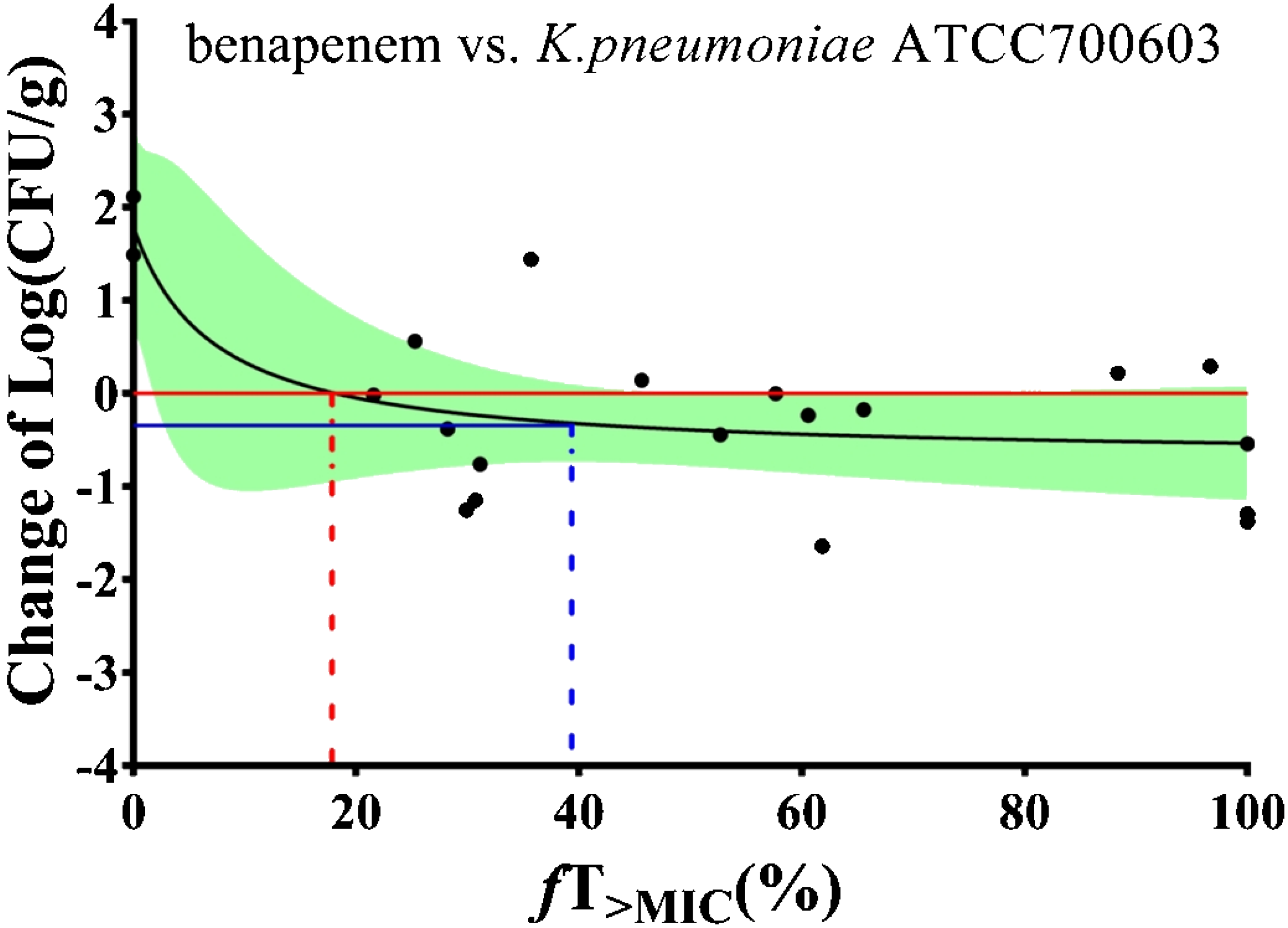

Figure S7. The correlation analysis between %fT$_{>MIC}$ and the *in vivo* anti-bacteria effects [log(CFU/g)] of benapenem against ATCC700603 (ESBL+*K.pneumoniae*). The curve represents the trend line; the green area represents 95% confidence interval; the solid dots represent the observed data. The values of %fT$_{>MIC}$ at △log(CFU/g)=0 and the curve inflection point ware are marked.

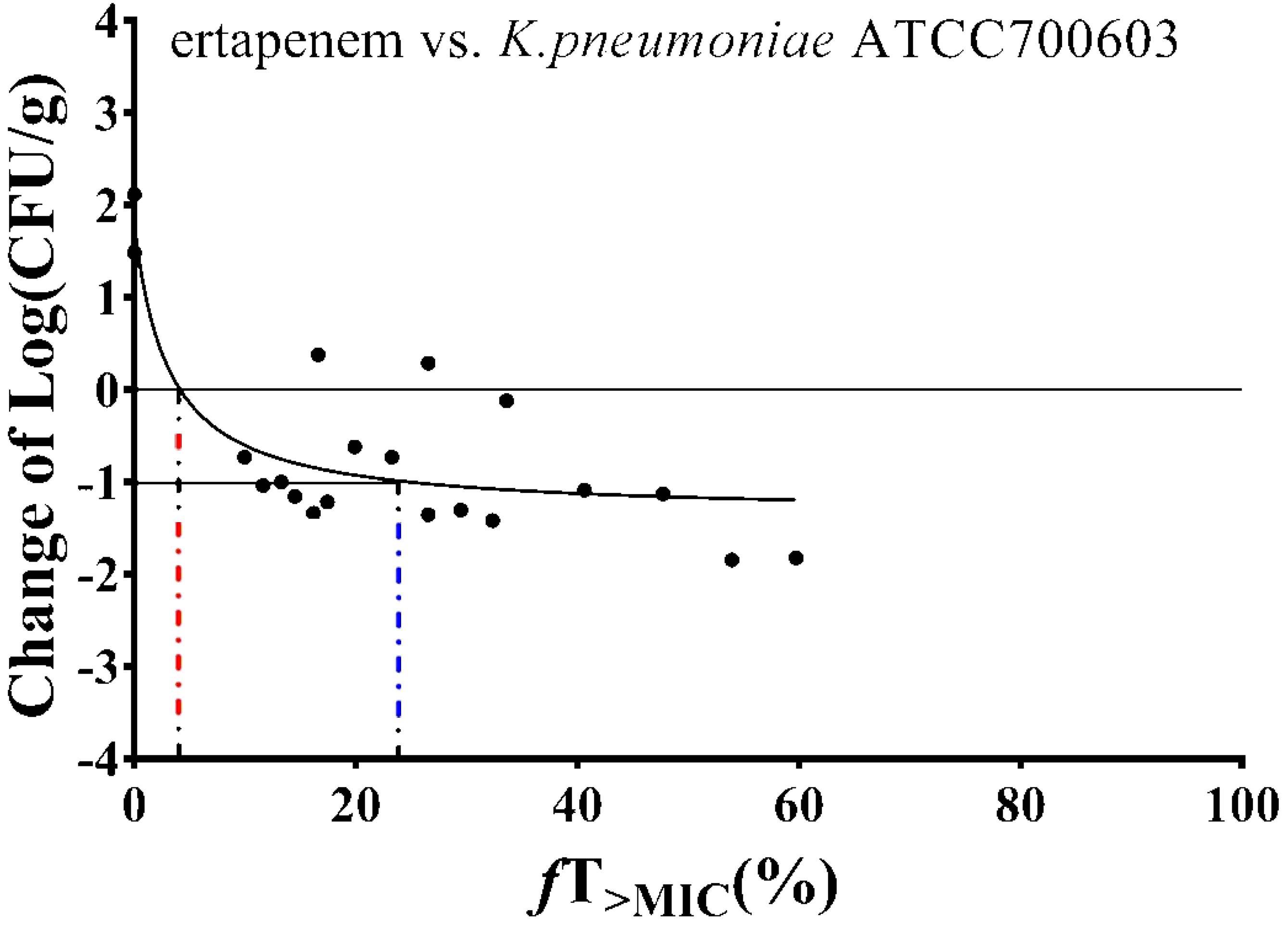

Figure S8. The correlation analysis between %$fT_{>MIC}$ and the *in vivo* anti-bacteria effects [log(CFU/g)] of ertapenem against ATCC700603 (ESBL+*K.pneumoniae*). The curve represents the trend line; the solid dots represent the observed data. The values of %$fT_{>MIC}$ at △log(CFU/g)=0 and △log(CFU/g)=-1 ware are marked.

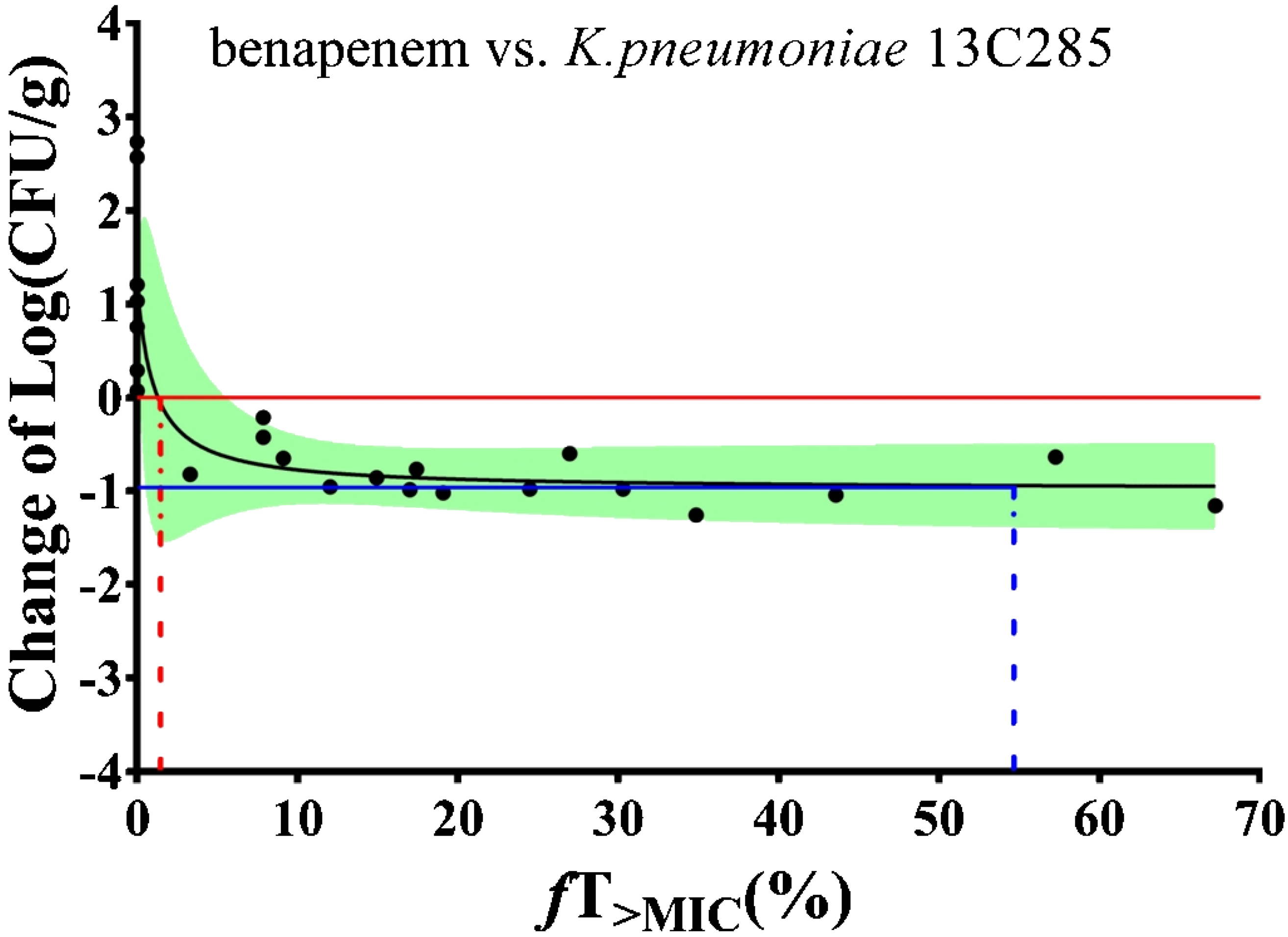

Figure S9. The correlation analysis between %$fT_{>MIC}$ and the *in vivo* anti-bacteria effects [log(CFU/g)] of benapenem against 13C285 (ESBL+*K.pneumoniae*). The curve represents the trend line; the green area represents 95% confidence interval; the solid dots represent the observed data. The values of %$fT_{>MIC}$ at △log(CFU/g)=0 and the curve inflection point ware are marked.

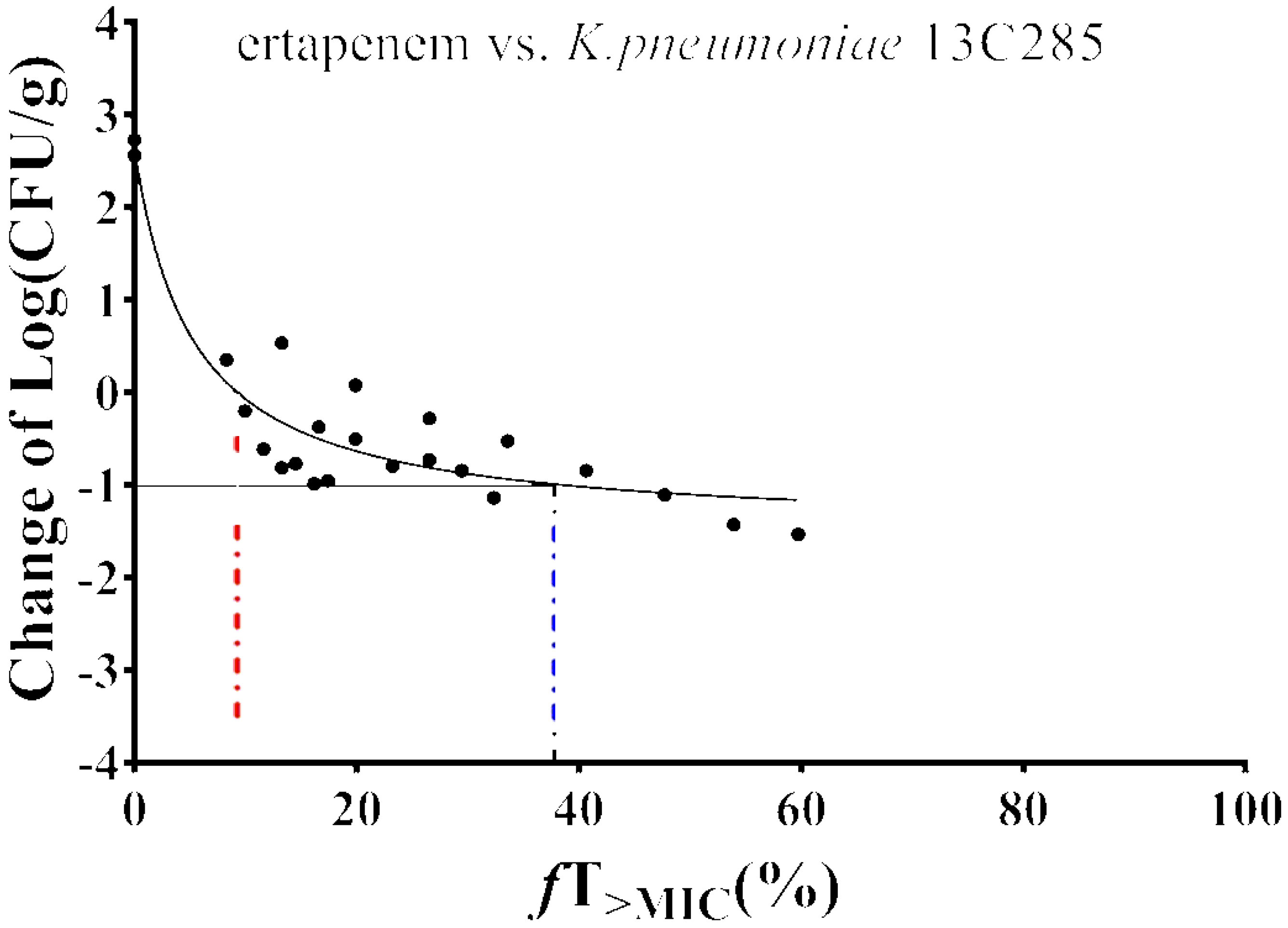

Figure S10. The correlation analysis between %$fT_{>MIC}$ and the *in vivo* anti-bacteria effects [log(CFU/g)] of ertapenem against 13C285 (ESBL+*K.pneumoniae*). The curve represents the trend line; the solid dots represent the observed data. The values of %$fT_{>MIC}$ at △log(CFU/g)=0 and △log(CFU/g)=-1 ware are marked.

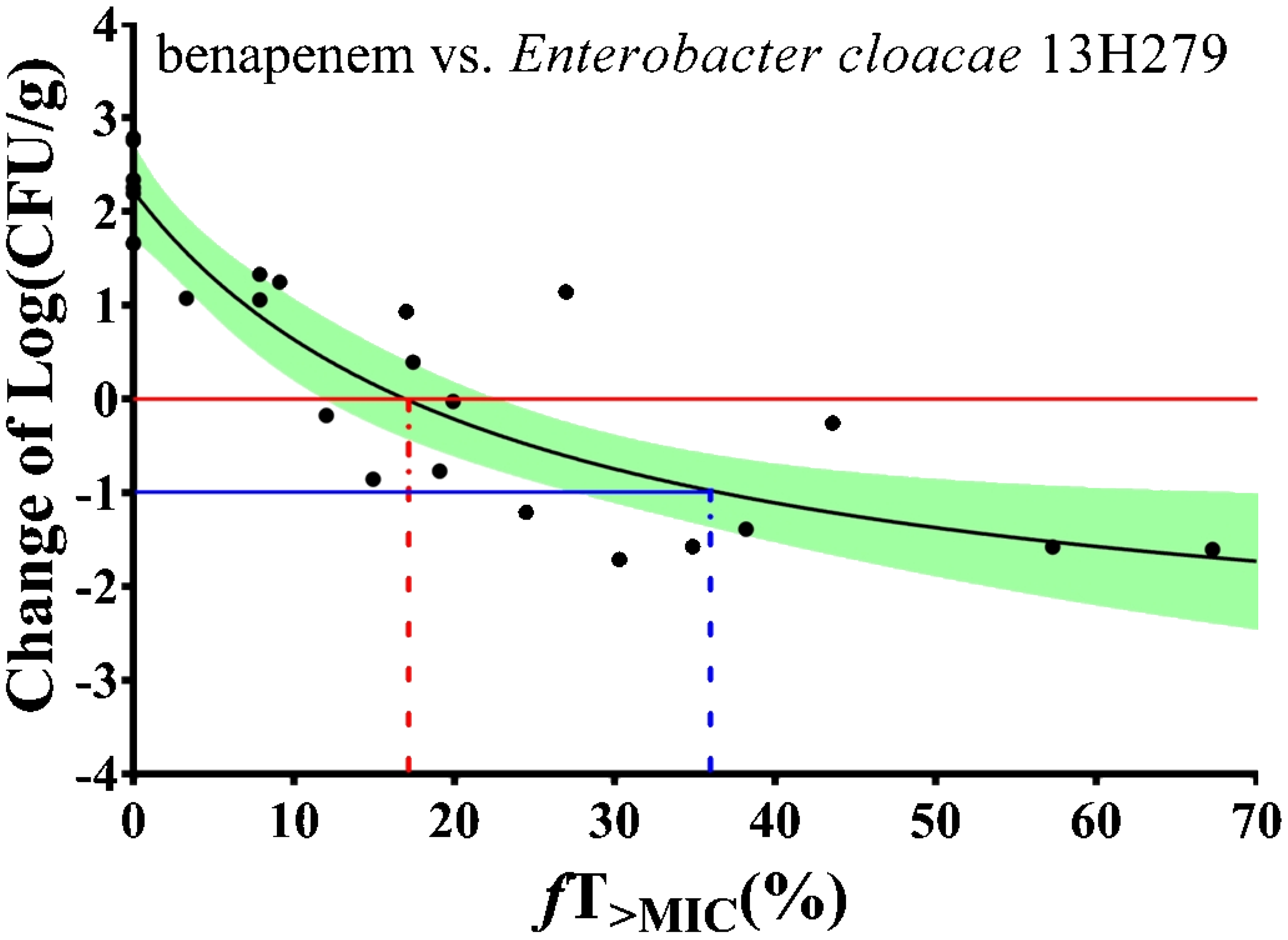

Figure S11. The correlation analysis between %$fT_{>MIC}$ and the *in vivo* anti-bacteria effects [log(CFU/g)] of benapenem against 13H279 (*Enterobacter cloacae*). The curve represents the trend line; the green area represents 95% confidence interval; the solid dots represent the observed data. The values of %$fT_{>MIC}$ at △log(CFU/g)=0 and △log(CFU/g)=-1 ware are marked.

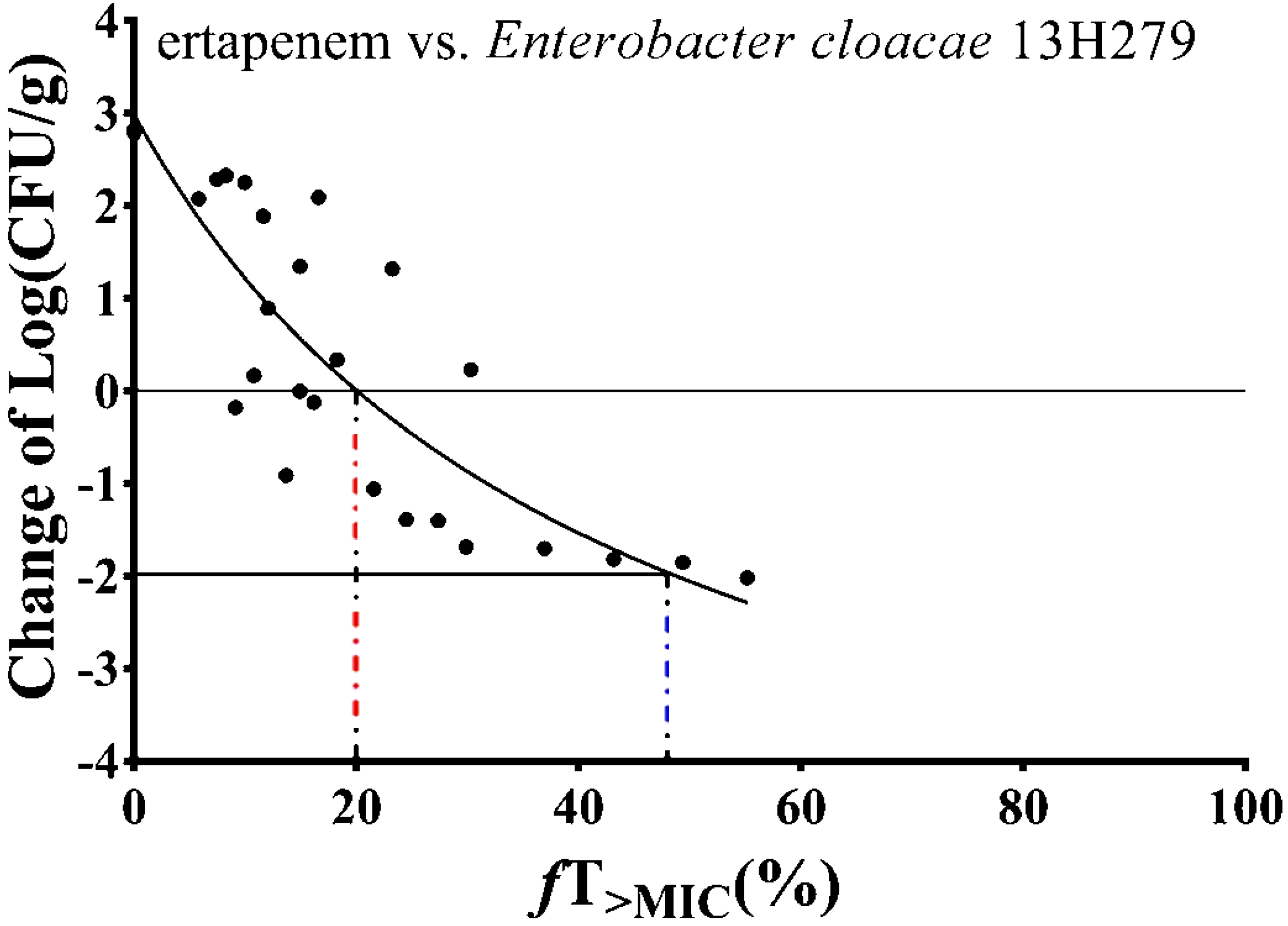

Figure S12. The correlation analysis between %$fT_{>MIC}$ and the *in vivo* anti-bacteria effects [log(CFU/g)] of ertapenem against 13H279 (*Enterobacter cloacae*). The curve represents the trend line; the solid dots represent the observed data. The values of %$fT_{>MIC}$ at △log(CFU/g)=0 and △log(CFU/g)= -2 ware are marked.

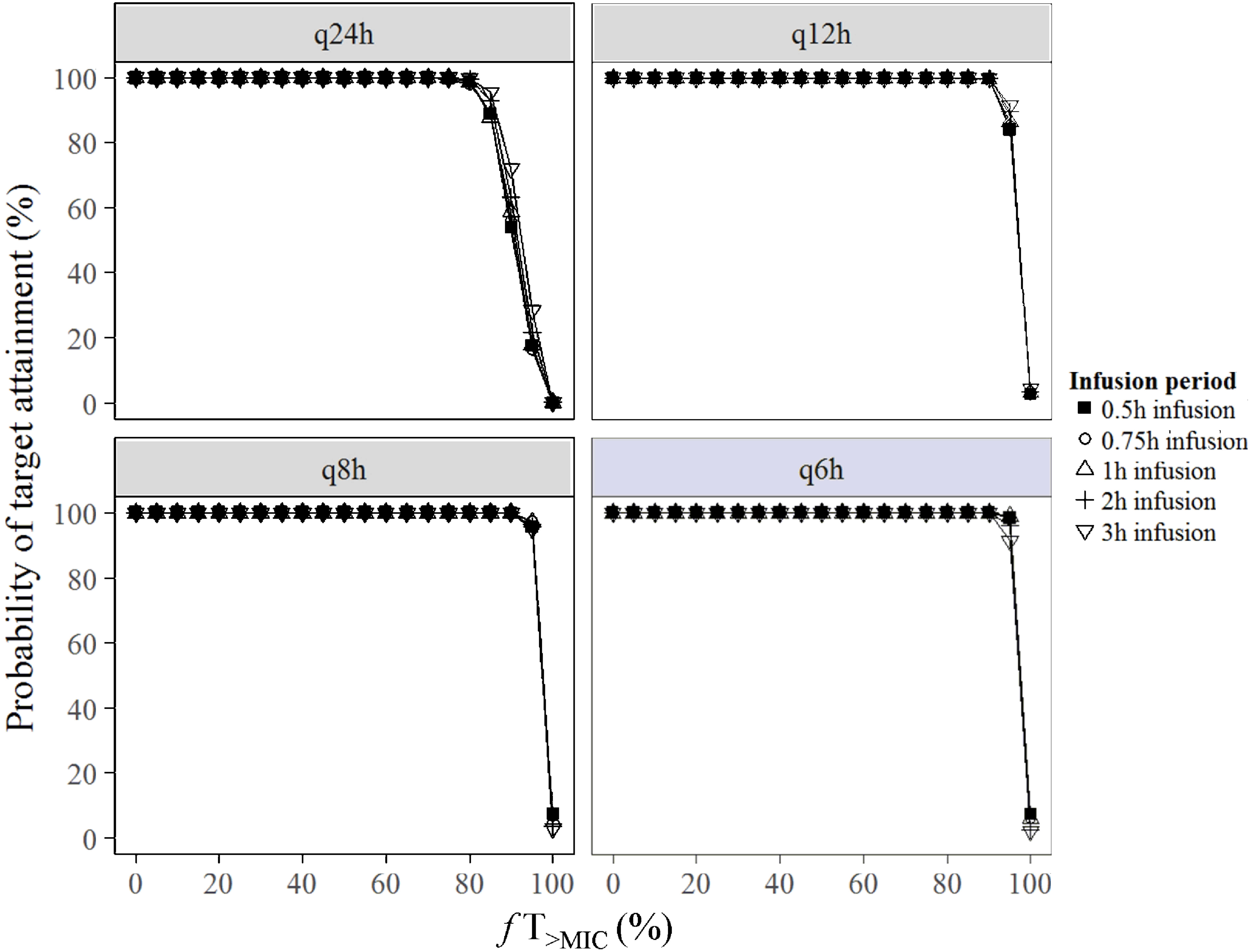

Figure S13. Probability of target attainment (PTA) of benapenem at %$fT_{>MIC}$ of 0% to 100% against *Citrobacter* under dose of 250 mg with different infusion time and dose interval.

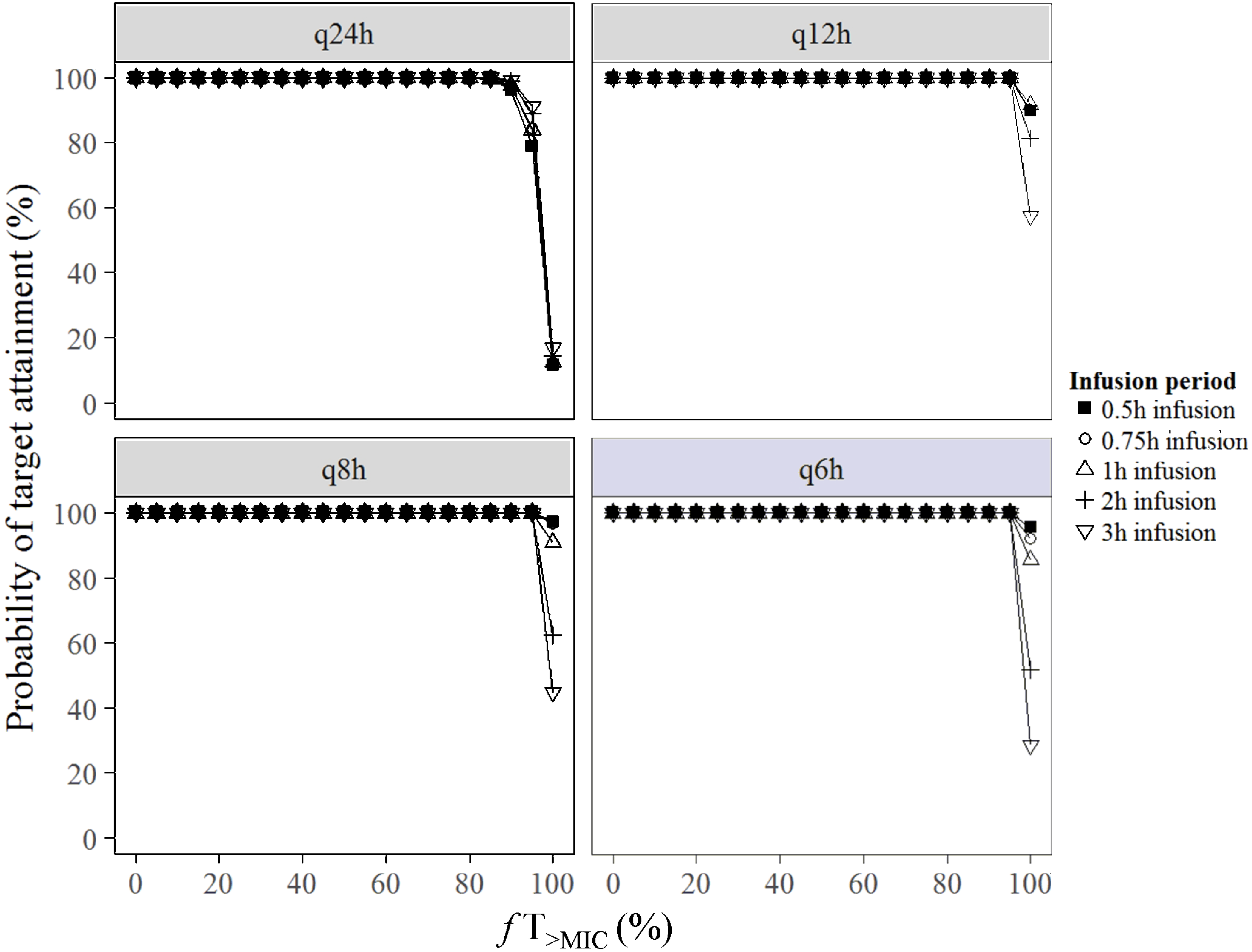

Figure S14. Probability of target attainment (PTA) of benapenem at %$f\mathrm{T}_{>\mathrm{MIC}}$ of 0% to 100% against *Citrobacter* under dose of 500 mg with different infusion time and dose interval.

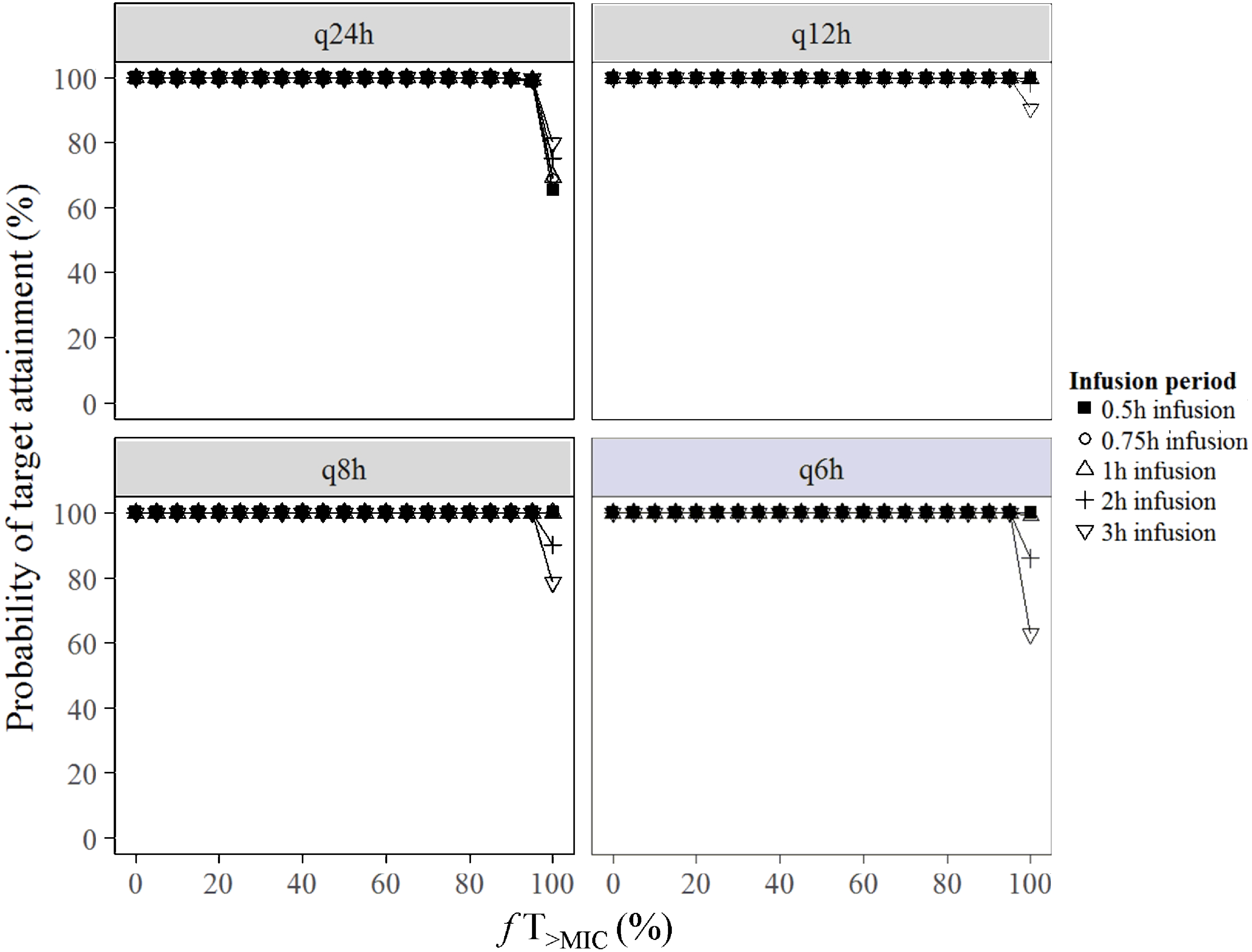

Figure S15. Probability of target attainment (PTA) of benapenem at $\%fT_{>MIC}$ of 0% to 100% against *Citrobacter* under dose of 1000 mg with different infusion time and dose interval.

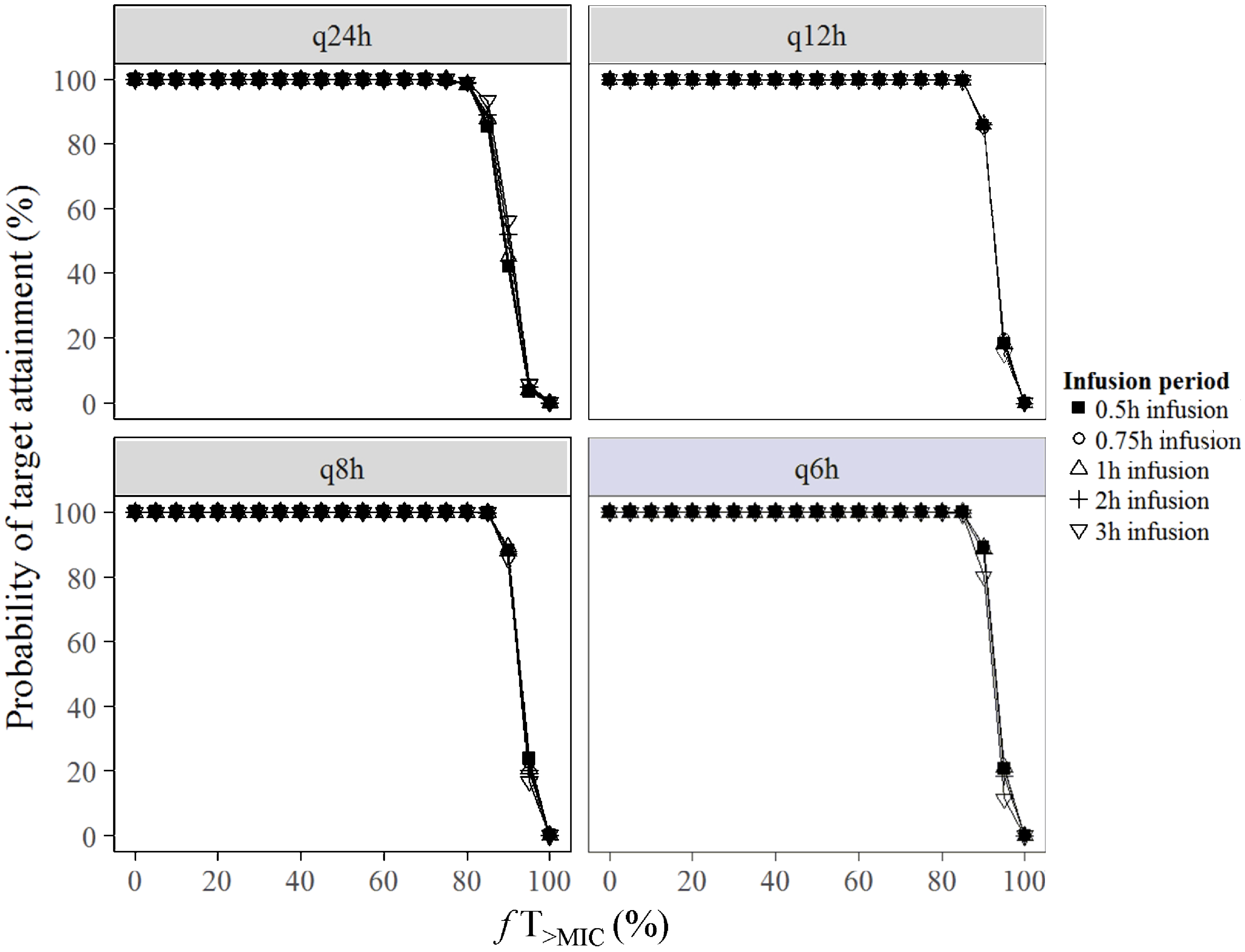

Figure S16. Probability of target attainment (PTA) of benapenem at %$fT_{>MIC}$ of 0% to 100% against *Enterobacter aerogenes* under dose of 250 mg with different infusion time and dose interval.

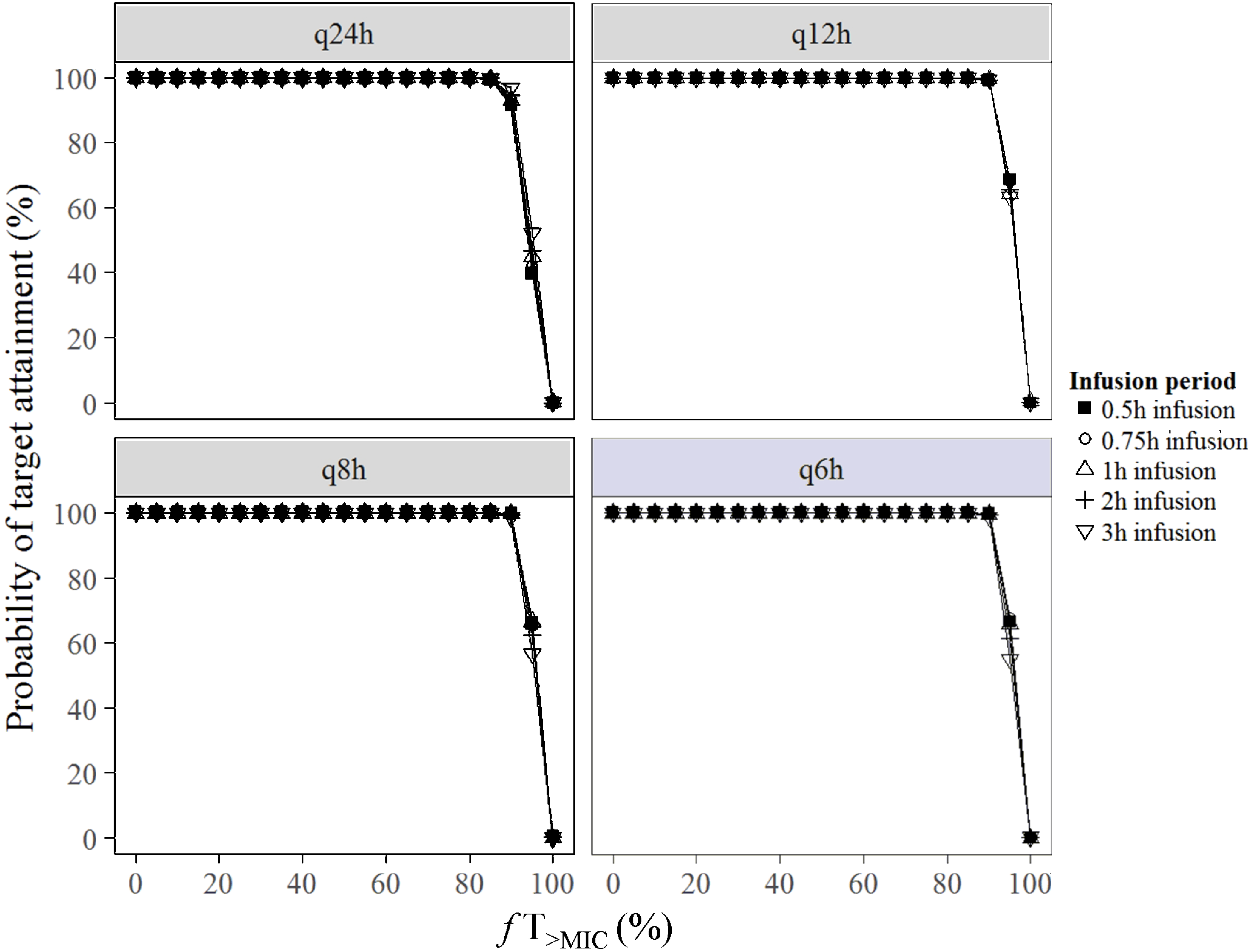

Figure S17. Probability of target attainment (PTA) of benapenem at %$fT_{>MIC}$ of 0% to 100% against *Enterobacter aerogenes* under dose of 500 mg with different infusion time and dose interval.

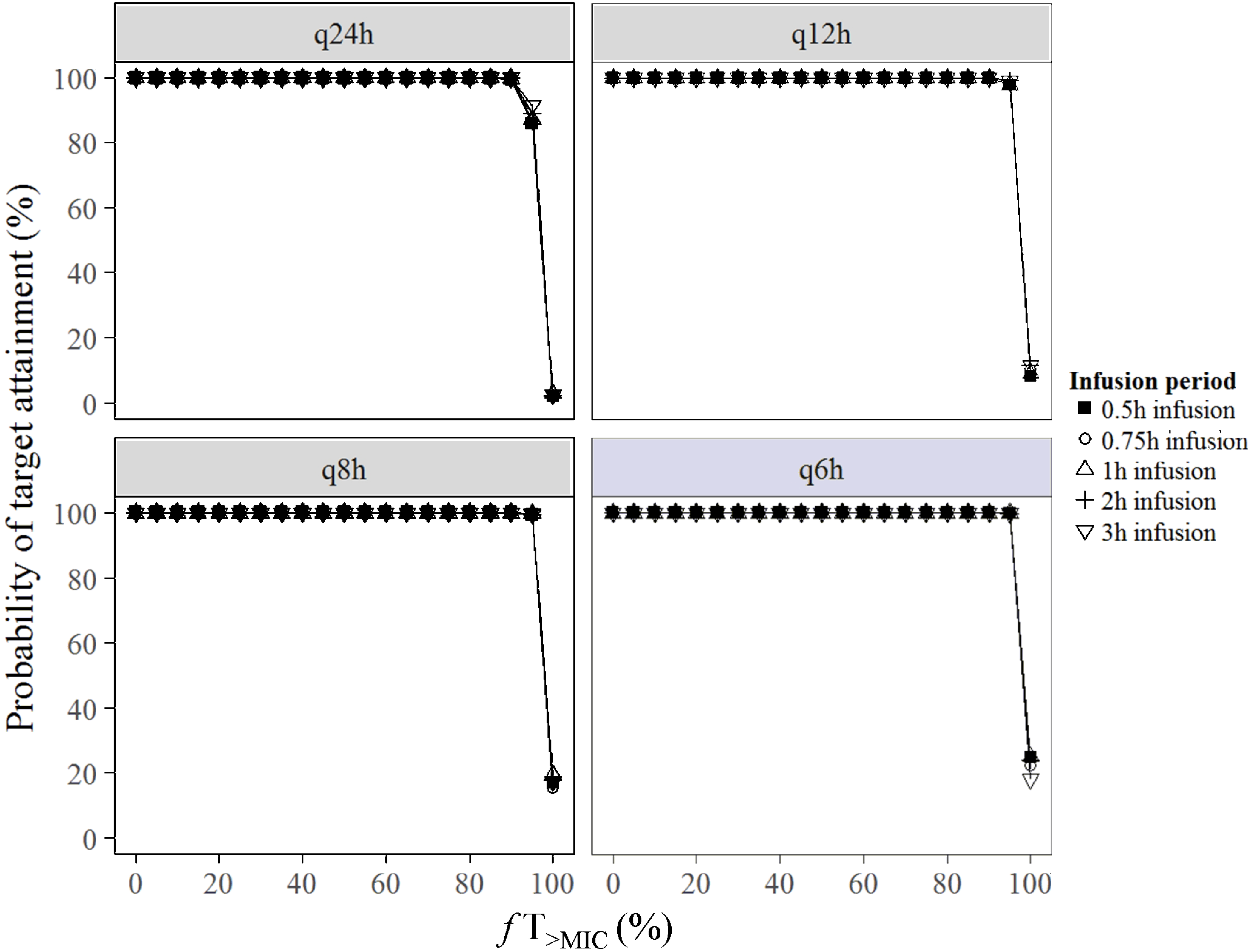

Figure S18. Probability of target attainment (PTA) of benapenem at %$fT_{>MIC}$ of 0% to 100% against *Enterobacter aerogenes* under dose of 1000 mg with different infusion time and dose interval.

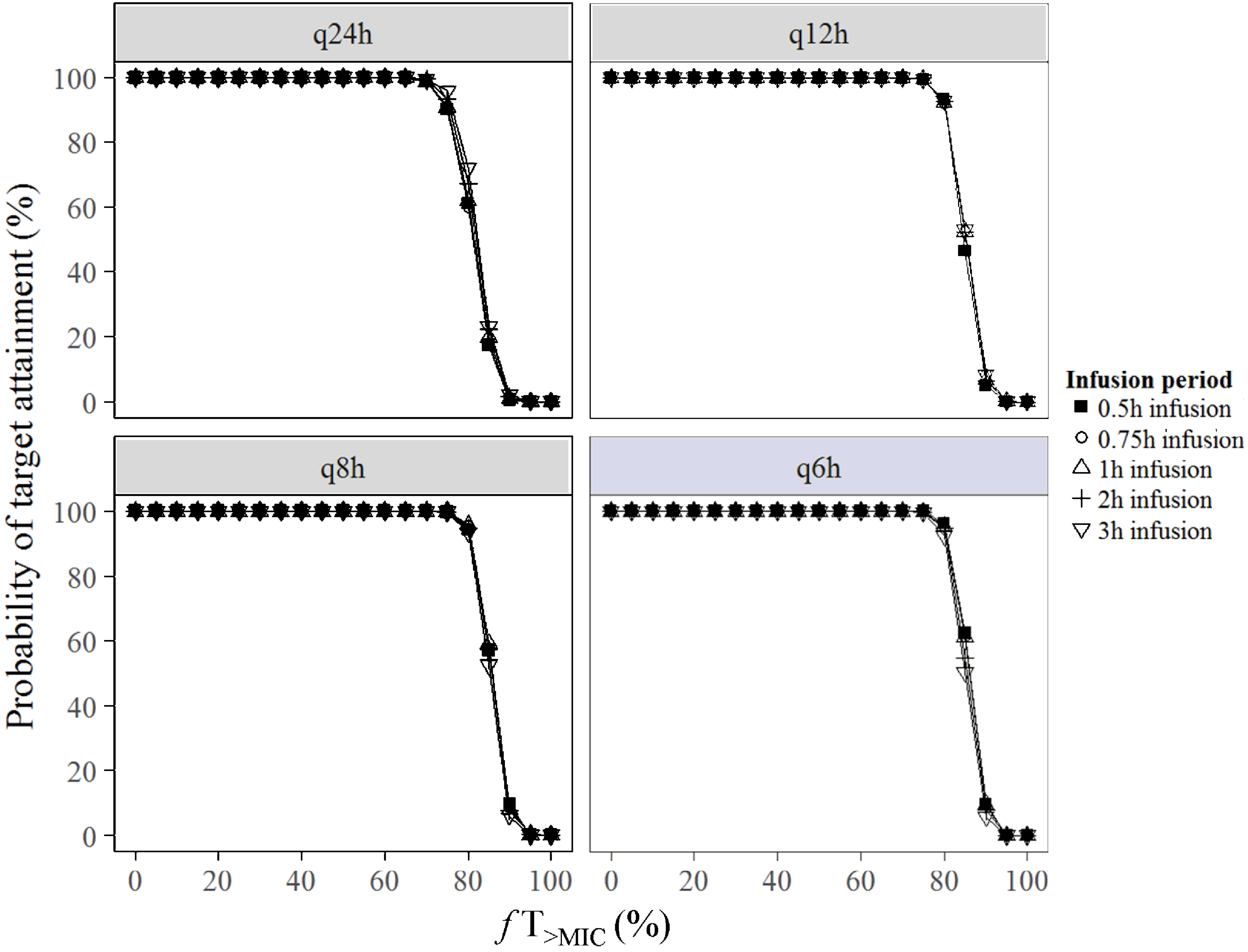

Figure S19. Probability of target attainment (PTA) of benapenem at %$fT_{>MIC}$ of 0% to 100% against *Enterobacter cloacae* under dose of 250 mg with different infusion time and dose interval.

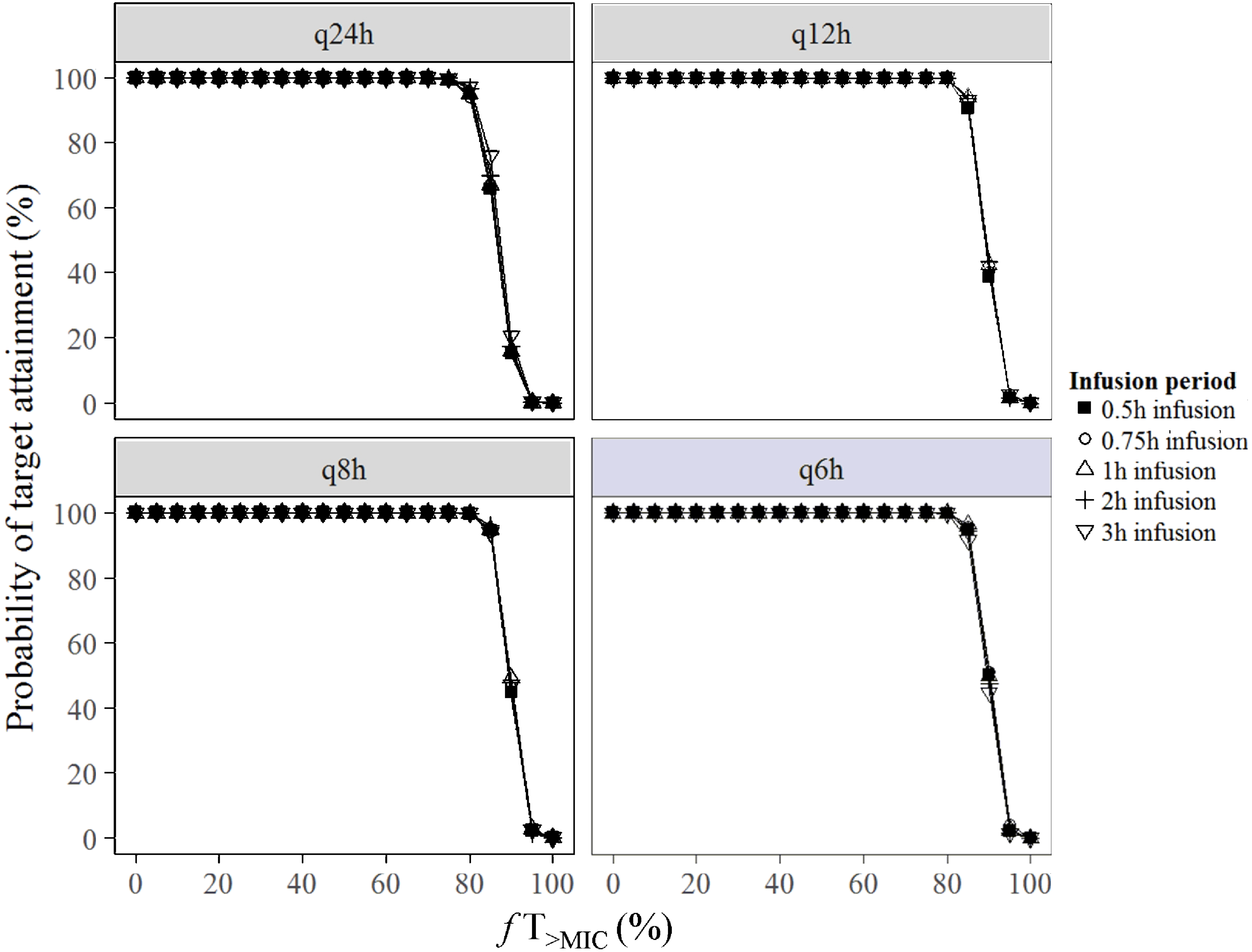

Figure S20. Probability of target attainment (PTA) of benapenem at %$f\mathrm{T}_{>\mathrm{MIC}}$ of 0% to 100% against *Enterobacter cloacae* under dose of 500 mg with different infusion time and dose interval.

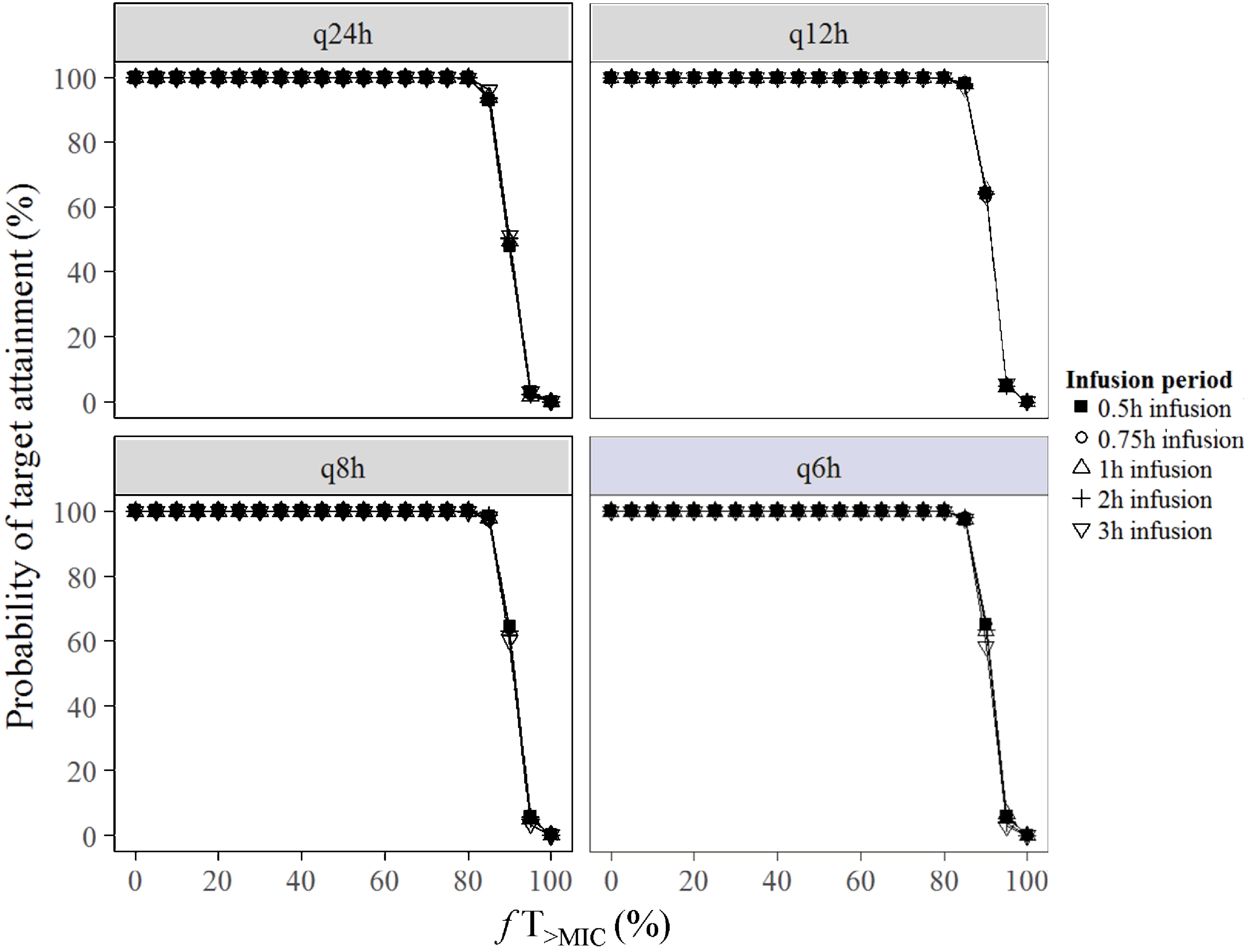

Figure S21. Probability of target attainment (PTA) of benapenem at $\%f\mathrm{T}_{>\mathrm{MIC}}$ of 0% to 100% against *Enterobacter cloacae* under dose of 1000 mg with different infusion time and dose interval.

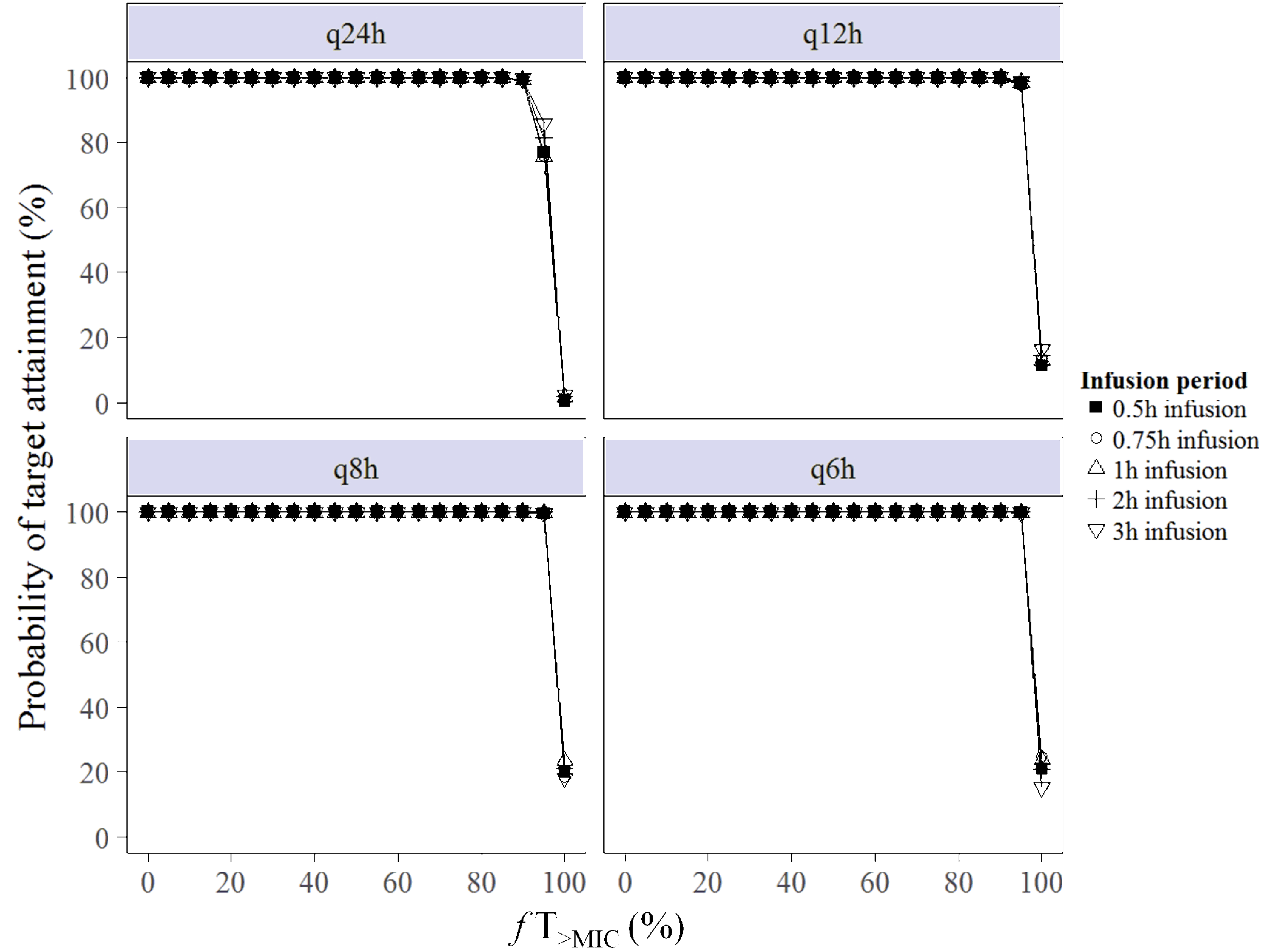

Figure S22. Probability of target attainment (PTA) of benapenem at %$fT_{>MIC}$ of 0% to 100% against ESBL+*E.coli* under dose of 250 mg with different infusion time and dose interval.

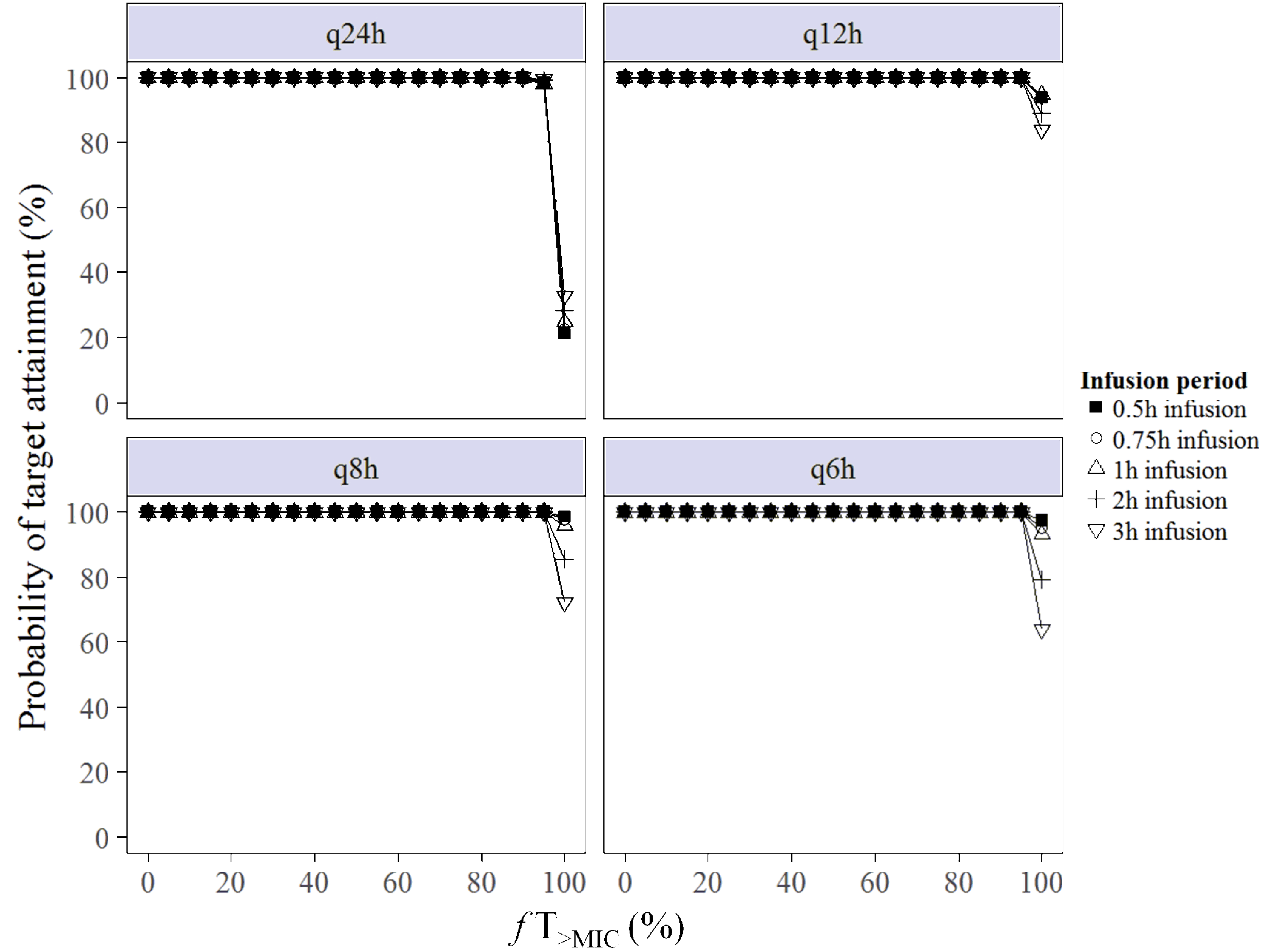

Figure S23. Probability of target attainment (PTA) of benapenem at $\%fT_{>MIC}$ of 0% to 100% against ESBL+*E.coli* under dose of 500 mg with different infusion time and dose interval.

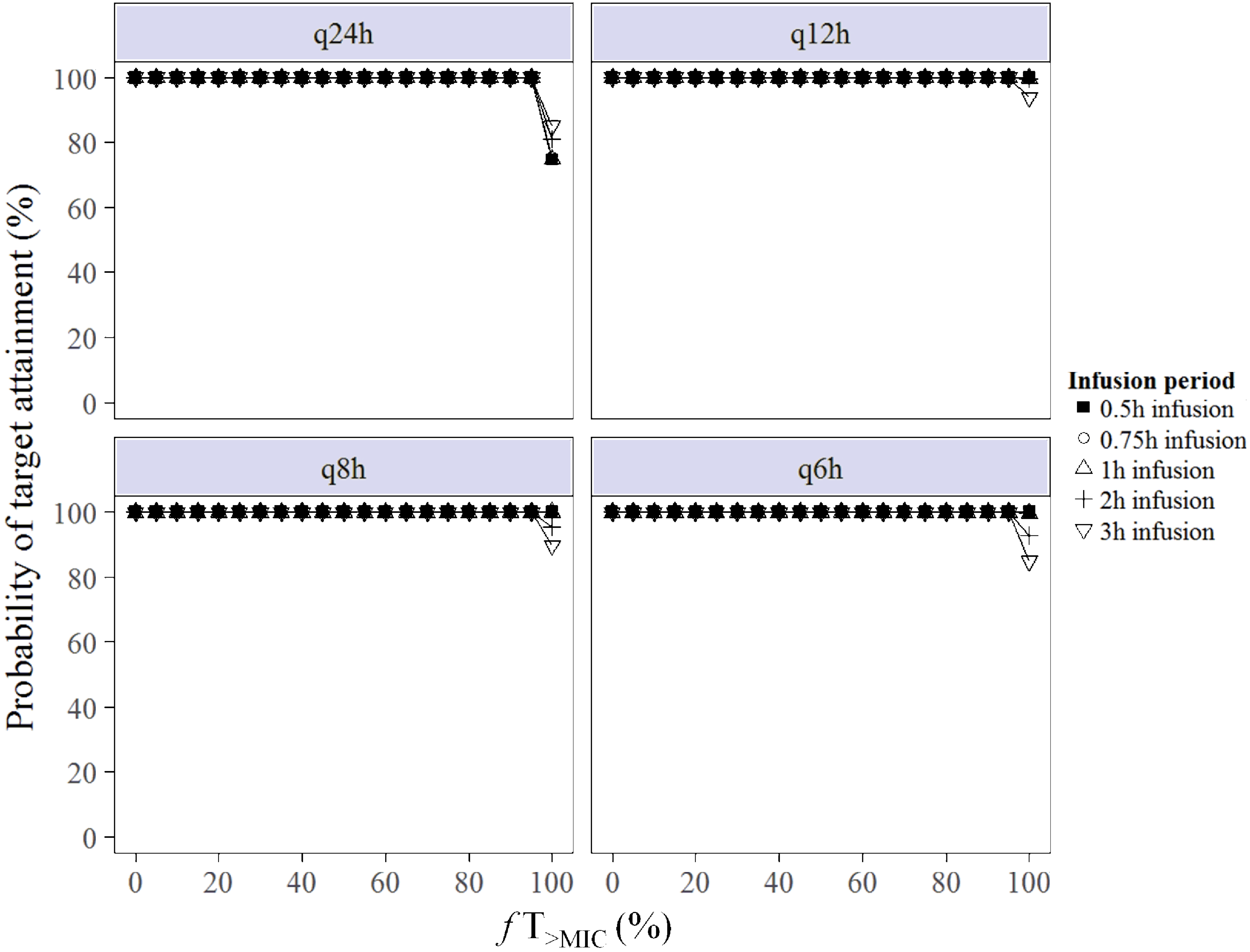

Figure S24. Probability of target attainment (PTA) of benapenem at %$fT_{>MIC}$ of 0% to 100% against ESBL+*E.coli* under dose of 1000 mg with different infusion time and dose interval.

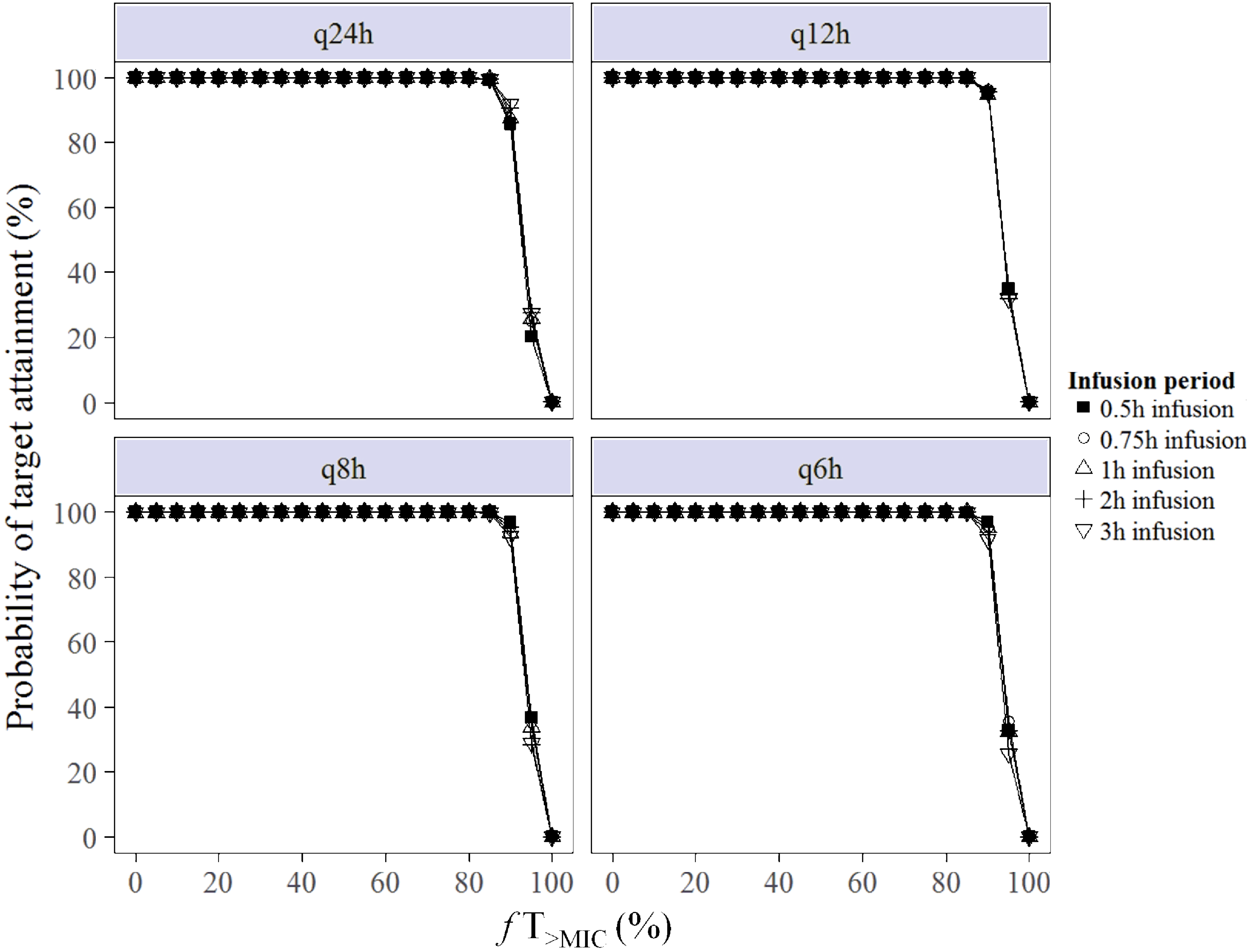

Figure S25. Probability of target attainment (PTA) of benapenem at %fT$_{>MIC}$ of 0% to 100% against ESBL+*K.pneumoniae* under dose of 250 mg with different infusion time and dose interval.

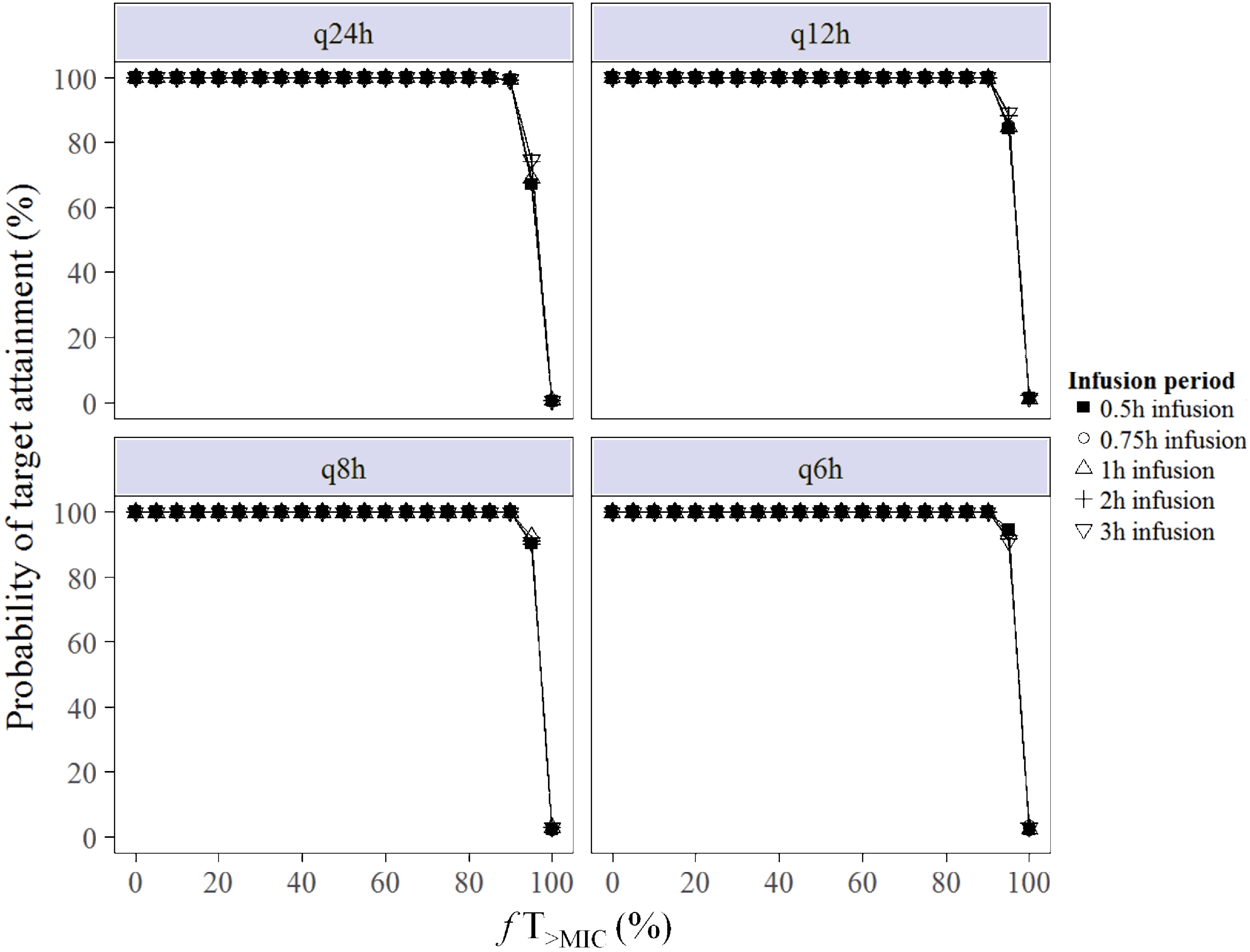

Figure S26. Probability of target attainment (PTA) of benapenem at %$fT_{>MIC}$ of 0% to 100% against ESBL+*K.pneumoniae* under dose of 500 mg with different infusion time and dose interval.

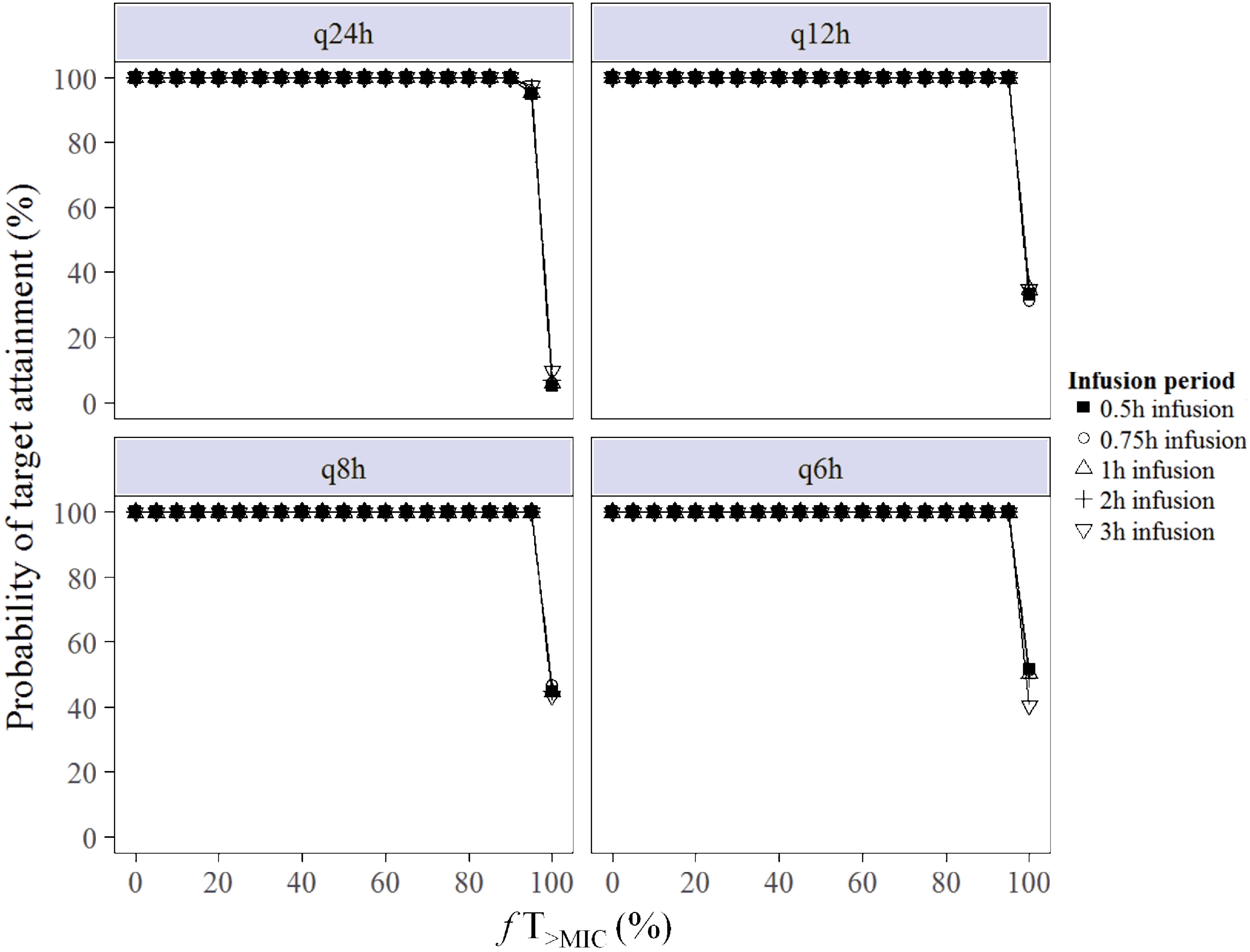

Figure S27. Probability of target attainment (PTA) of benapenem at $\% fT_{>MIC}$ of 0% to 100% against ESBL+*K.pneumoniae* under dose of 1000 mg with different infusion time and dose interval.

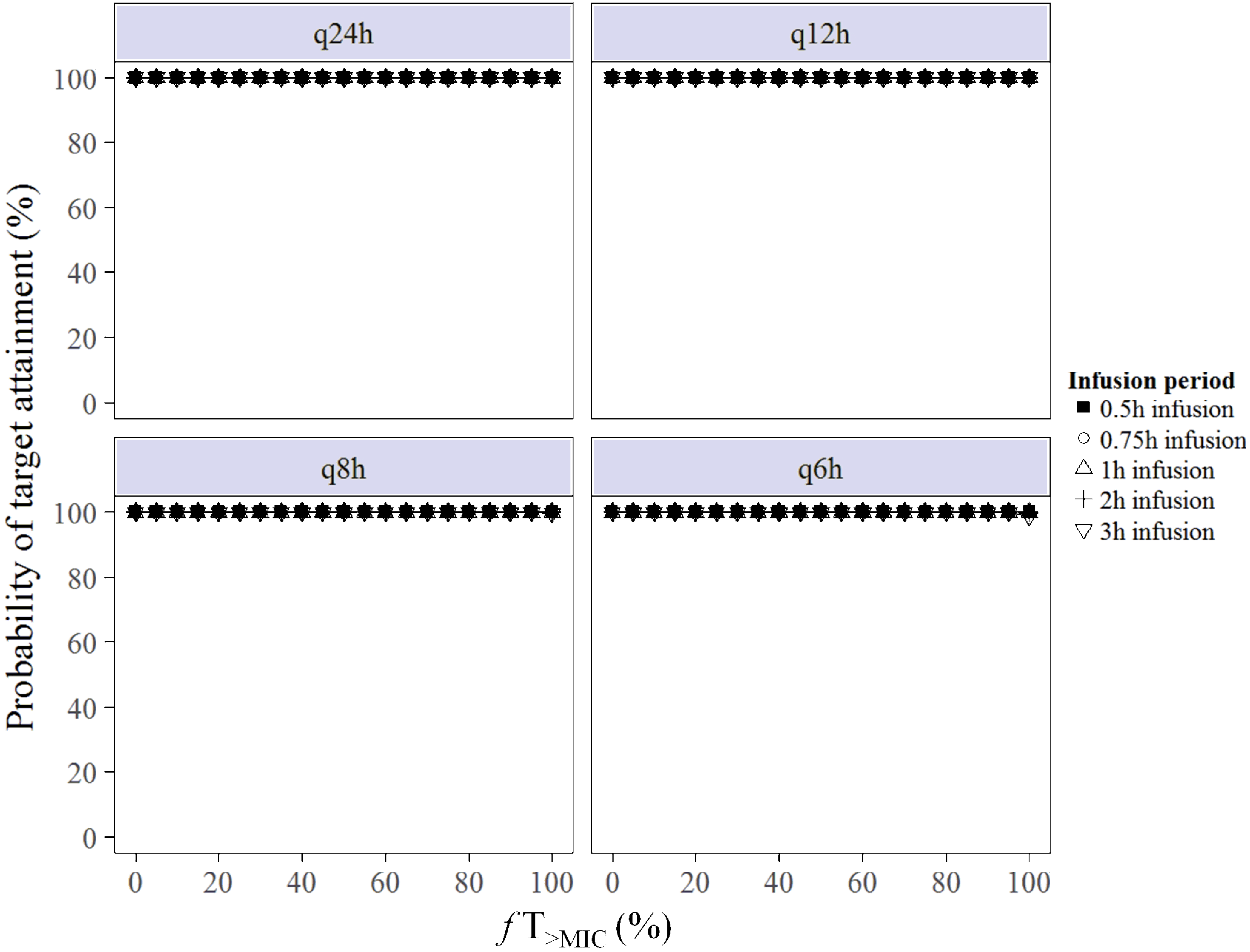

Figure S28. Probability of target attainment (PTA) of benapenem at $\%fT_{>MIC}$ of 0% to 100% against ESBL-*E.coli* under dose of 250 mg with different infusion time and dose interval.

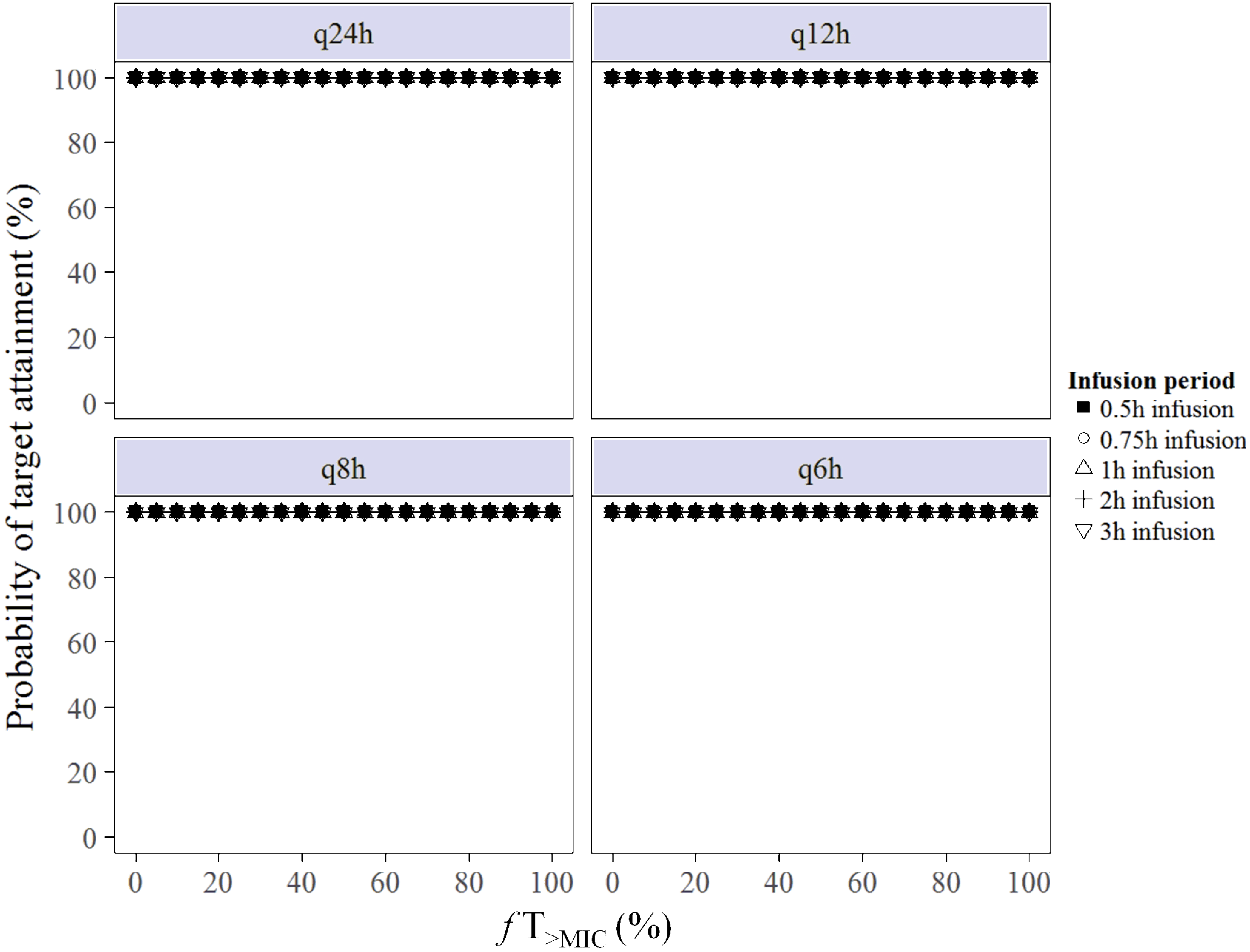

Figure S29. Probability of target attainment (PTA) of benapenem at %$fT_{>MIC}$ of 0% to 100% against ESBL-*E.coli* under dose of 500 mg with different infusion time and dose interval.

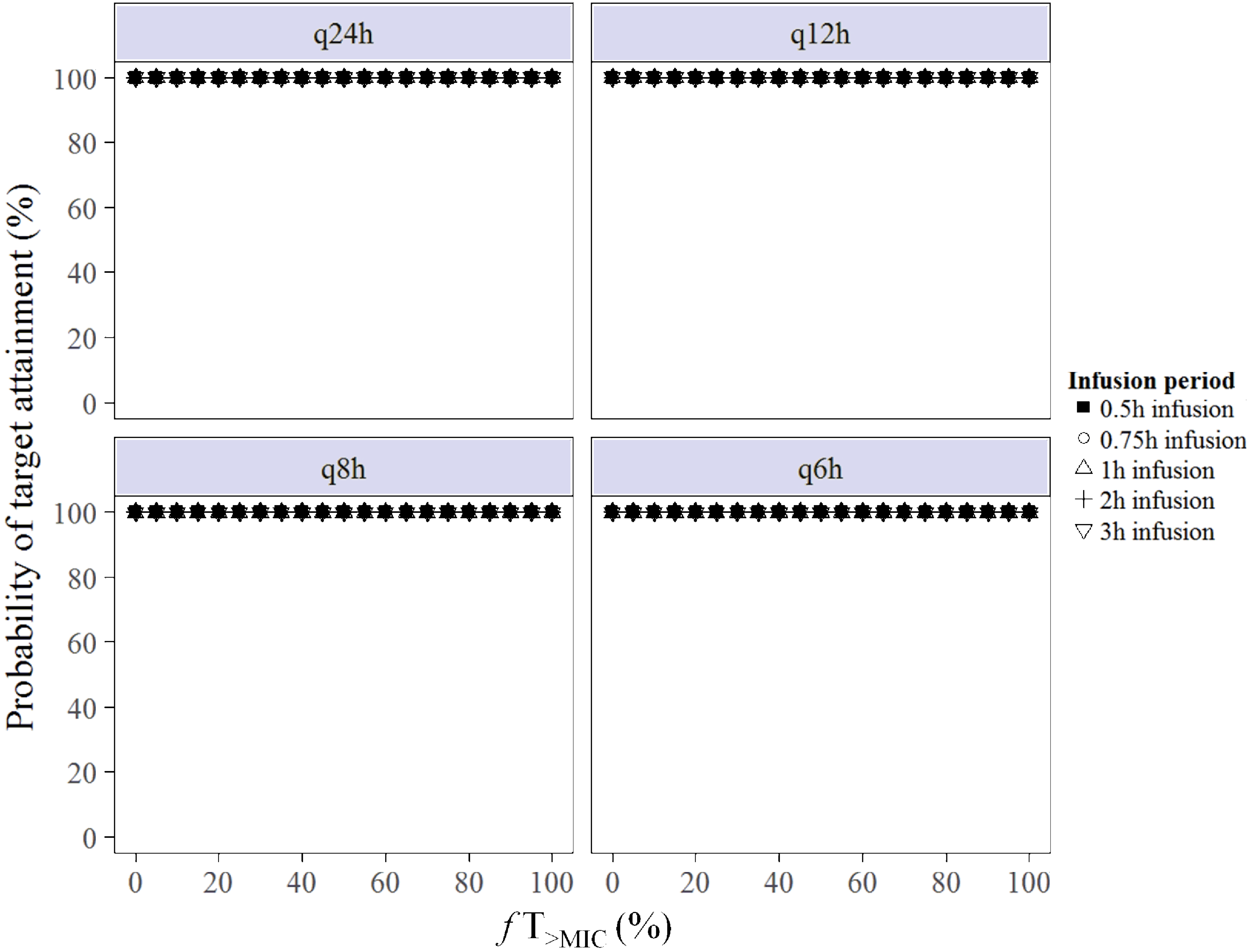

Figure S30. Probability of target attainment (PTA) of benapenem at %$fT_{>MIC}$ of 0% to 100% against ESBL-*E.coli* under dose of 1000 mg with different infusion time and dose interval.

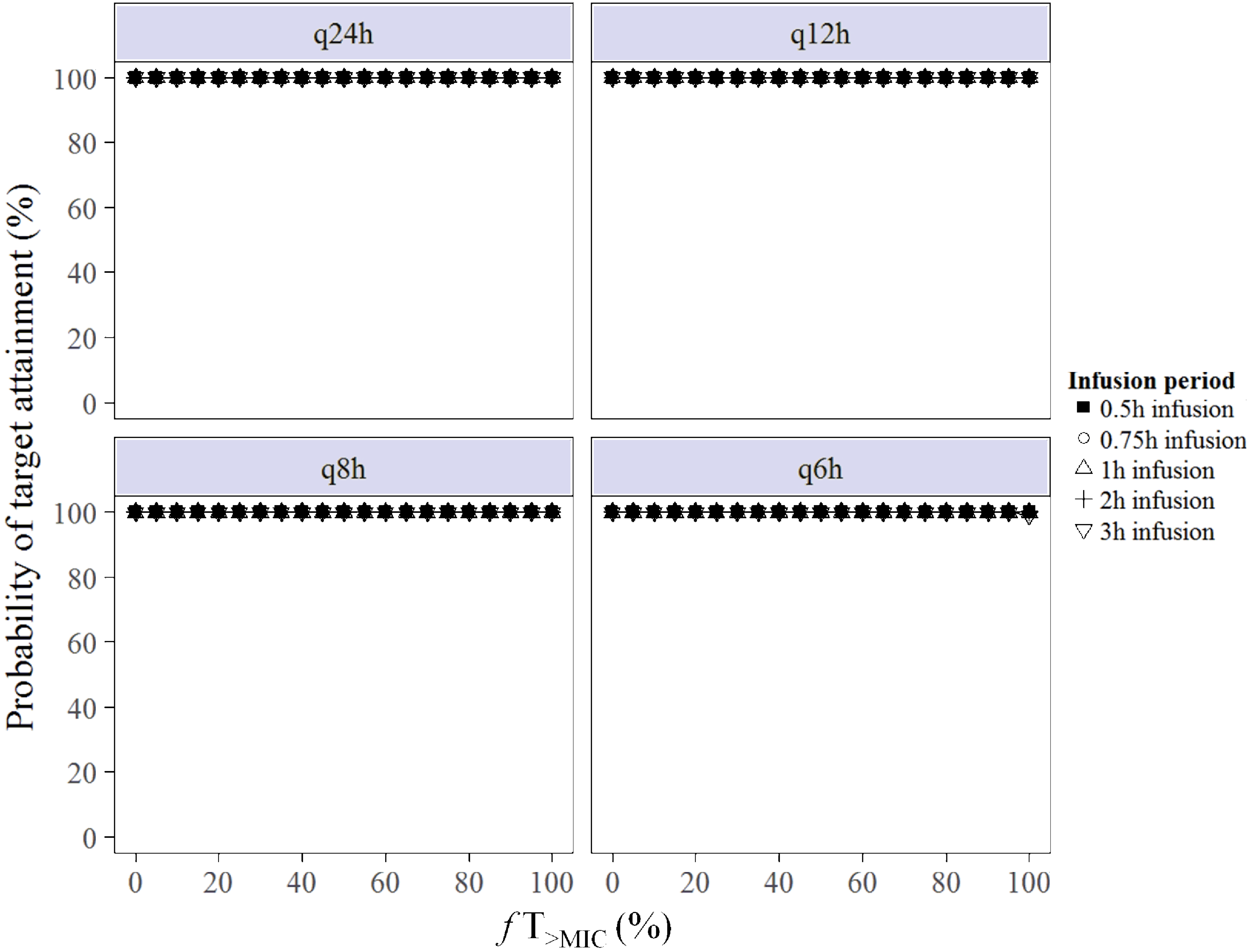

Figure S31. Probability of target attainment (PTA) of benapenem at $\%fT_{>MIC}$ of 0% to 100% against ESBL-*K.pneumoniae* under dose of 250 mg with different infusion time and dose interval.

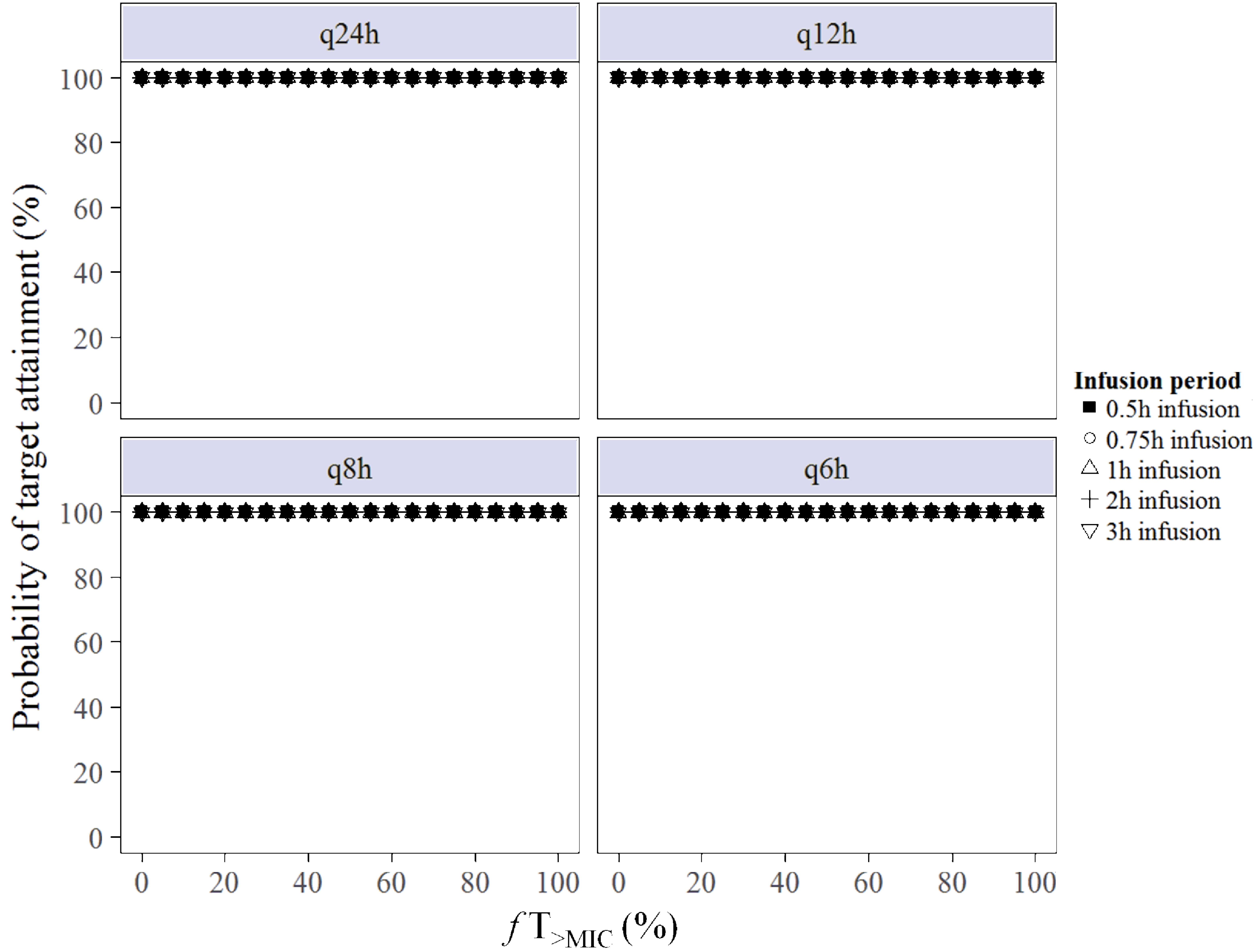

Figure S32. Probability of target attainment (PTA) of benapenem at $\%fT_{>MIC}$ of 0% to 100% against ESBL-*K.pneumoniae* under dose of 500 mg with different infusion time and dose interval.

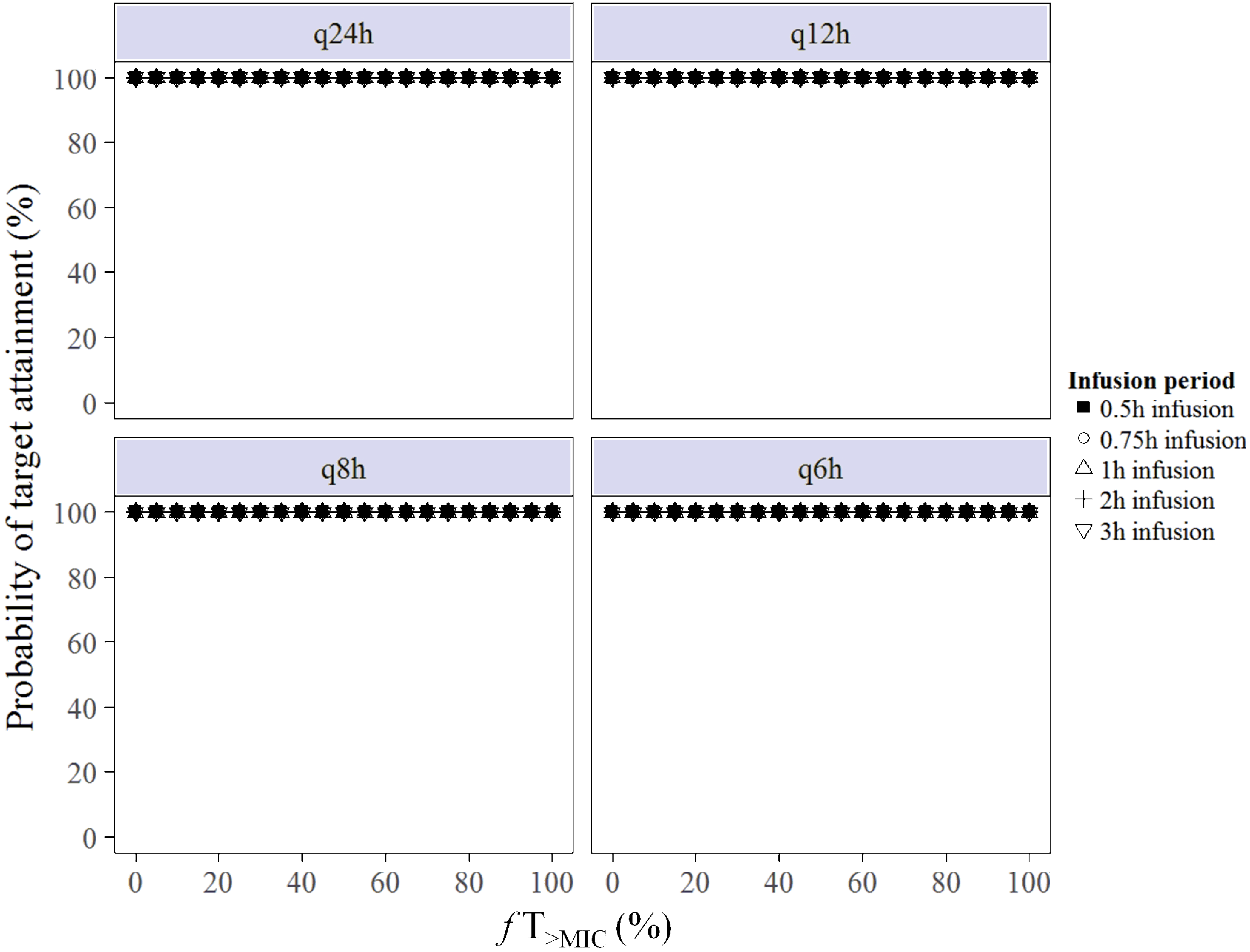

Figure S33. Probability of target attainment (PTA) of benapenem at $\%fT_{>MIC}$ of 0% to 100% against ESBL-*K.pneumoniae* under dose of 1000 mg with different infusion time and dose interval.

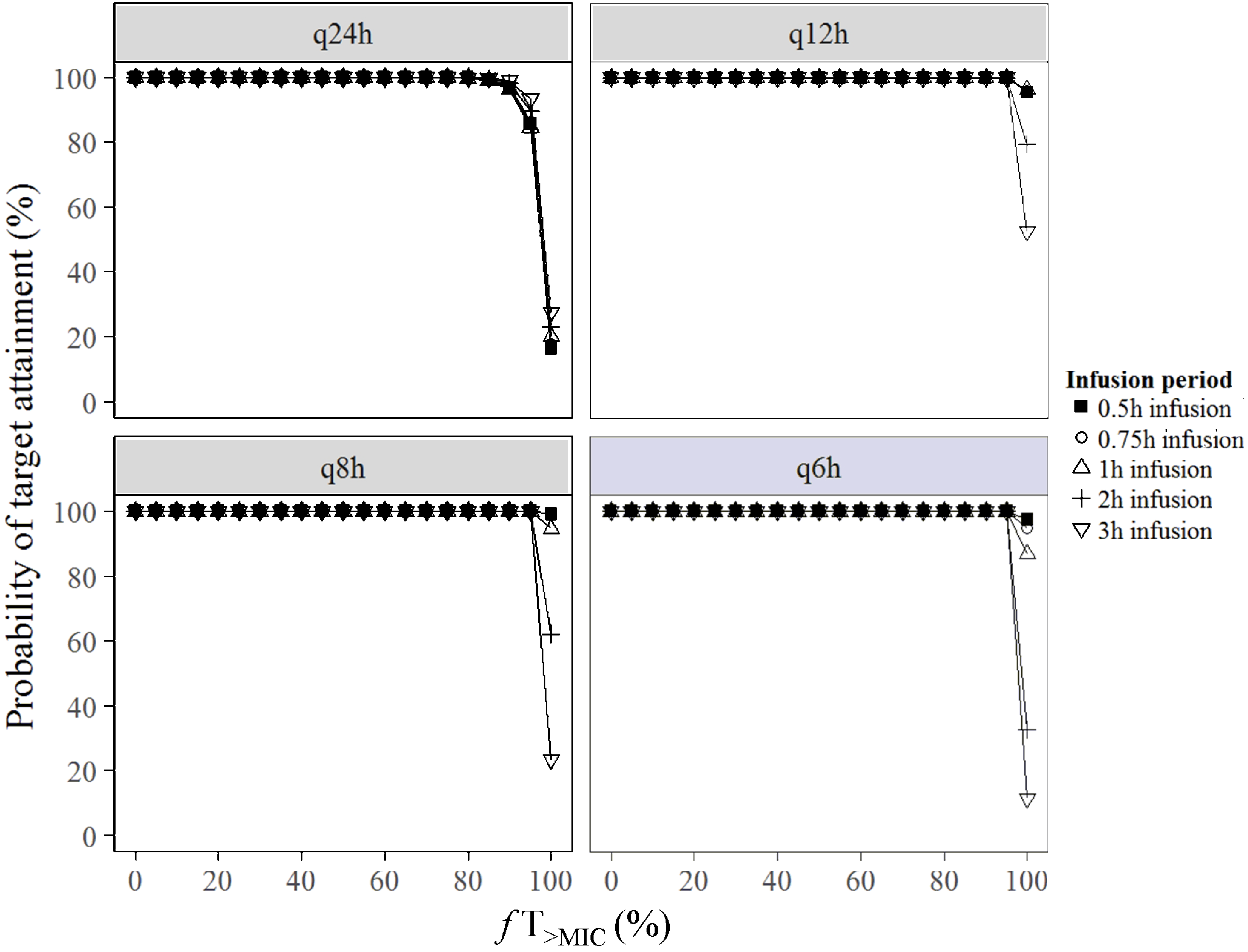

Figure S34. Probability of target attainment (PTA) of benapenem at %$f\mathrm{T}_{>\mathrm{MIC}}$ of 0% to 100% against *Morganella* under dose of 250 mg with different infusion time and dose interval.

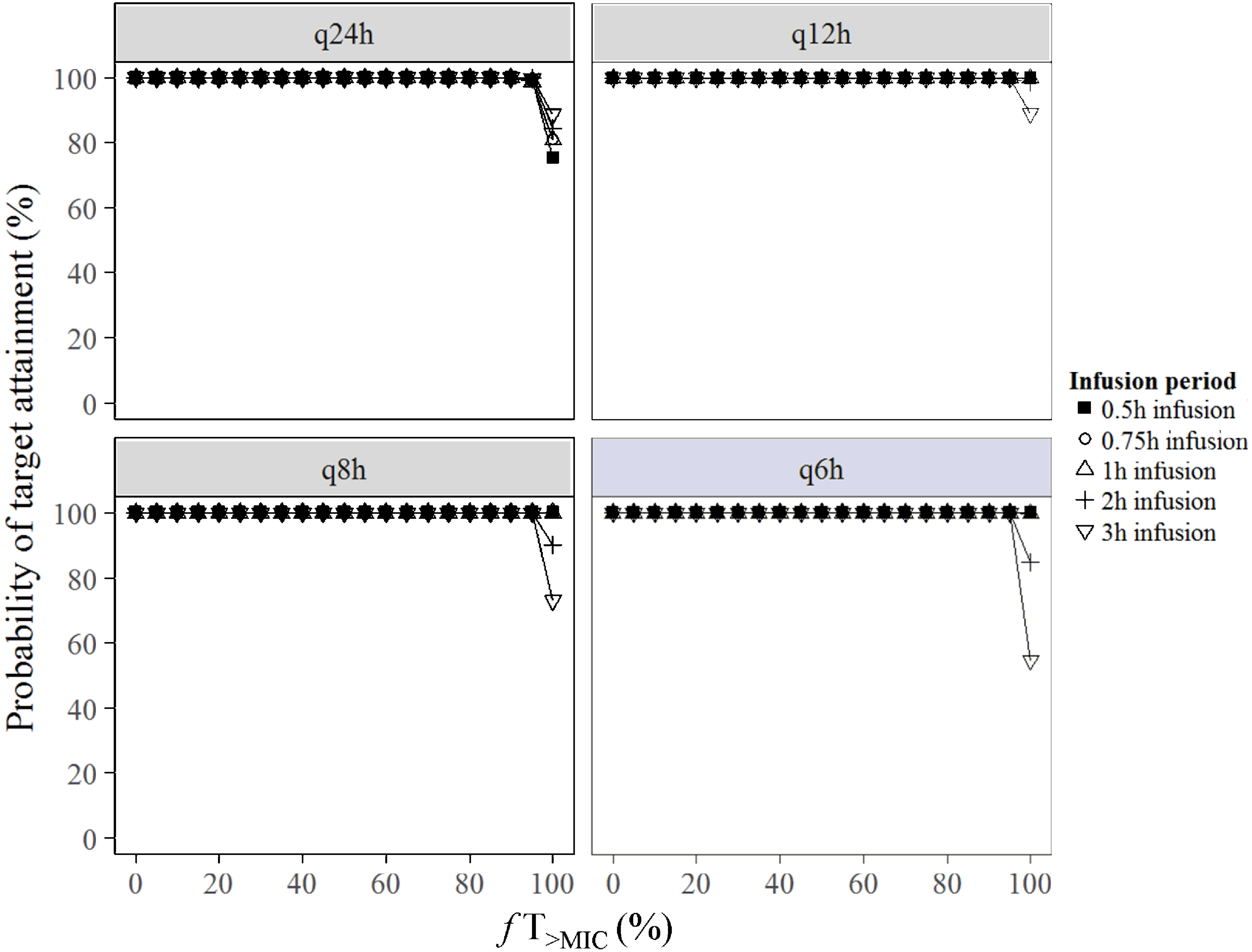

Figure S35. Probability of target attainment (PTA) of benapenem at %$fT_{>MIC}$ of 0% to 100% against *Morganella* under dose of 500 mg with different infusion time and dose interval.

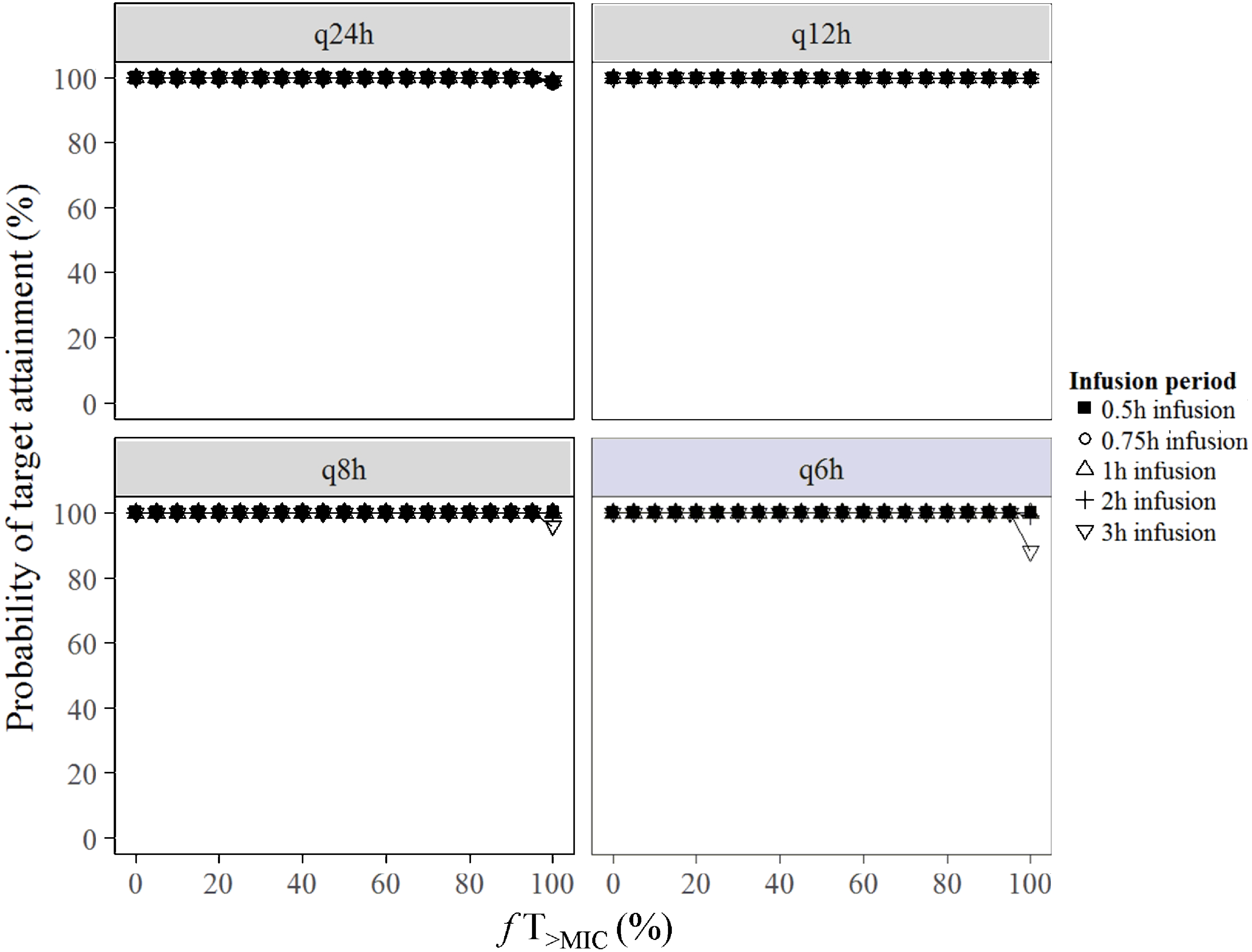

Figure S36. Probability of target attainment (PTA) of benapenem at %$fT_{>MIC}$ of 0% to 100% against *Morganella* under dose of 1000 mg with different infusion time and dose interval.

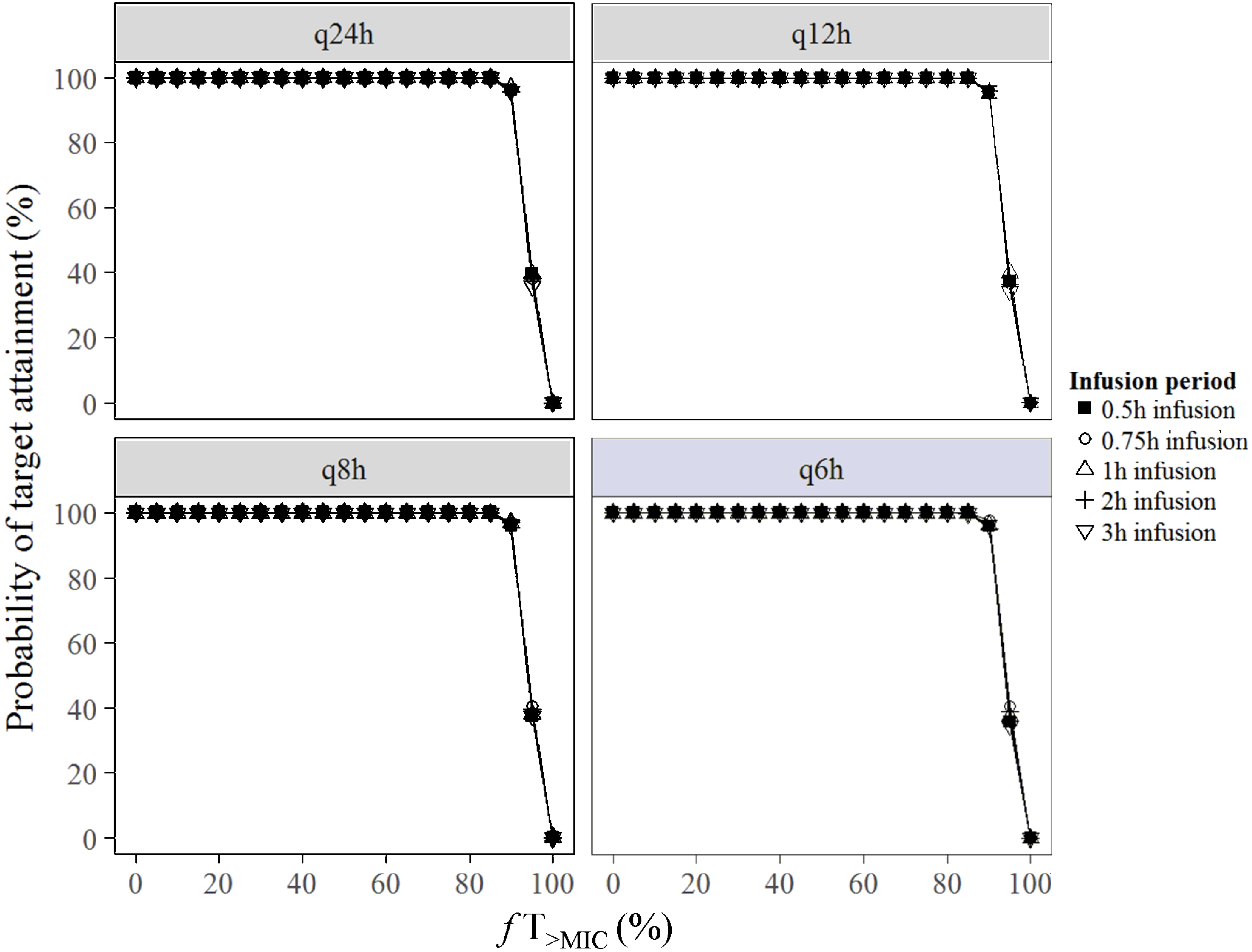

Figure S37. Probability of target attainment (PTA) of benapenem at %$fT_{>MIC}$ of 0% to 100% against Other *Klebsiella* under dose of 250 mg with different infusion time and dose interval.

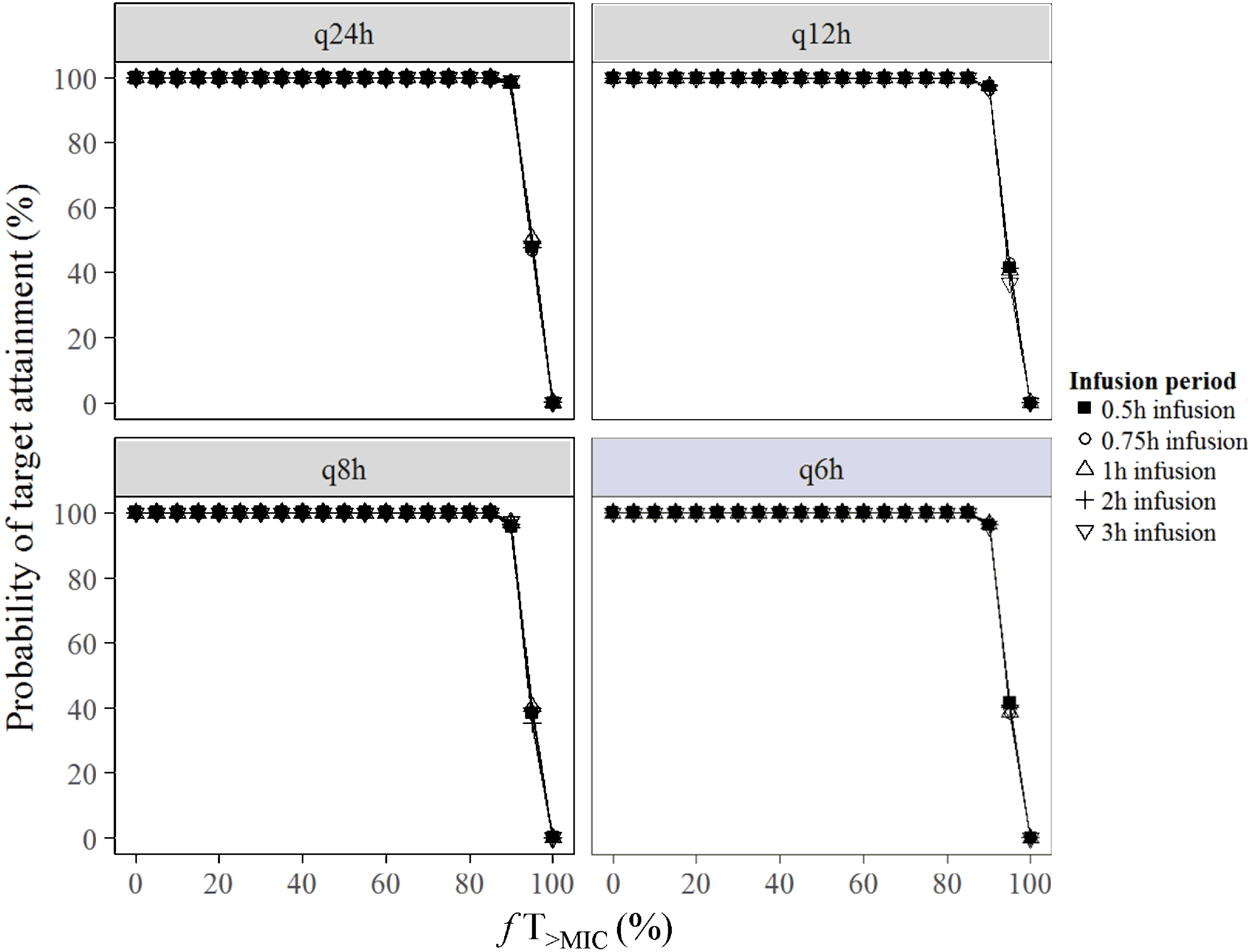

Figure S38. Probability of target attainment (PTA) of benapenem at %$fT_{>MIC}$ of 0% to 100% against Other *Klebsiella* under dose of 500 mg with different infusion time and dose interval.

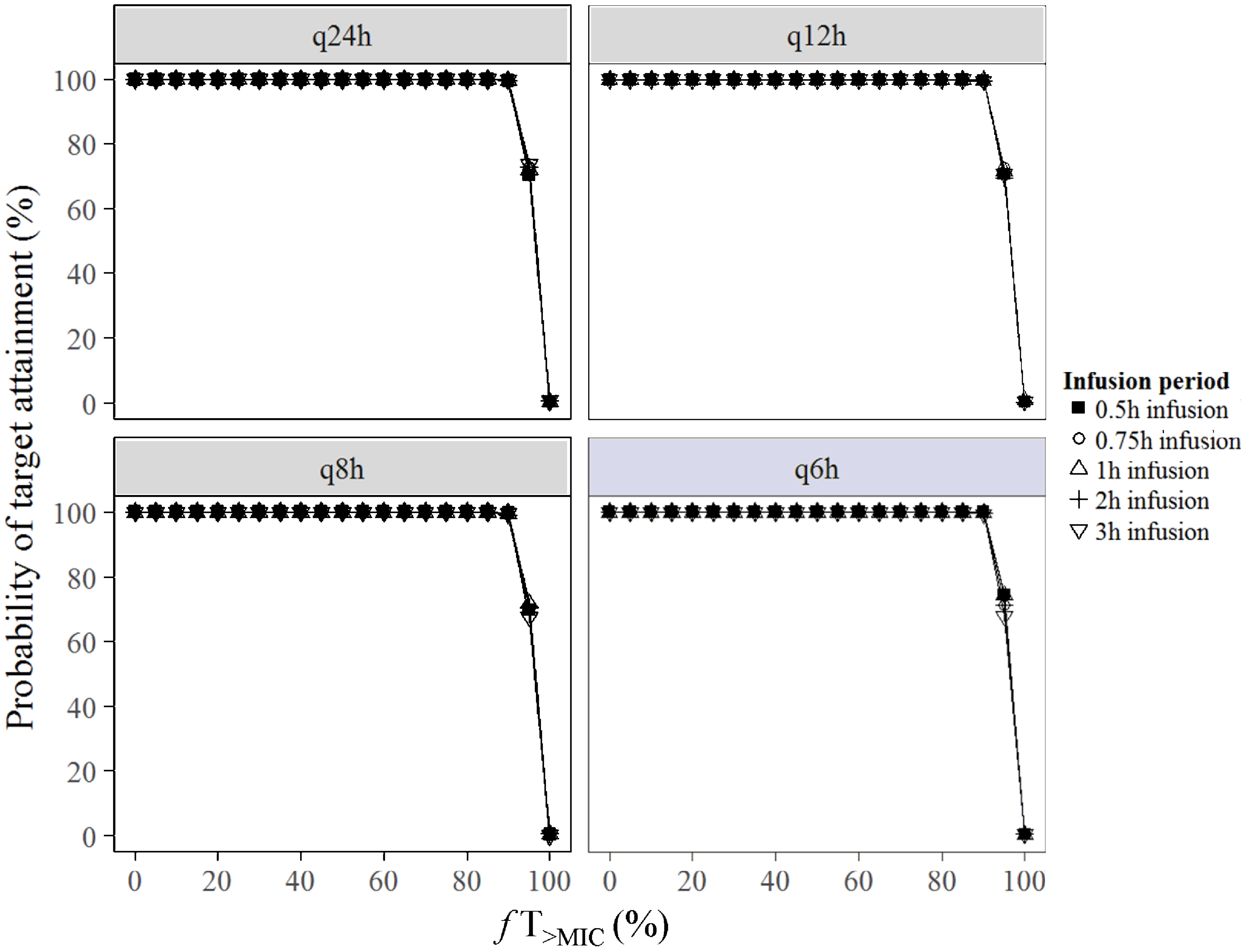

Figure S39. Probability of target attainment (PTA) of benapenem at %fT$_{>MIC}$ of 0% to 100% against Other *Klebsiella* under dose of 1000 mg with different infusion time and dose interval.

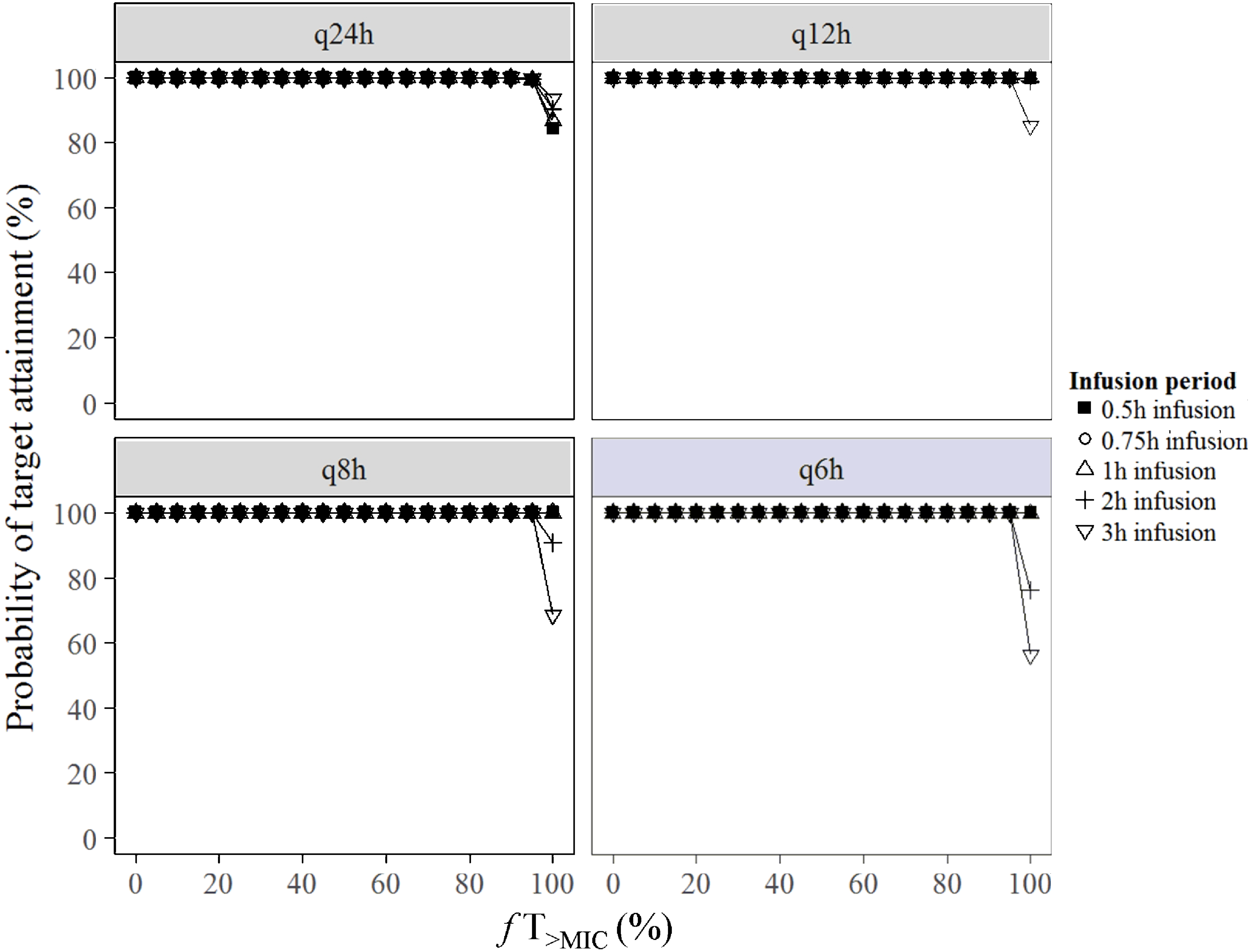

Figure S40. Probability of target attainment (PTA) of benapenem at %$fT_{>MIC}$ of 0% to 100% against *Proteus* under dose of 250 mg with different infusion time and dose interval.

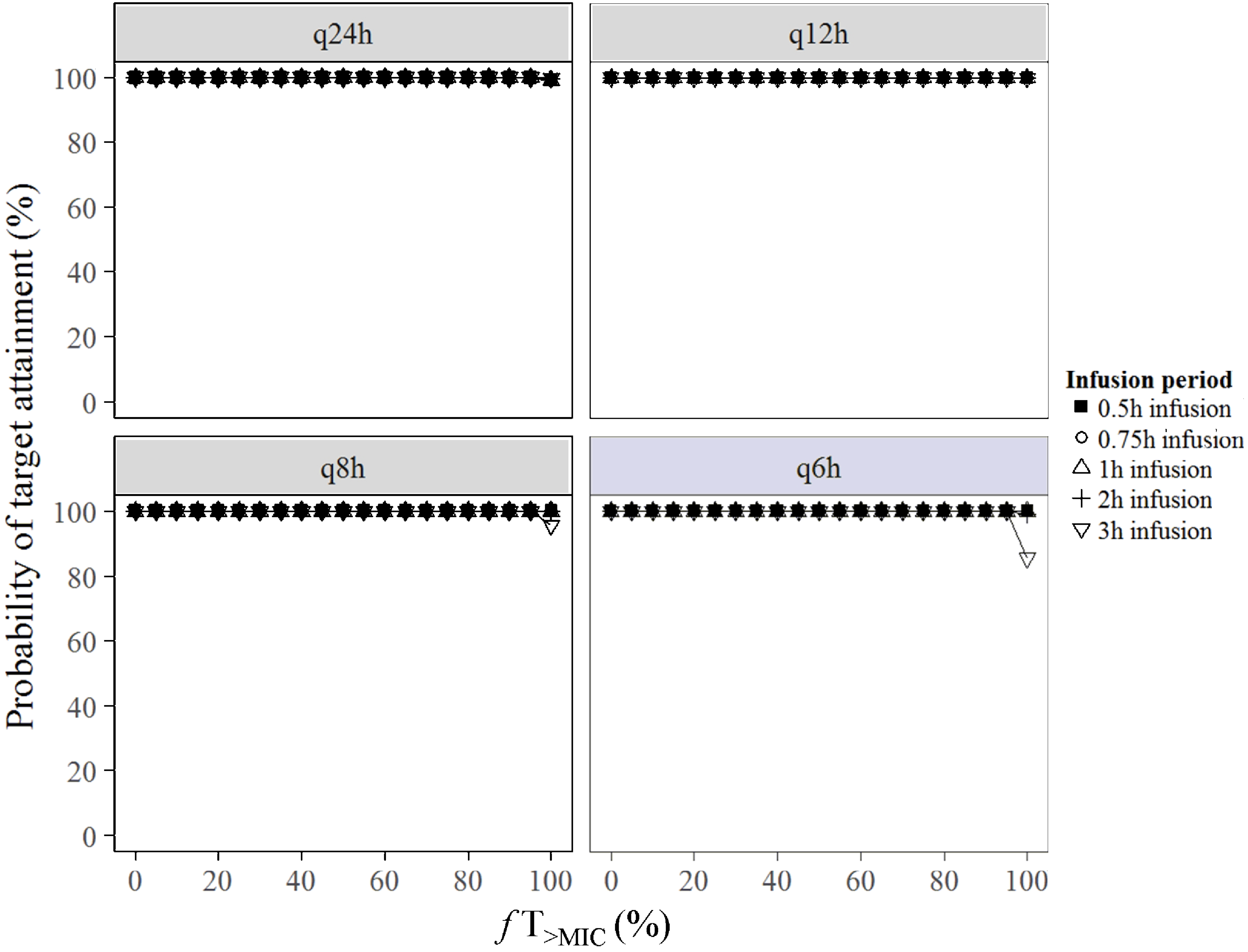

Figure S41. Probability of target attainment (PTA) of benapenem at %$f\mathrm{T}_{>\mathrm{MIC}}$ of 0% to 100% against *Proteus* under dose of 500 mg with different infusion time and dose interval.

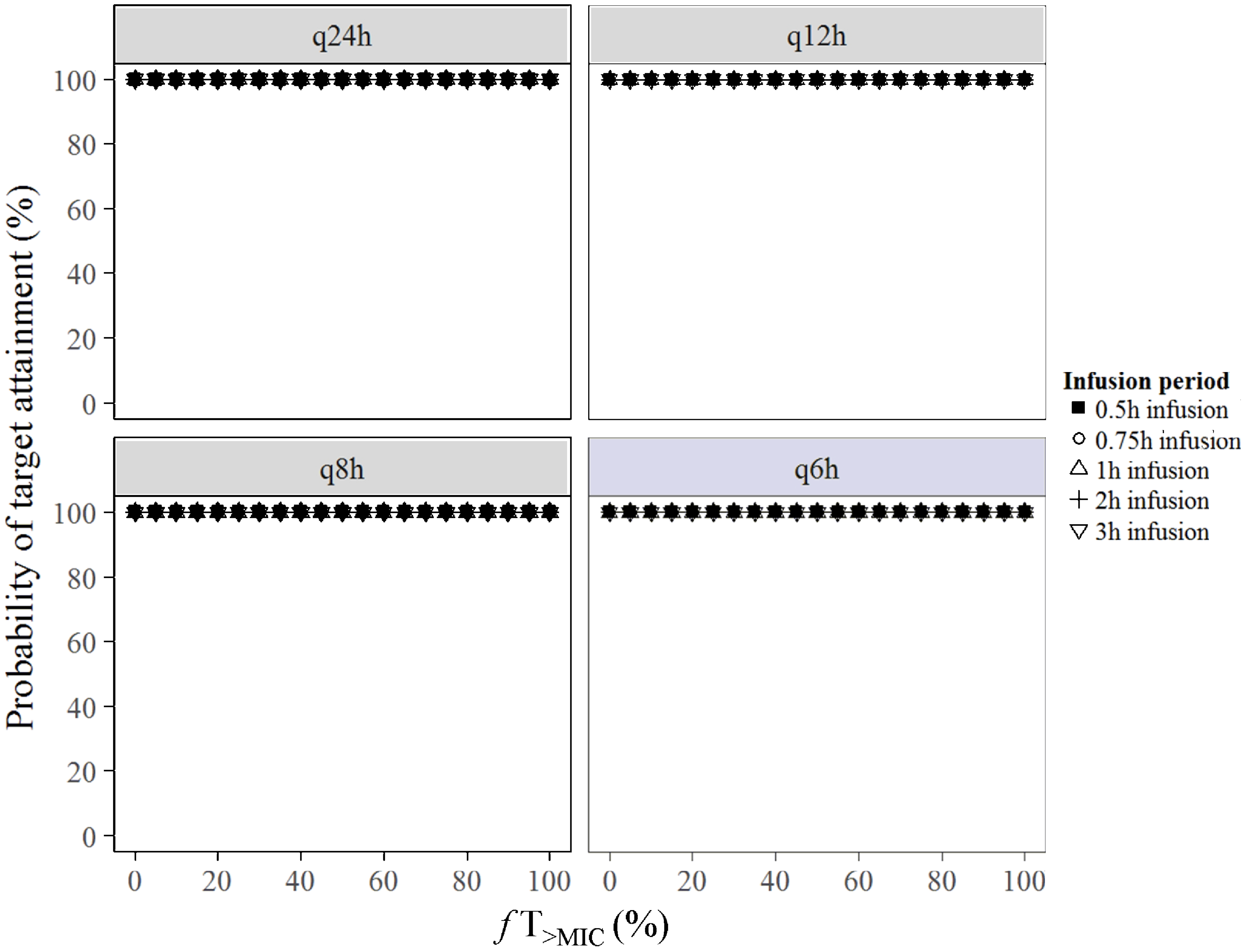

Figure S42. Probability of target attainment (PTA) of benapenem at $\%fT_{>MIC}$ of 0% to 100% against *Proteus* under dose of 1000 mg with different infusion time and dose interval.

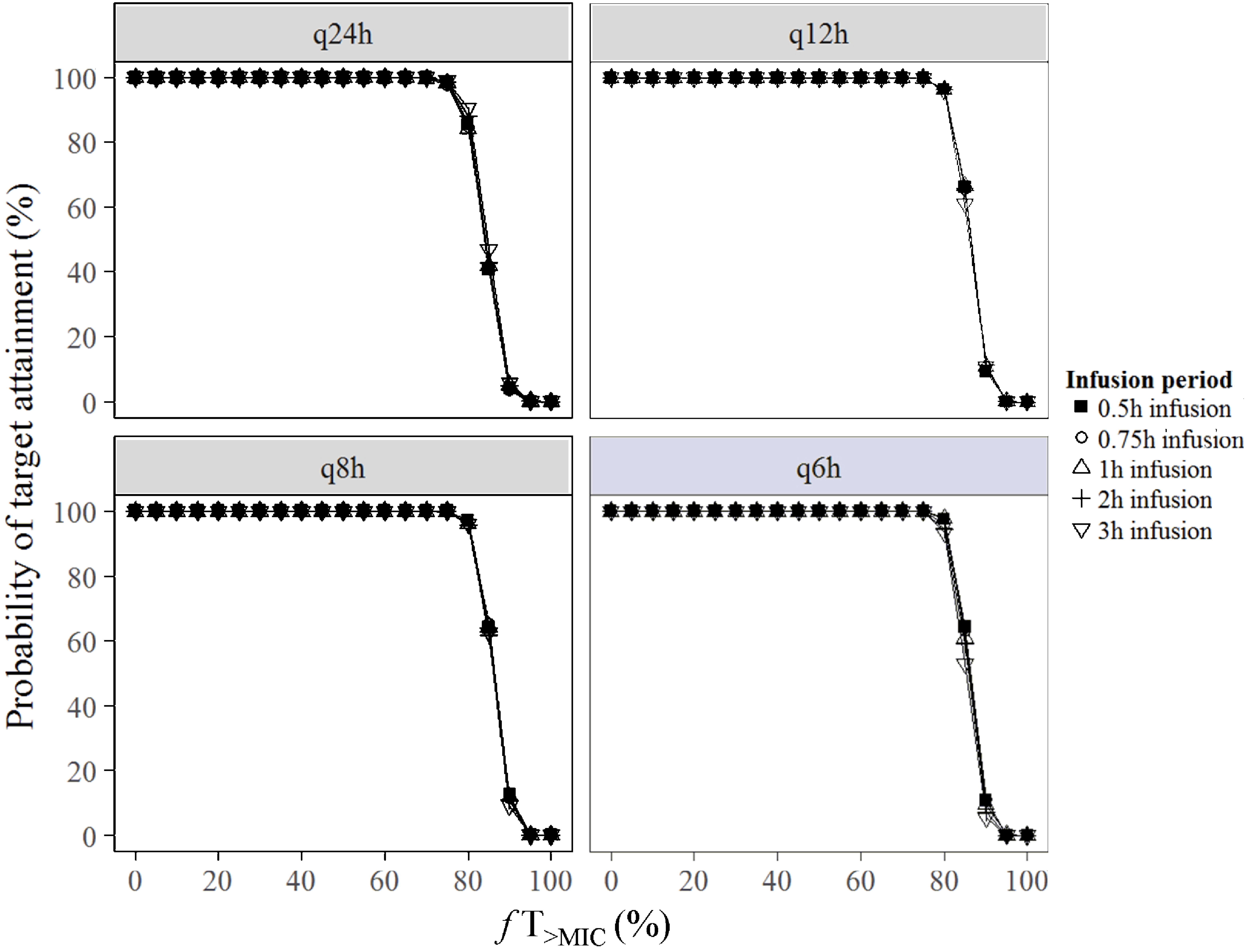

Figure S43. Probability of target attainment (PTA) of benapenem at $\%f\mathrm{T}_{>\mathrm{MIC}}$ of 0% to 100% against *Providencia* under dose of 250 mg with different infusion time and dose interval.

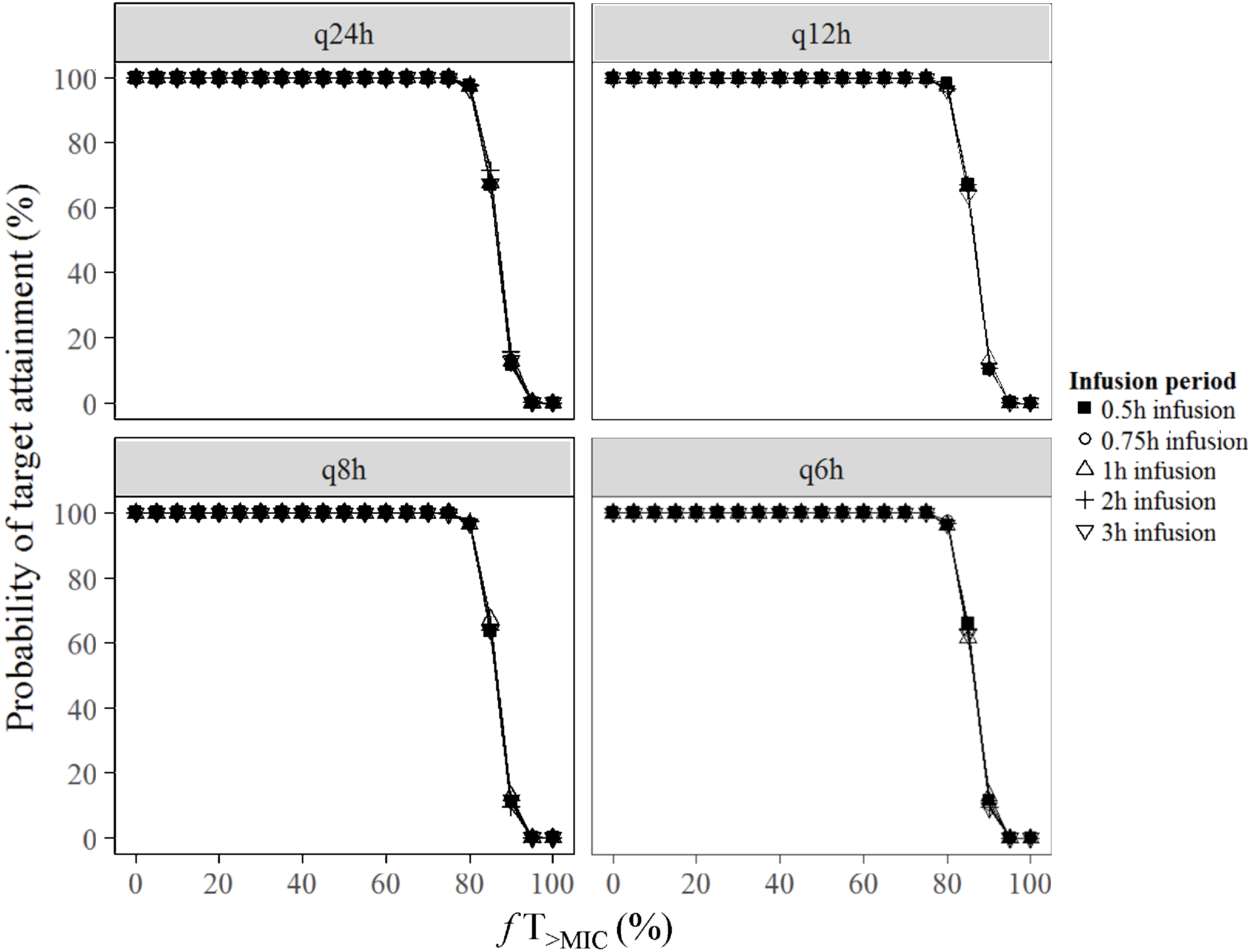

Figure S44. Probability of target attainment (PTA) of benapenem at %fT$_{>MIC}$ of 0% to 100% against *Providencia* under dose of 500 mg with different infusion time and dose interval.

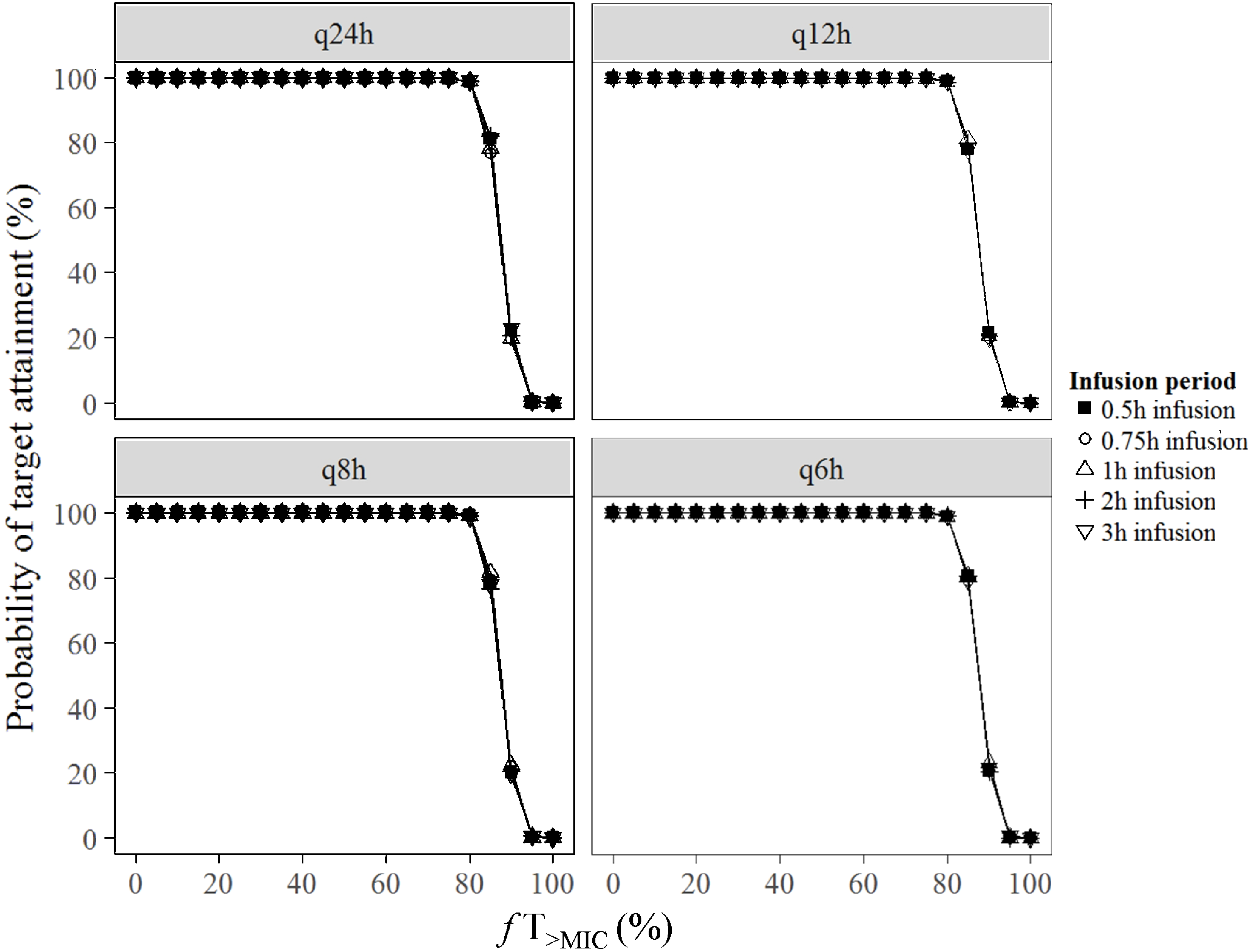

Figure S45. Probability of target attainment (PTA) of benapenem at %$f\mathrm{T}_{>\mathrm{MIC}}$ of 0% to 100% against *Providencia* under dose of 1000 mg with different infusion time and dose interval.

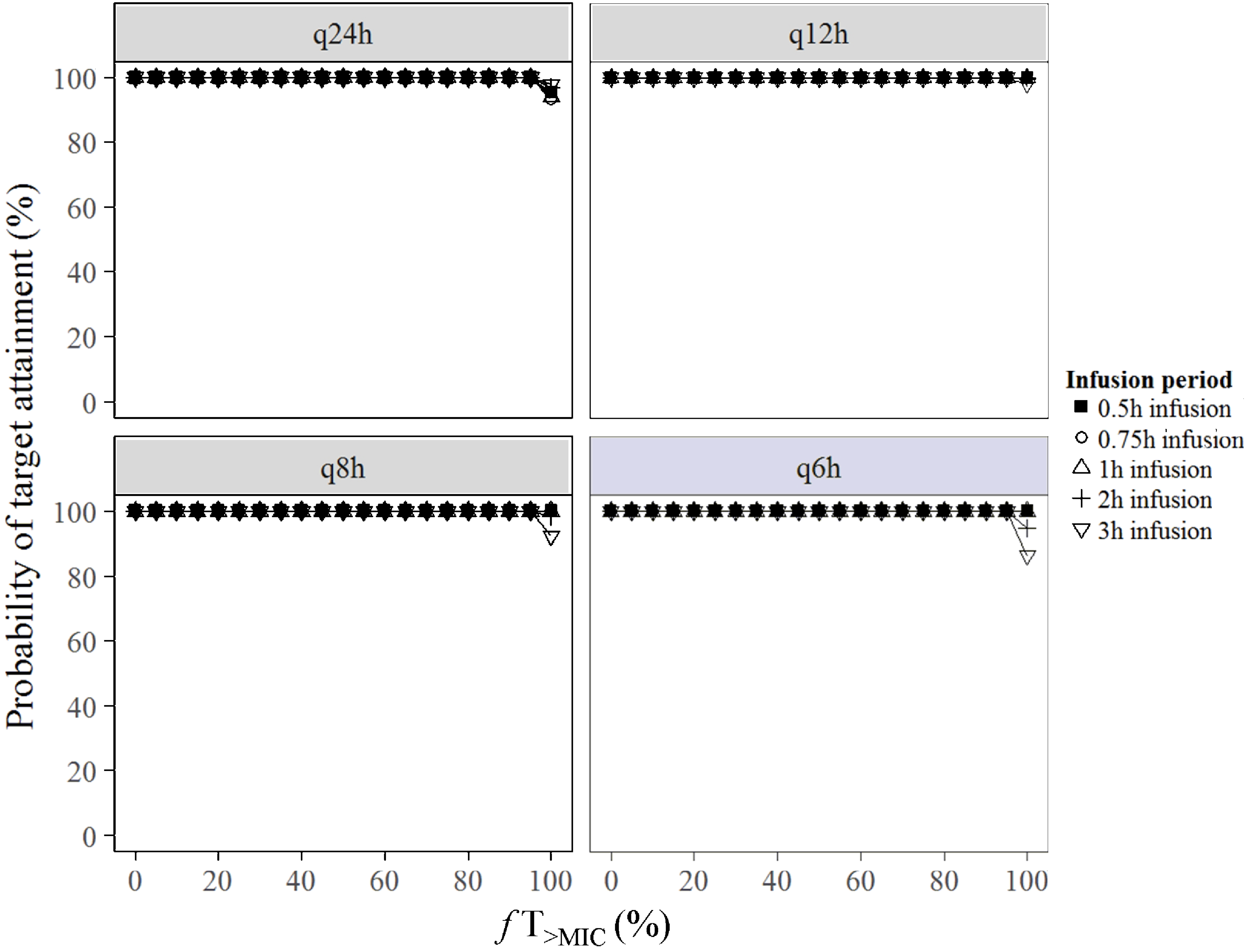

Figure S46. Probability of target attainment (PTA) of benapenem at %$fT_{>MIC}$ of 0% to 100% against *Salmonella* under dose of 250 mg with different infusion time and dose interval.

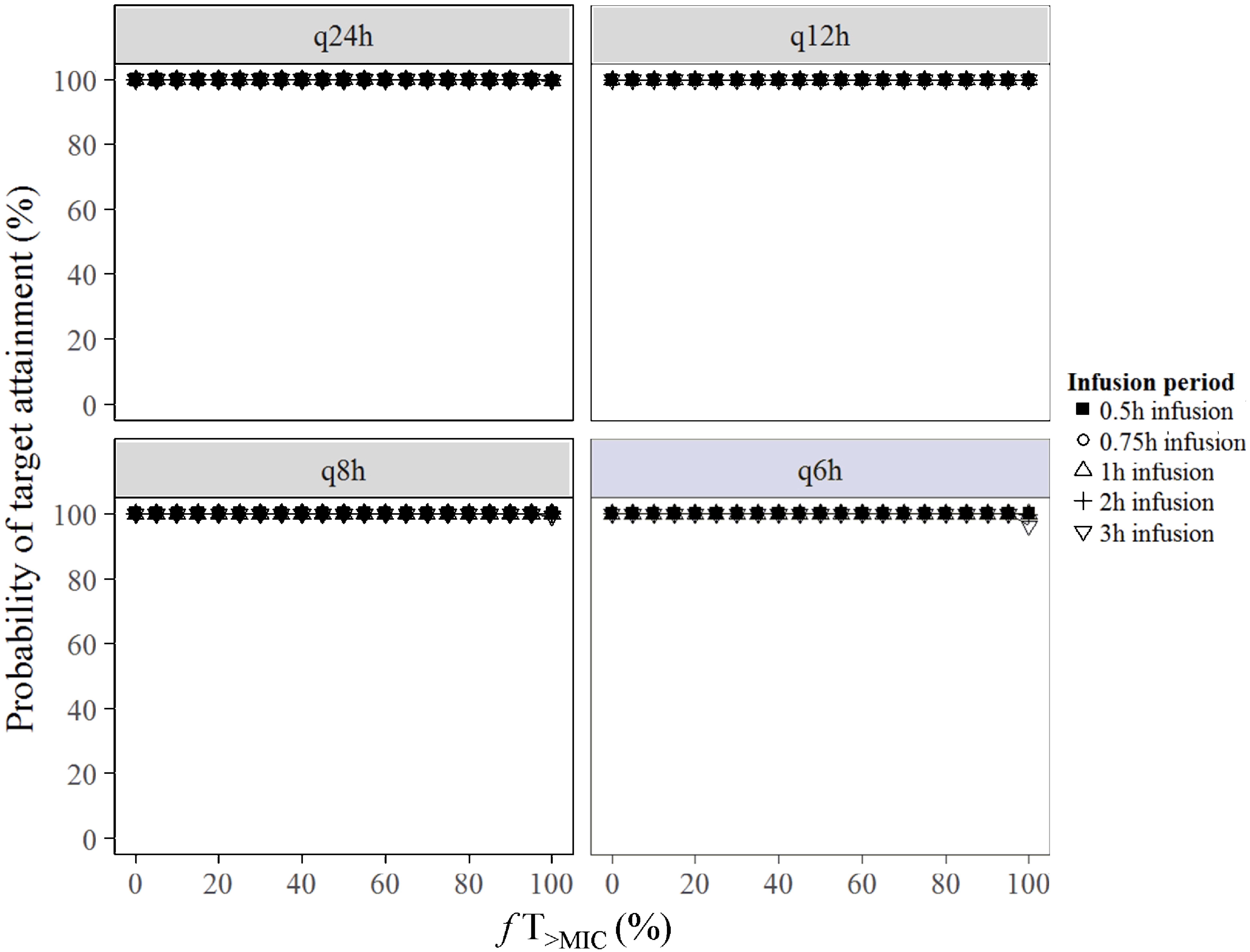

Figure S47. Probability of target attainment (PTA) of benapenem at %$fT_{>MIC}$ of 0% to 100% against *Salmonella* under dose of 500 mg with different infusion time and dose interval.

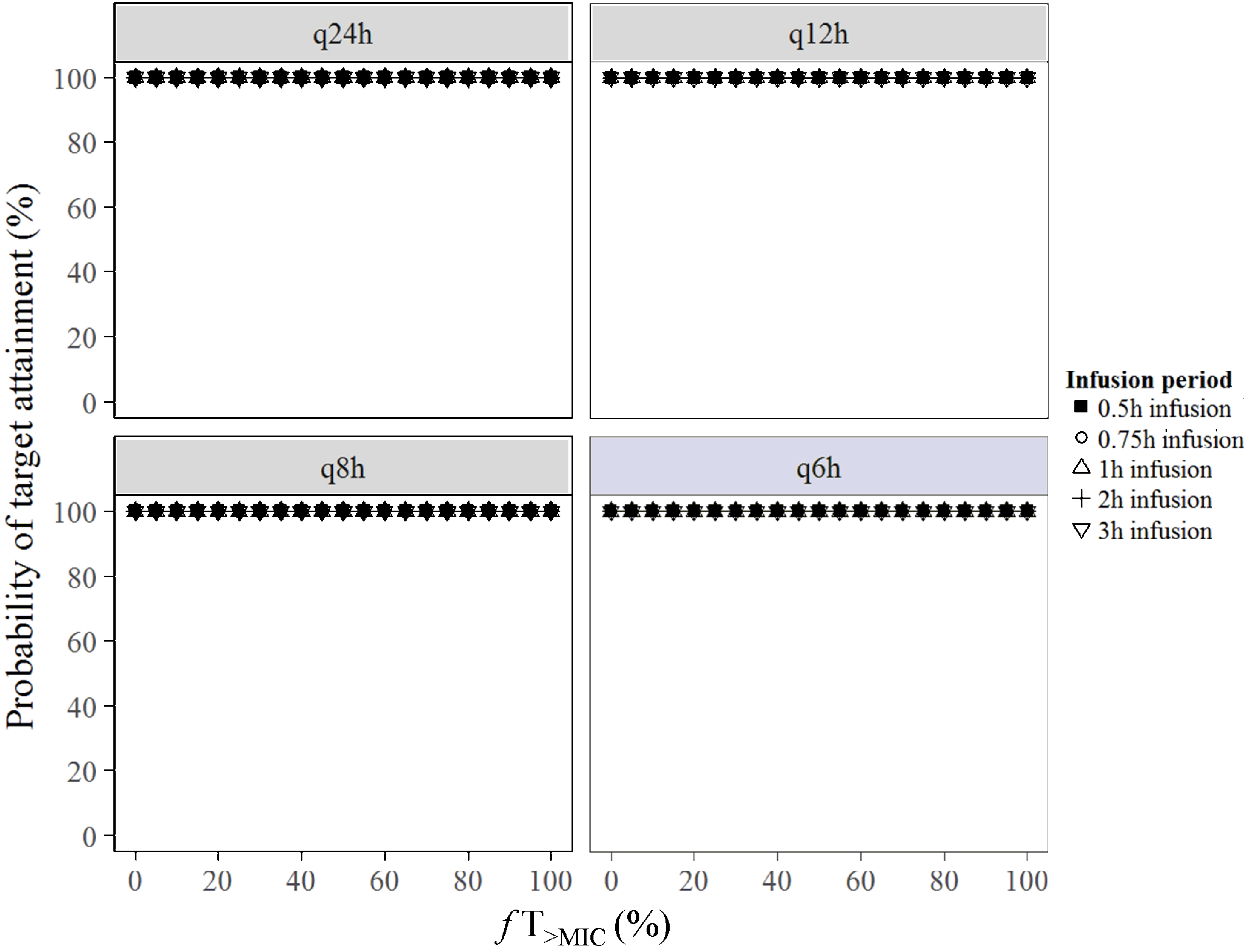

Figure S48. Probability of target attainment (PTA) of benapenem at %fT$_{>MIC}$ of 0% to 100% against *Salmonella* under dose of 1000 mg with different infusion time and dose interval.

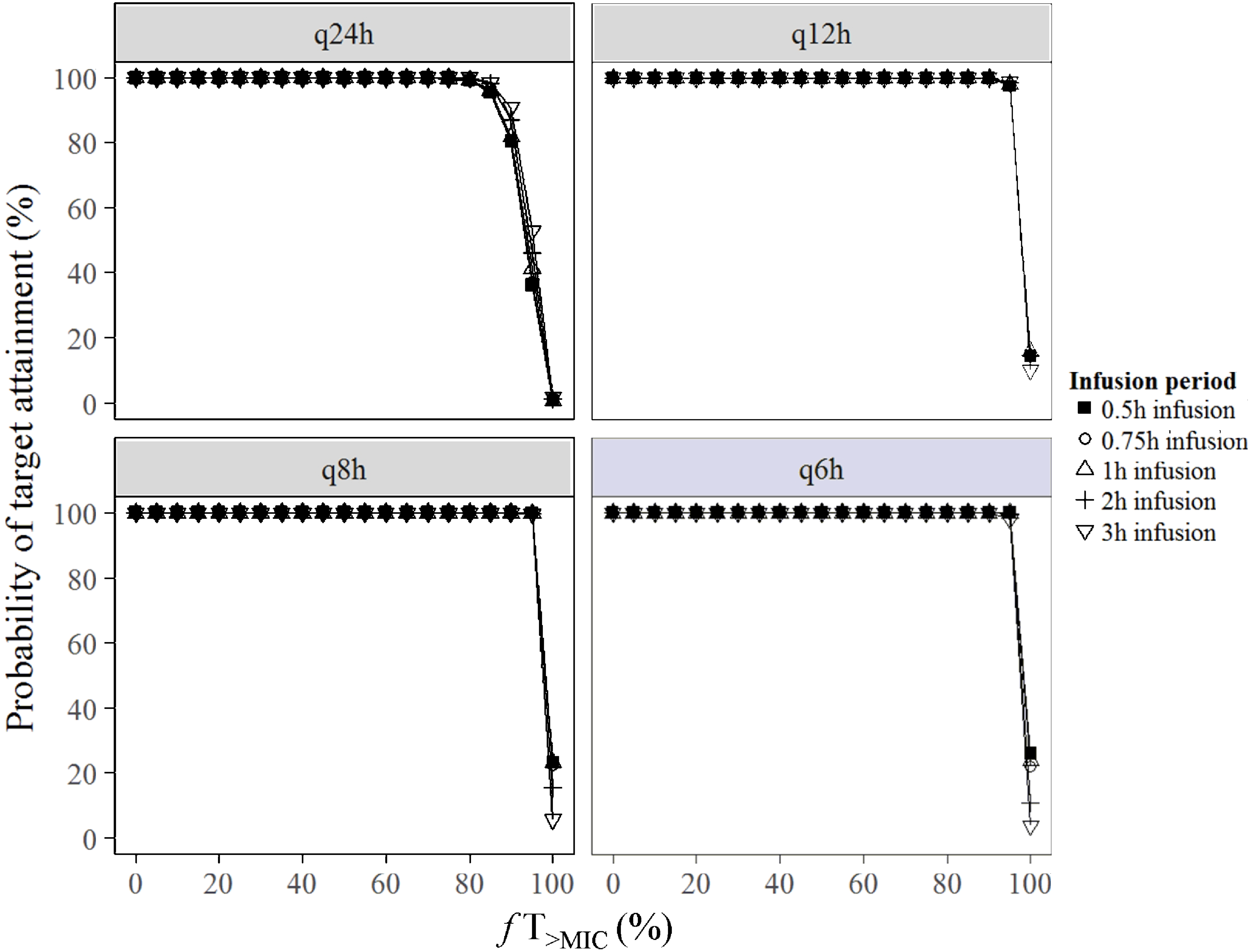

Figure S49. Probability of target attainment (PTA) of benapenem at %fT$_{>MIC}$ of 0% to 100% against *Serratia* under dose of 250 mg with different infusion time and dose interval.

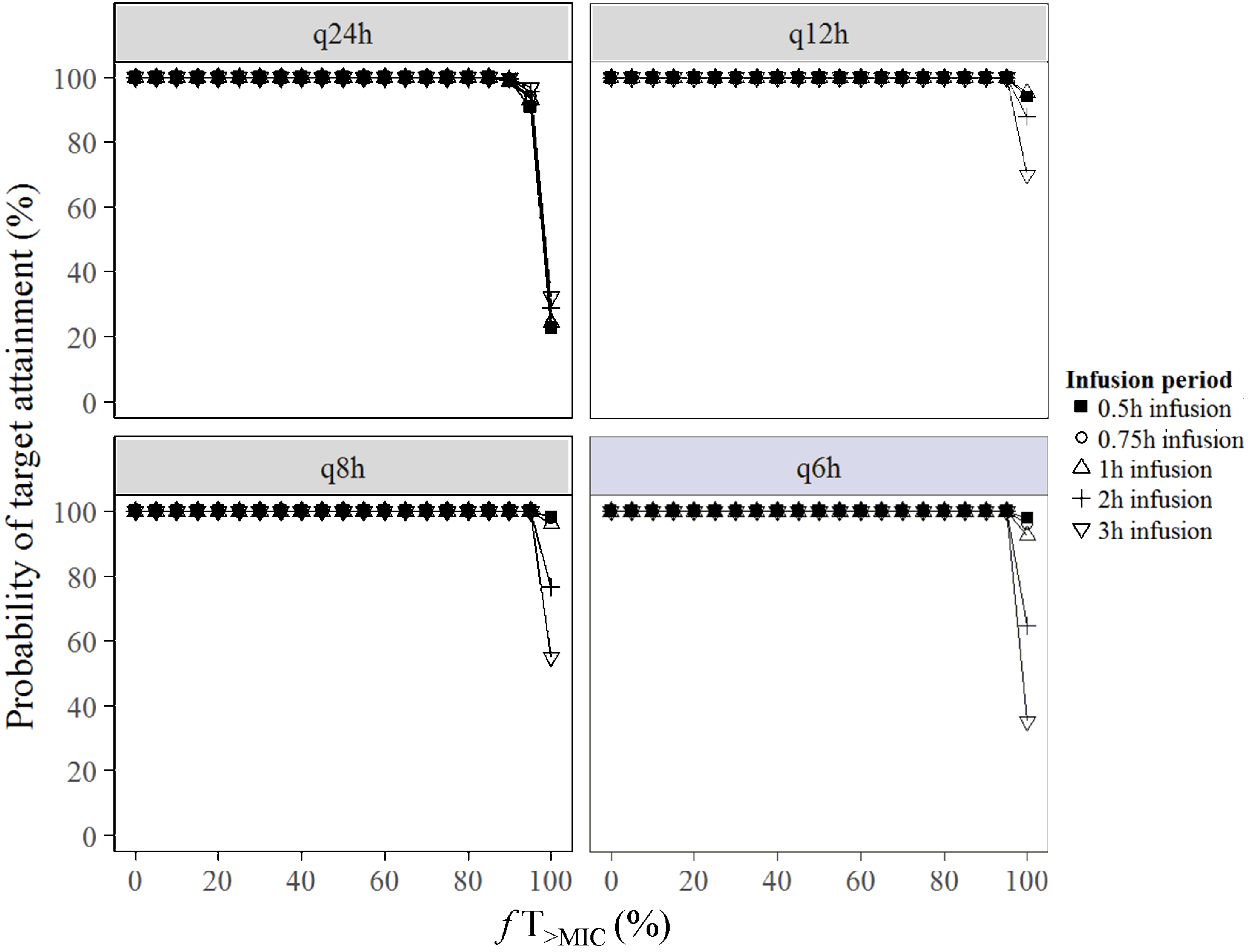

Figure S50. Probability of target attainment (PTA) of benapenem at %$fT_{>MIC}$ of 0% to 100% against *Serratia* under dose of 500 mg with different infusion time and dose interval.

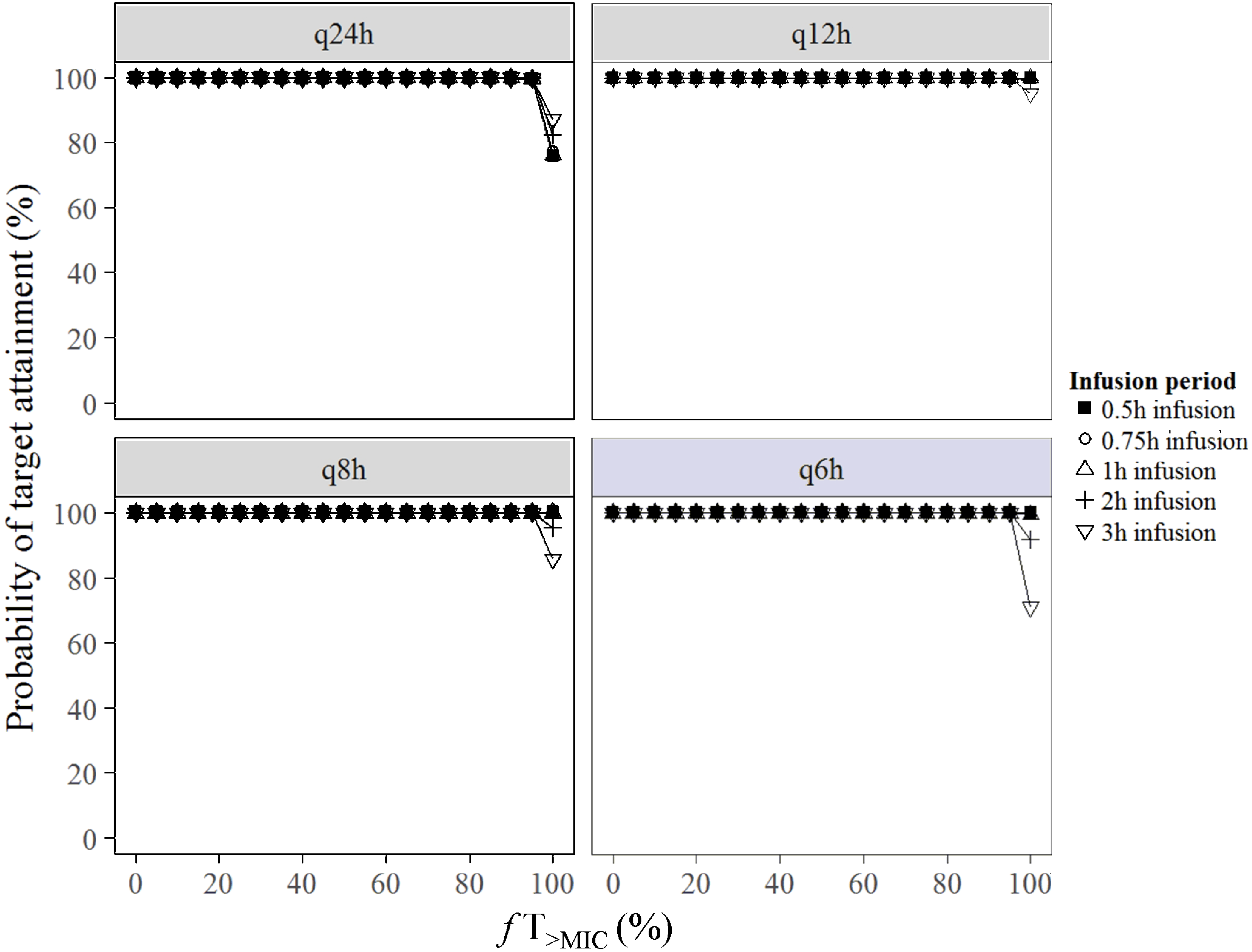

Figure S51. Probability of target attainment (PTA) of benapenem at %$fT_{>MIC}$ of 0% to 100% against *Serratia* under dose of 1000 mg with different infusion time and dose interval.

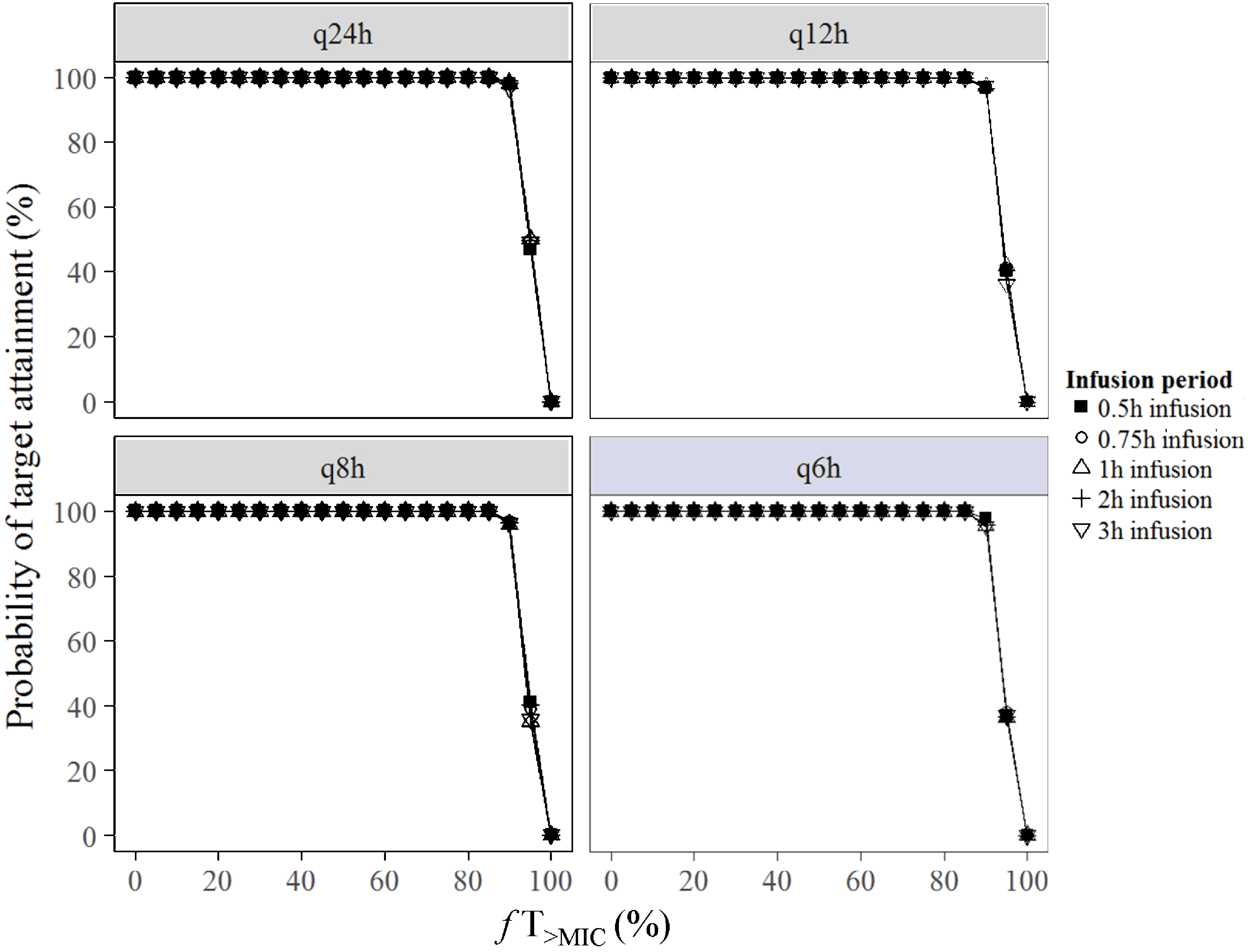

Figure S52. Probability of target attainment (PTA) of benapenem at %fT>MIC of 0% to 100% against *Shigella* under dose of 250 mg with different infusion time and dose interval.

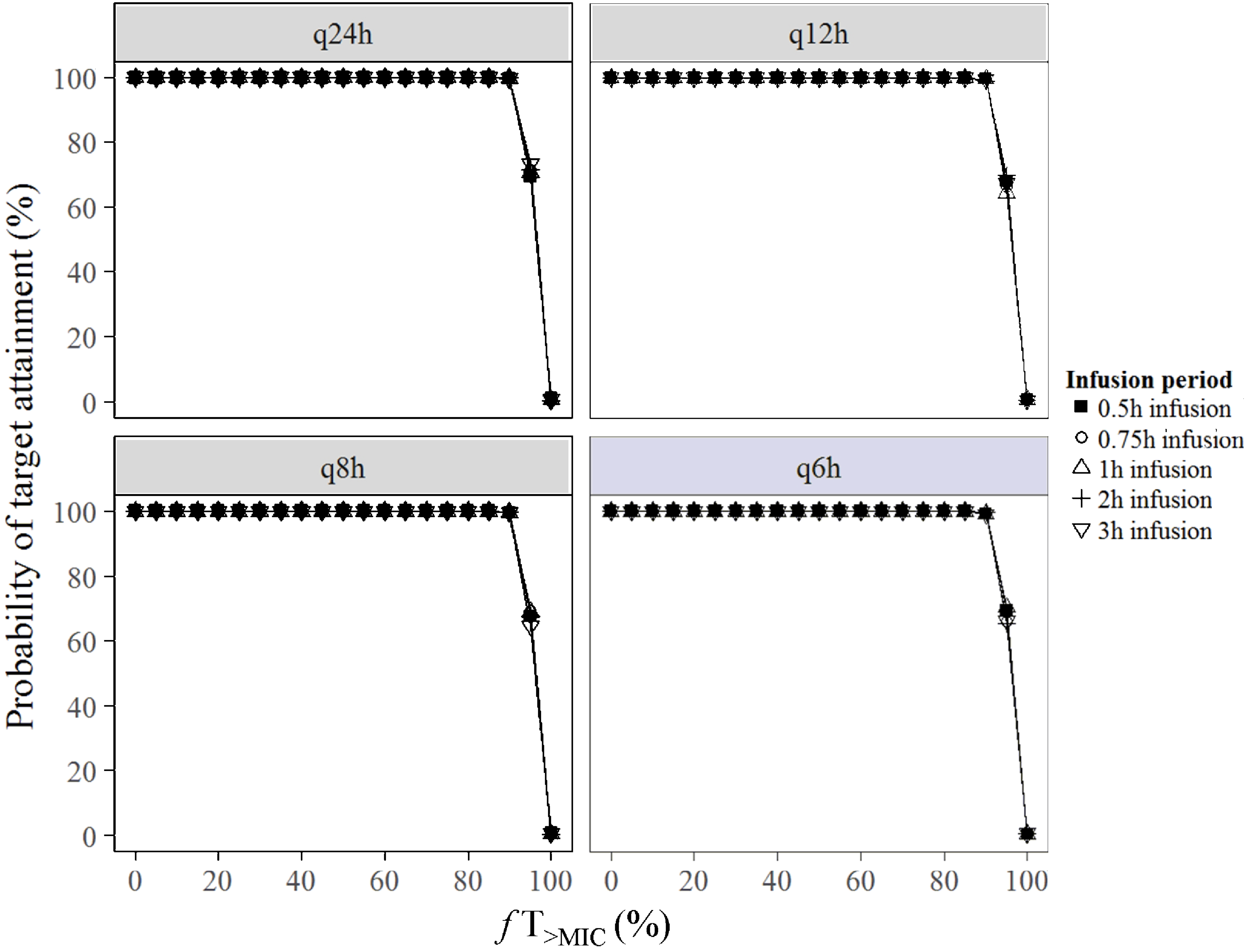

Figure S53. Probability of target attainment (PTA) of benapenem at %fT$_{>MIC}$ of 0% to 100% against *Shigella* under dose of 500 mg with different infusion time and dose interval.

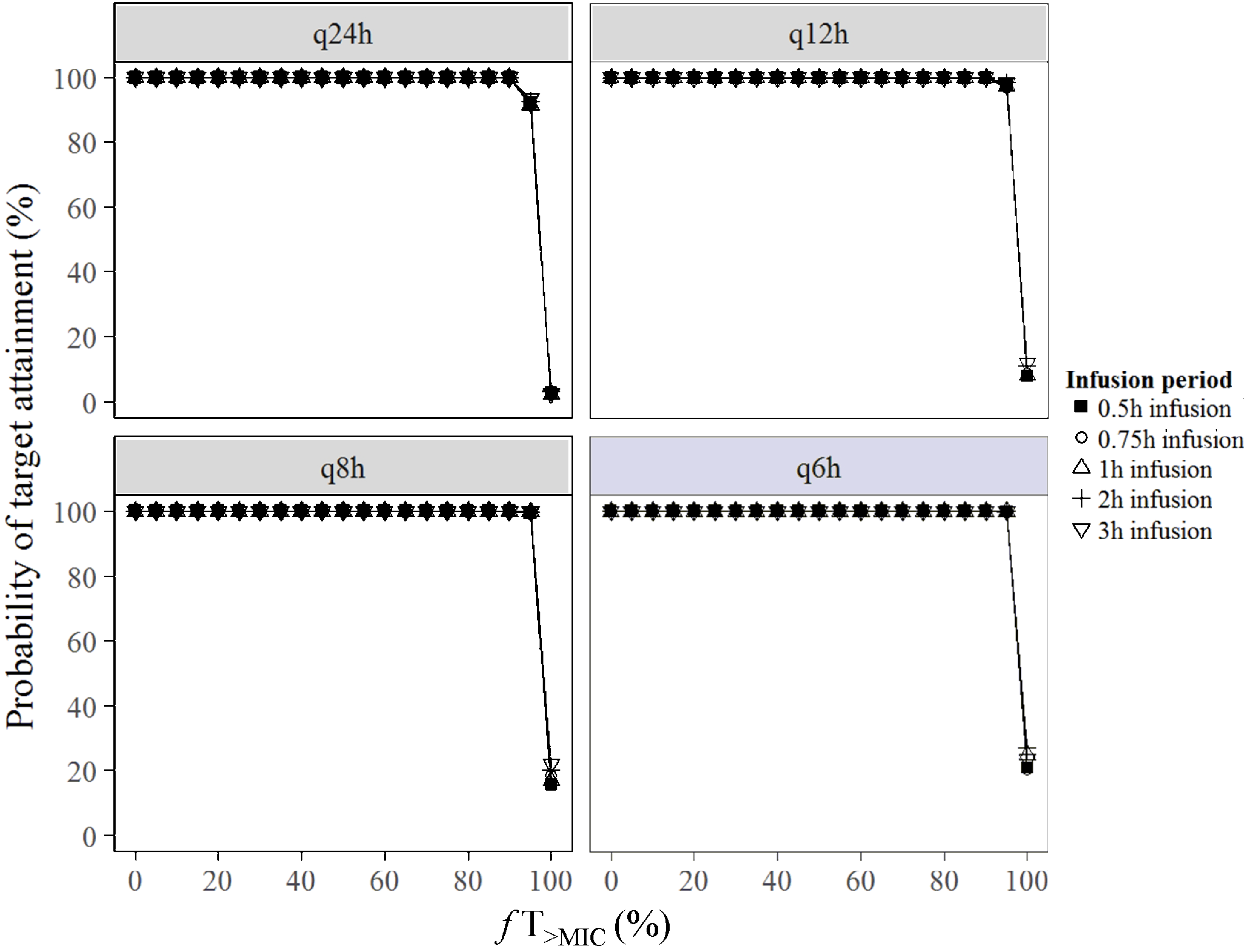

Figure S54. Probability of target attainment (PTA) of benapenem at $\%fT_{>MIC}$ of 0% to 100% against *Shigella* under dose of 1000 mg with different infusion time and dose interval.

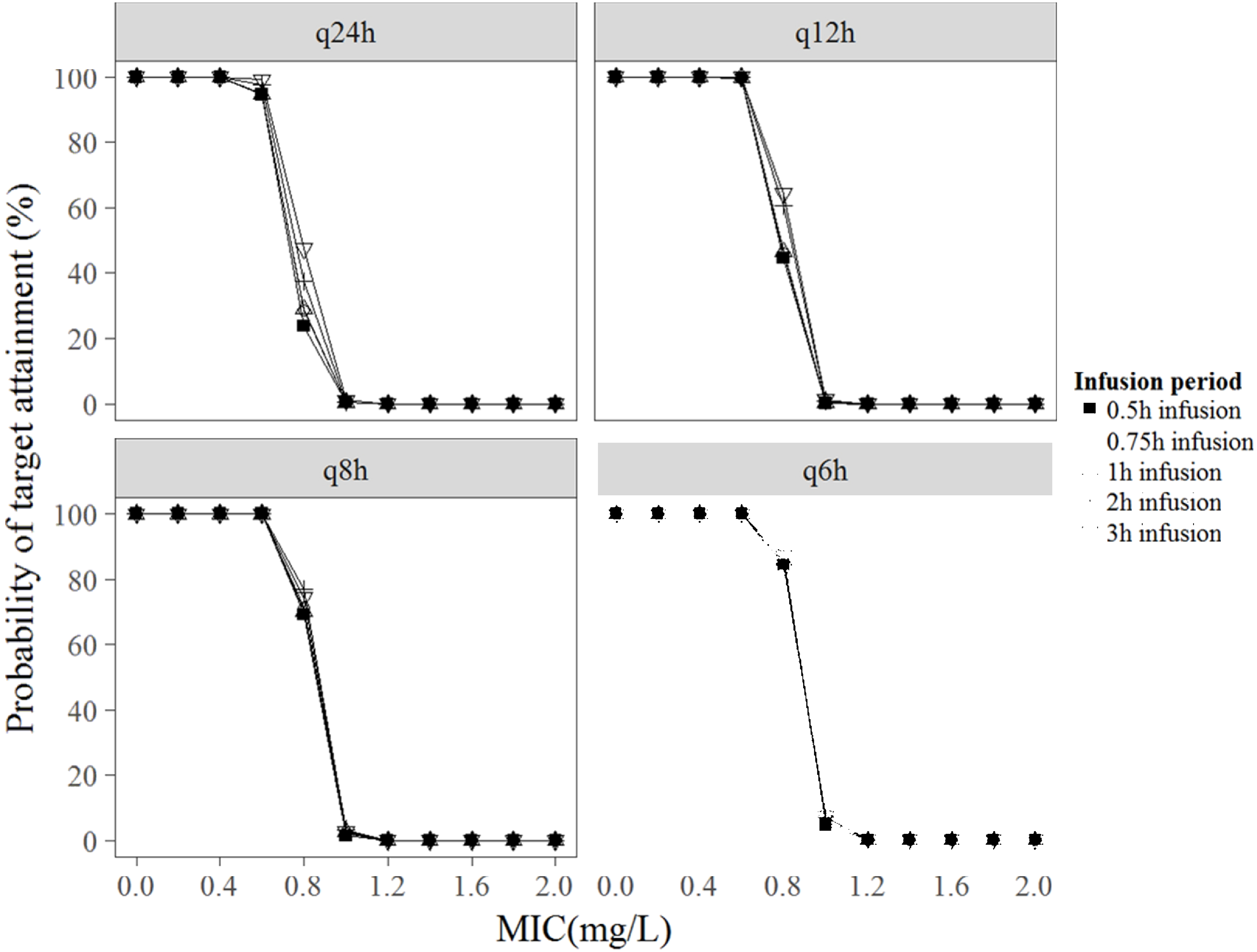

Figure S55. The relationship between probability of target attainment (PTA) and the MIC distribution of benapenem at %fT$_{>MIC}$=40% against enterobacteriaceae bacterial strains under dose of 250 mg with different infusion time and dose interval.

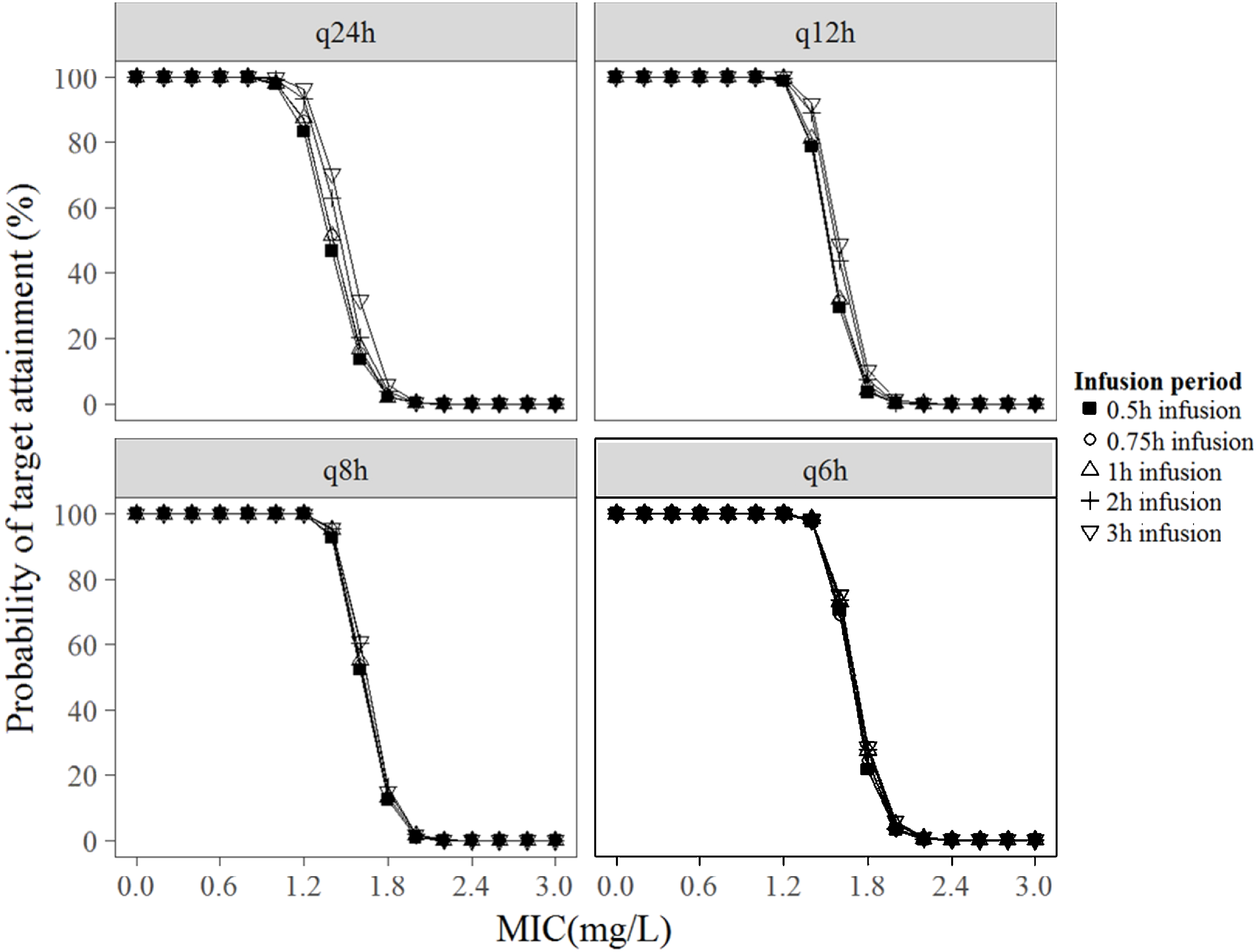

Figure S56. The relationship between probability of target attainment (PTA) and the MIC distribution of benapenem at %fT$_{>MIC}$=40% against enterobacteriaceae bacterial strains under dose of 500 mg with different infusion time and dose interval.

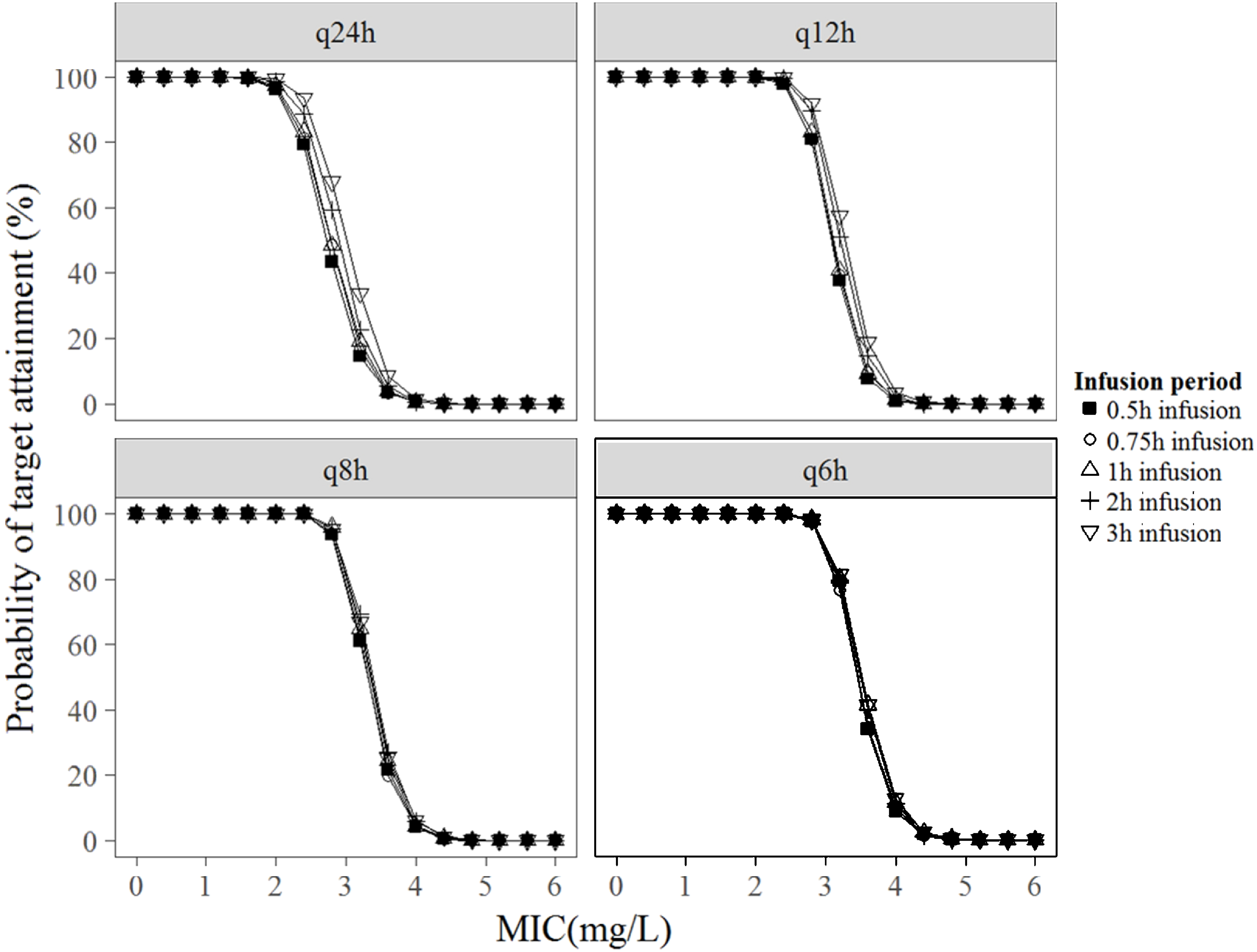

Figure S57. The relationship between probability of target attainment (PTA) and the MIC distribution of benapenem at $\%fT_{>MIC}$=40% against enterobacteriaceae bacterial strains under dose of 1000 mg with different infusion time and dose interval.

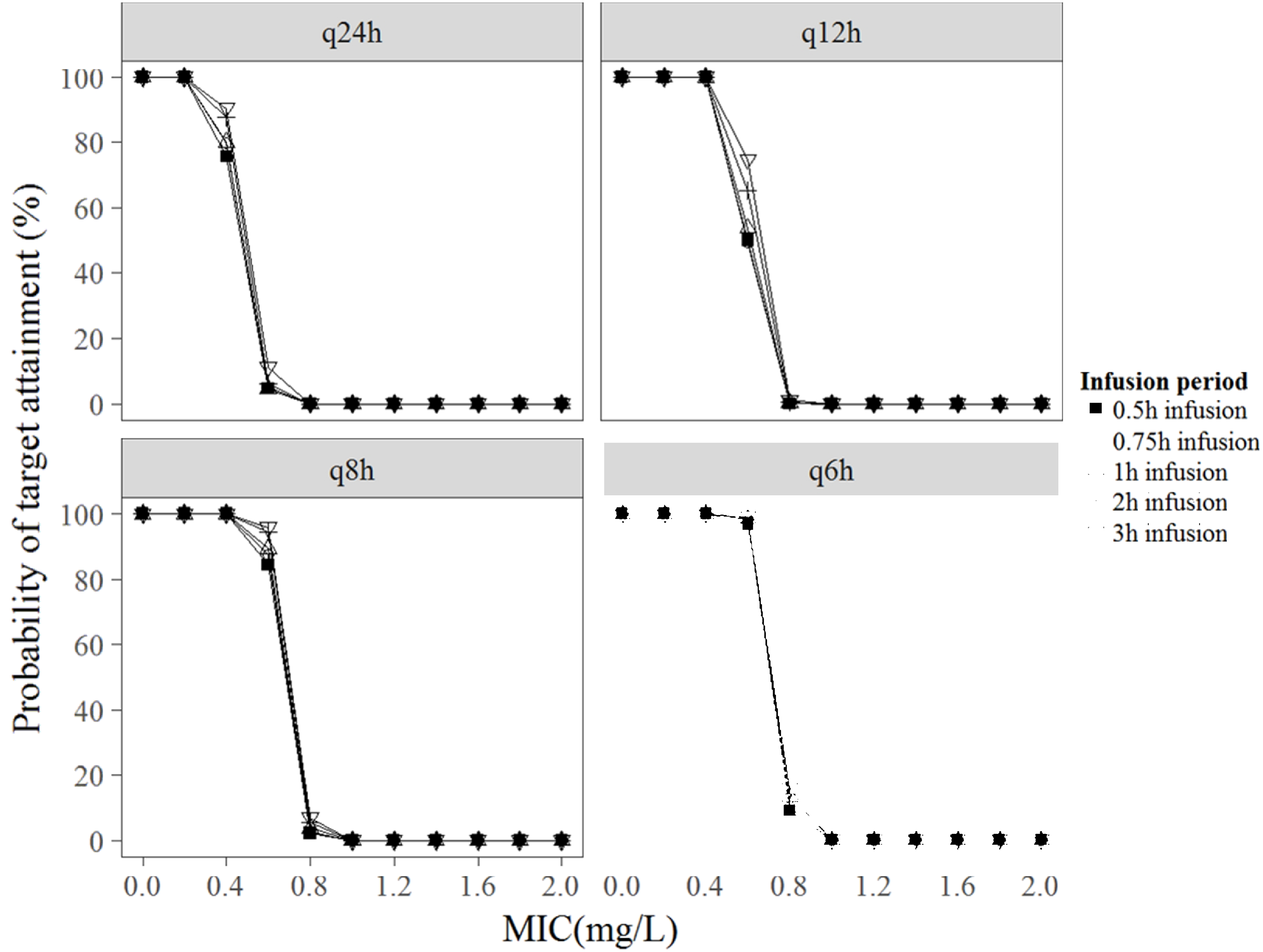

Figure S58. The relationship between probability of target attainment (PTA) and the MIC distribution of benapenem at %fT$_{>MIC}$=60% against enterobacteriaceae bacterial strains under dose of 250 mg with different infusion time and dose interval.

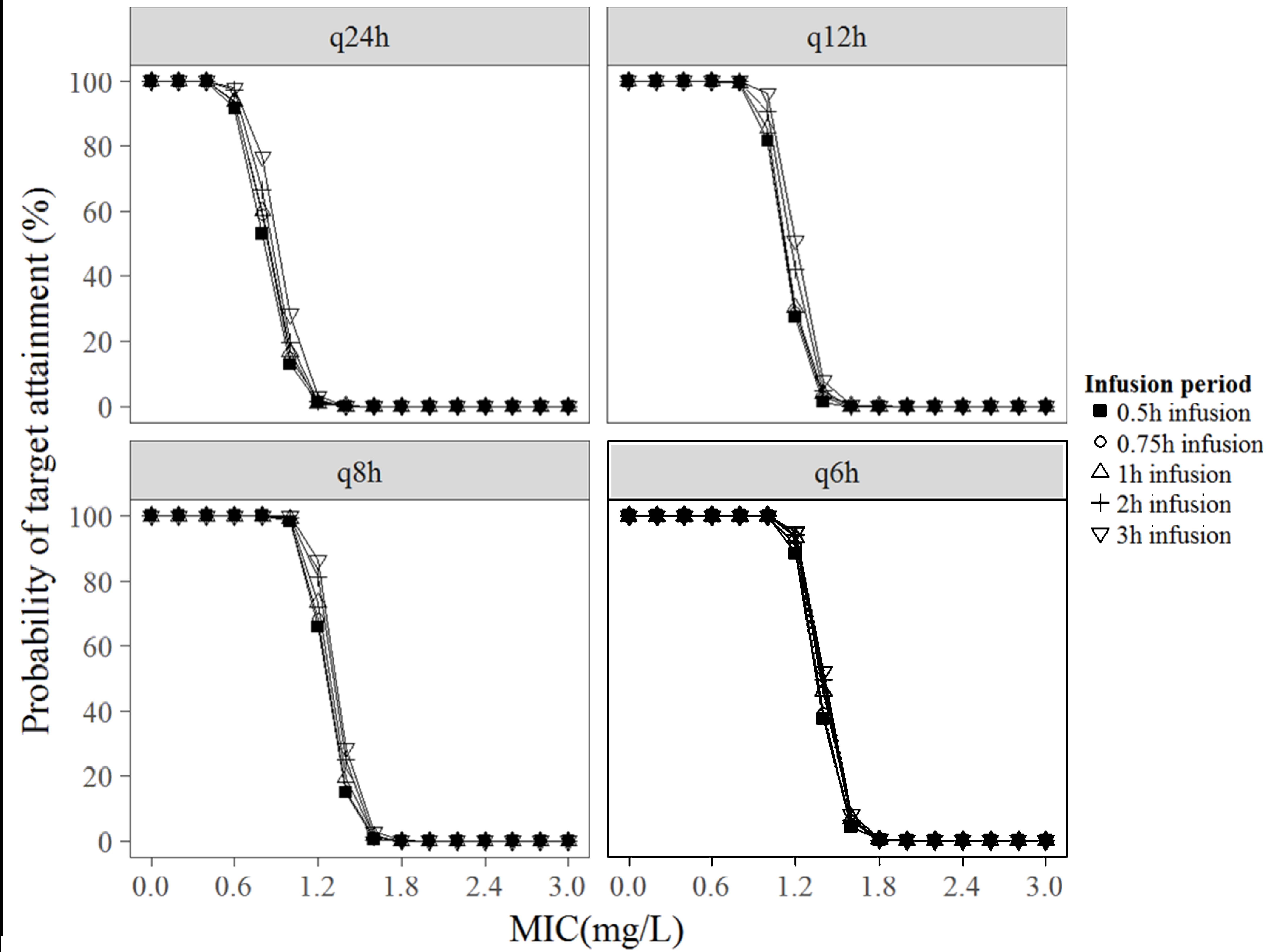

Figure S59. The relationship between probability of target attainment (PTA) and the MIC distribution of benapenem at %fT$_{>MIC}$=60% against enterobacteriaceae bacterial strains under dose of 500 mg with different infusion time and dose interval.

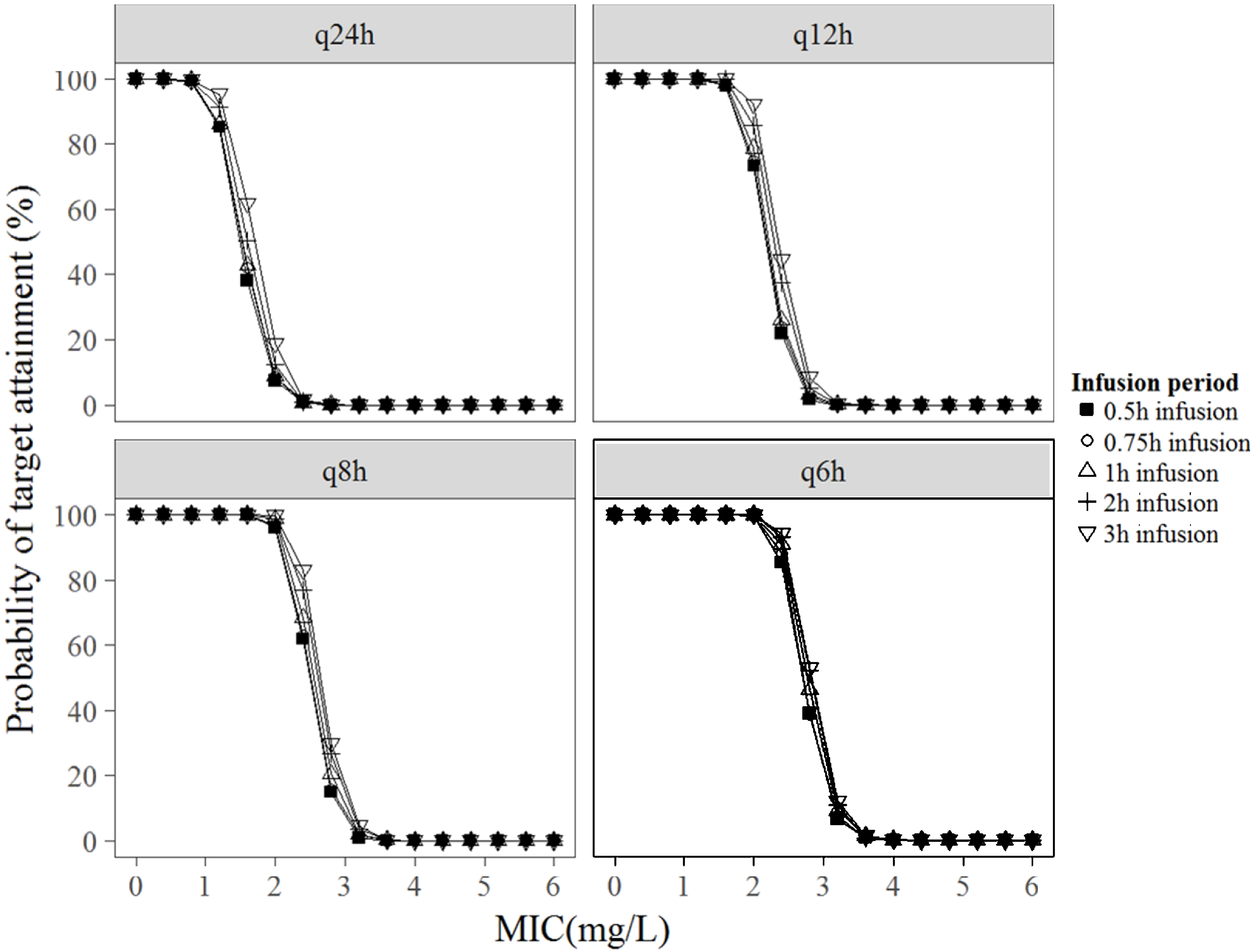

Figure S60. The relationship between probability of target attainment (PTA) and the MIC distribution of benapenem at $\%fT_{>MIC}$=60% against enterobacteriaceae bacterial strains under dose of 1000 mg with different infusion time and dose interval.
